# Affinity purification-mass spectrometry analysis of bcl-2 interactome identified SLIRP as a novel interacting protein

**DOI:** 10.1038/cddis.2015.357

**Published:** 2016-02-11

**Authors:** D Trisciuoglio, M Desideri, V Farini, T De Luca, M Di Martile, M G Tupone, A Urbani, S D'Aguanno, D Del Bufalo

**Affiliations:** 1 Department of Research, Advanced Diagnostics and Technological Innovation, Regina Elena National Cancer Institute, Rome, Italy; 2 Department of Experimental Medicine and Surgery, University of ‘Tor Vergata', Rome, Italy

## Abstract

Members of the bcl-2 protein family share regions of sequence similarity, the bcl-2 homology (BH) domains. Bcl-2, the most studied member of this family, has four BH domains, BH1–4, and has a critical role in resistance to antineoplastic drugs by regulating the mitochondrial apoptotic pathway. Moreover, it is also involved in other relevant cellular processes such as tumor progression, angiogenesis and autophagy. Deciphering the network of bcl-2-interacting factors should provide a critical advance in understanding the different functions of bcl-2. Here, we characterized bcl-2 interactome by mass spectrometry in human lung adenocarcinoma cells. *In silico* functional analysis associated most part of the identified proteins to mitochondrial functions. Among them we identified SRA stem–loop interacting RNA-binding protein, SLIRP, a mitochondrial protein with a relevant role in regulating mitochondrial messenger RNA (mRNA) homeostasis. We validated bcl-2/SLIRP interaction by immunoprecipitation and immunofluorescence experiments in cancer cell lines from different histotypes. We showed that, although SLIRP is not involved in mediating bcl-2 ability to protect from apoptosis and oxidative damage, bcl-2 binds and stabilizes SLIRP protein and regulates mitochondrial mRNA levels. Moreover, we demonstrated that the BH4 domain of bcl-2 has a role in maintaining this binding.

Mitochondrial-mediated apoptosis is significantly regulated by bcl-2 family members.^1^ This family is composed of pro- and anti-apoptotic proteins sharing at least one bcl-2 homology (BH) domain in common with bcl-2.^2^ Many studies have highlighted that the dysregulation of bcl-2 and other anti-apoptotic members is a distinguishing feature of cancer cells with respect to normal ones.^3^ Ours and other groups previously demonstrated that in addition to its critical role in regulating apoptosis, bcl-2 protein has also multiple apoptosis-independent functions, being involved in several phenomena including cell proliferation, tumor metastatization, angiogenesis and autophagy.^4, 5, 6^ Moreover, bcl-2 also regulates the cellular redox state interacting with the voltage-dependent anion channel 1 (VDAC1)^7^ and cytochrome *c* oxidase subunits Va (COX5A)^8, 9^ and prevents mitochondria from producing excessive reactive oxygen species (ROS).

Both the BH4 domain and the flexible loop domain, which links the BH4 domain to the BH3, are known to be significant for the anti-apoptotic activity of bcl-2.^10^ Although its conformation has not been completely elucidated, flexible loop domain is necessary for bcl-2 interaction with several proteins such as p53, JNK-1 and FKBP38.^11^ BH4 is also involved in several non-canonical bcl-2 functions. In this context, we demonstrated that removal of or mutations at the BH4 domain abrogate the ability of bcl-2 to induce Vascular Endothelial Growth Factor expression and transcriptional activity,^12^ reduce the interaction between bcl-2 and Hypoxia Inducible Factor-1*α* proteins and the capability of exogenous bcl-2 protein to localize in the nucleus^13^ and mediate inhibition of autophagy.^14^ It was also reported that BH4 domain mediates the interaction of bcl-2 with inositol 1,4,5-trisphosphate receptor.^10, 15^ Mutation of a tyrosine residue within BH4 domain is responsible of bcl-2-mediated cell cycle regulation.^16^ Furthermore, it was demonstrated that bcl-2 interacts via BH1 and BH4 domains with Mre11, inhibiting its activity and decreasing the repairing of clustered/complex DNA double-strand breaks.^17^ Recently, it was demonstrated that bcl-2 regulates autophagy also by binding the nutrient-deprivation autophagy factor-1, through both BH3 and BH4 domains^18^ and the phagophore-associated protein GABARAP via the three-residue segment adjacent to BH4.^19^

In this work, we investigated the network of bcl-2-interacting factors in order to identify novel putative bcl-2-binding proteins, which in turn should provide critical advances in understanding the regulation mechanism underlying different bcl-2 functions. By means of immune-affinity purification/mass spectrometry analysis, we identified 210 proteins in complex with bcl-2 in the H1299 human lung adenocarcinoma cell line stably overexpressing bcl-2 protein. Among the putative novel bcl-2-binding proteins, we identified SRA stem–loop interacting RNA-binding protein, SLIRP, a mitochondrial protein with a relevant role in regulating mitochondrial messenger RNA (mRNA) stability.^20^ After *in vitro* validation of bcl-2/SLIRP binding in cancer cell lines from different histotypes, we investigated the functional meaning of this novel interaction.

## Results

### NanoLiquid chromatography tandem mass spectrometry (nLC-MS/MS) identification and *in silico* analysis of proteins interacting with bcl-2

**Figure 1:**
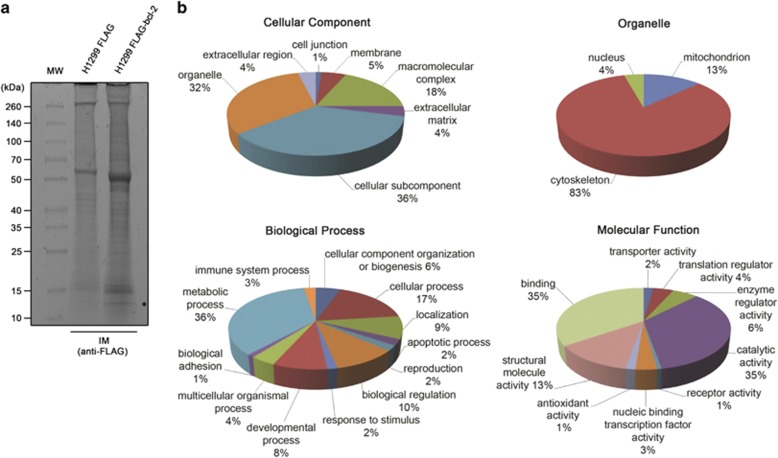
Functional analysis of bcl-2-interacting proteins. (**a**) Immunocomplexes (IMs) separation by SDS-PAGE. H1299 control (H1299 FLAG) and bcl-2-overexpressing (H1299 FLAG-bcl-2) cells were immunoprecipitated with anti-FLAG antibody. IMs were recovered, separated by SDS-PAGE and visualized by Coomassie staining. Lines were cut in 12 pieces, digested by trypsin and analyzed by nLC-MS/MS. Asterisk on the image indicates the band running at ~12 kDa in which SLIRP was identified. (**b**) Functional analysis by PANTHER classification system. Pie charts showing protein classification by cellular component, organelle, biological process and molecular function. The percentage of annotated categories is reported

Bcl-2 immunocomplexes (IMs) obtained from total protein extracts of H1299 stably overexpressing bcl-2 wild-type protein fused to FLAG peptide (H1299 FLAG-bcl-2) were separated by SDS-PAGE gel and visualized by Coomassie staining (Figure 1a). IMs obtained from H1299 cells transfected with the FLAG-empty vector were used as control. Twelve bands for each lane were excised from gel, subjected to trypsin digestion and resulting peptides were extracted for nLC-MS/MS analysis. A total of 210 proteins were identified with false discovery rate <1% in FLAG-bcl-2 IM after subtracting the proteins found in the IM control experiments with FLAG-empty vector (Supplementary Table 1). The list of proteins was used as input file for PANTHER classification system to classify them by different functional categories (Figure 1b). A relevant number of the proteins, representing 32% of the pie chart, had an organelle subcellular localization and among them a fraction corresponding to 13% was associated to mitochondria. Most part of the identified proteins (36% of the pie chart) was associated to metabolic process by biological process classification. In accordance with this classification, 35% of the pie chart displaying the classified molecular function was represented by proteins with catalytic activity. Moreover, an equivalent fraction was represented by proteins showing binding function. Molecular and cellular functions were also investigated by Ingenuity Pathway Analysis software (IPA; Supplementary Table 4). Results showed that proteins covered a broad range of functional categories, being classified into five molecular and cellular functions. The multiple categories represented by the identified proteins led to the construction of five top-ranked networks with distinct associated functions (Supplementary Table 5). In summary, the two most significant networks were associated to protein synthesis, gene expression, carbohydrate metabolism and RNA-posttranscriptional modification. Identified proteins with structural and transport functions led to the generation of networks associated to cellular assembly and organization, cellular compromise and molecular transport. The fourth ranked network was more focused on functions involved in biochemical aspects of the cell, being associated to free radical scavenging, small-molecule biochemistry and metabolic disease. Cellular assembly and organization was also associated to the fifth ranked network, where the expected function cell death and survival was also reported. On the other hand, the top tox list generated by IPA showed more homogeneity among the reported categories, mostly focused on mitochondrion functions (Supplementary Table 6). In accordance with these findings, mitochondrial dysfunction and oxidative phosphorylation were highlighted by IPA among top canonical pathways (Supplementary Table 7). In agreement with previously published papers,^7, 9^ in this study we have identified VDAC1 and COX5A as bcl-2 interactors among proteins associated to mitochondrial dysfunction (Supplementary Table 8).

### *In vitro* validation of bcl-2 binding to SLIRP

Considering that bcl-2 main site of action and localization lies on the outer mitochondrial membrane and its protective mechanism aims at preserving mitochondrial membrane integrity,^9^ our validation of novel bcl-2-binding proteins started from those strictly correlated to mitochondria. In particular, we focused our attention on SLIRP (Supplementary Table 8). SLIRP is predominantly associated to mitochondria where, in combination with leucine-rich pentatricopeptide repeat-containing (LRPPRC), is involved in maintaining mitochondrial mRNA homeostasis.^20, 21^

**Figure 2:**
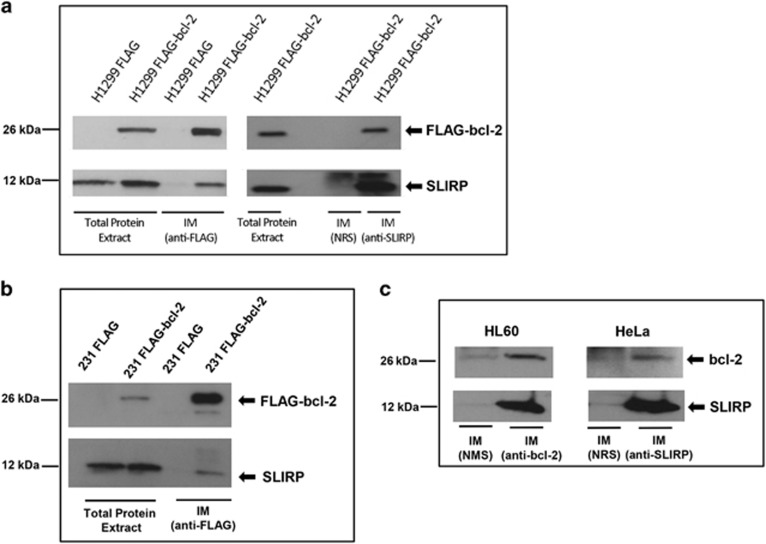
*In vitro* validation of bcl-2/SLIRP interaction. (**a**) Bcl-2 and SLIRP co-immunoprecipitation in H1299 control (H1299 FLAG) and bcl-2-overexpressing (H1299 FLAG bcl-2) cells. Western blot analysis of FLAG-bcl-2 and SLIRP proteins from immunocomplexes (IMs) obtained using anti-FLAG or anti-SLIRP antibodies. (**b**) Bcl-2 and SLIRP co-immunoprecipitation in MDA-MB 231 control (231 FLAG) and bcl-2-overexpressing (231 FLAG-bcl-2) cells. Western blot analysis of FLAG-bcl-2 and SLIRP proteins from IM obtained using anti-FLAG antibody. (**c**) Bcl-2 and SLIRP co-immunoprecipitation in HL60 and HeLa cell lines. Western blot analysis of bcl-2 and SLIRP proteins from IM obtained in HL60 cells using endogenous anti-bcl-2 antibody and in HeLa cells using endogenous anti-SLIRP antibody. (**a**, **c**) Normal rabbit serum was used as control. (**c**) Normal mouse serum (NMS) was used as control

Co-immunoprecipitation of SLIRP with bcl-2 was strengthened in new prepared FLAG-bcl-2 IM, analyzed by Western blotting using anti-SLIRP antibody (Figure 2a). A further validation was provided by the analysis of IM obtained using endogenous SLIRP as bait. Western blot analysis of SLIRP IM using anti-FLAG antibody confirmed the co-immunoprecipitation of bcl-2 (Figure 2a). As SLIRP expression has been demonstrated both in breast cancer cell lines and primary tissues (http://www.proteinatlas.org), we extended the results obtained in the lung adenocarcinoma model to a breast adenocarcinoma cell line with low level of endogenous bcl-2, such as MDA-MB-231. To this purpose, we established a MDA-MB-231 cell line stably overexpressing bcl-2 wild-type protein fused to FLAG sequence (MDA-MB-231 FLAG-bcl-2). As observed in lung cancer model, co-immunoprecipitation of bcl-2-fused protein and SLIRP was observed in IM prepared from total extract of MDA-MB-231 FLAG-bcl-2 (Figure 2b). Co-immunoprecipitation of SLIRP with endogenous bcl-2 was observed in other cell lines, such as acute promyelocytic leukemia HL60 and human epithelial cervix adenocarcinoma HeLa cells (Figure 2c).

**Figure 3:**
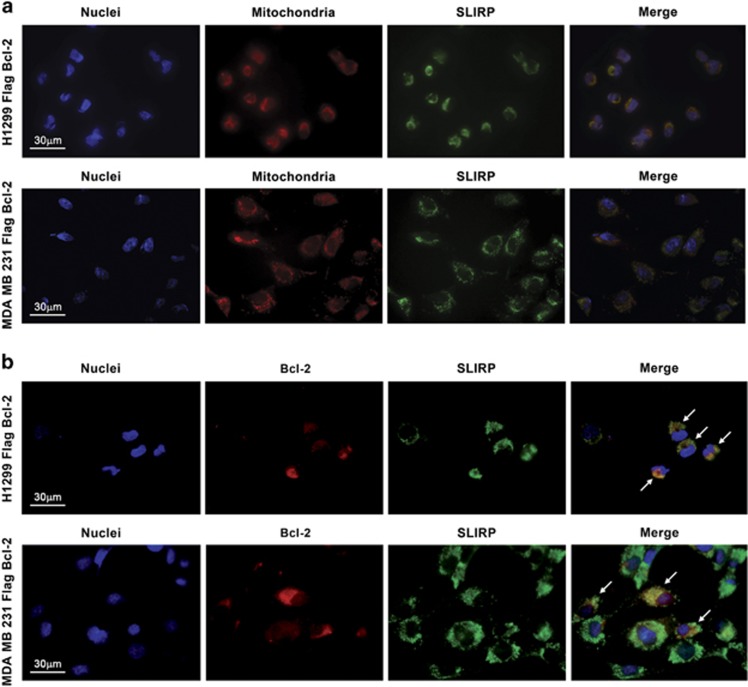
Immunofluorescence analysis. (**a**) Immunostaining analysis showing the co-localization of antibody-stained SLIRP (green) with Mitotracker-labeled mitochondria (red) in H1299 and MDA-MB-231 cell lines overexpressing bcl-2. (**b**) Immunostaining analysis showing partial co-localization between endogenous SLIRP (green) and bcl-2 (red) in H1299 and MDA-MB-231 cell lines overexpressing bcl-2; arrows indicate bcl-2/SLIRP co-localization. **a**, **b**: Representative images from one experiment are shown. Detailed images of single cell acquired with × 100 objective are reported in Supplementary Information (Supplementary Figure 1)

We next investigated SLIRP subcellular localization in immunofluorescence experiments (Figure 3 and Supplementary Figure 1). In accordance with previously reported experiments conducted in HeLa cells,^21^ SLIRP is predominantly localized at mitochondria in both H1299 and MDA-MB-231 cell lines (Figure 3a). Interestingly, partial co-localization of bcl-2 and SLIRP was observed in both cell lines overexpressing FLAG-bcl-2 (Figure 3b). These results, confirming the bcl-2/SLIRP binding, prompted us to investigate the functional meaning of bcl-2/SLIRP interaction.

### Functional meaning of bcl-2/SLIRP interaction

**Figure 4:**
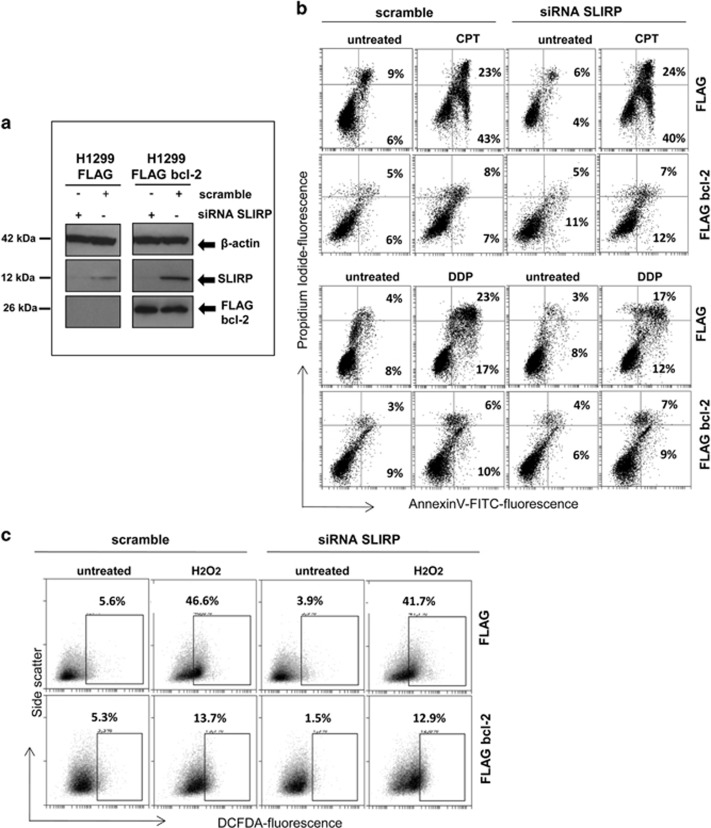
Evaluation of apoptosis and ROS production in H1299 cells. (**a**) Western blot analysis of SLIRP downregulation in H1299 cells. SLIRP silencing was achieved by using a targeted pool of siRNA (siRNA SLIRP) in control (H1299 FLAG) and bcl-2-overexpressing (H1299 FLAG bcl-2) cells. A pool of siRNA against scramble target sequence was used as control (scramble). *β*-Actin was used as loading control. (**b**) Apoptotic cells by Annexin V/PI staining in H1299 control and bcl-2-overexpressing cells transfected with siRNA scramble or siRNA SLIRP and exposed to 0.5 *μ*M camptothecin (CPT) for 48 h or 20 *μ*M cisplatin (DDP) for 24 h. The percentage of Annexin V^+^/PI^−^ (early apoptotic cells) and Annexin V^+^/PI^+^(late apoptotic cells) is shown. (**c**) ROS production determined in H1299 control and bcl-2-overexpressing cells transfected with siRNA scramble or siRNA SLIRP and exposed to H_2_O_2_ for 30 min. Percentage of 5–6-chloromethyl-20,70-dichlorofluorescindiacetate (DCFDA)-positive cells is shown. (**b** and **c**) Images from a representative experiment are shown

**Figure 5:**
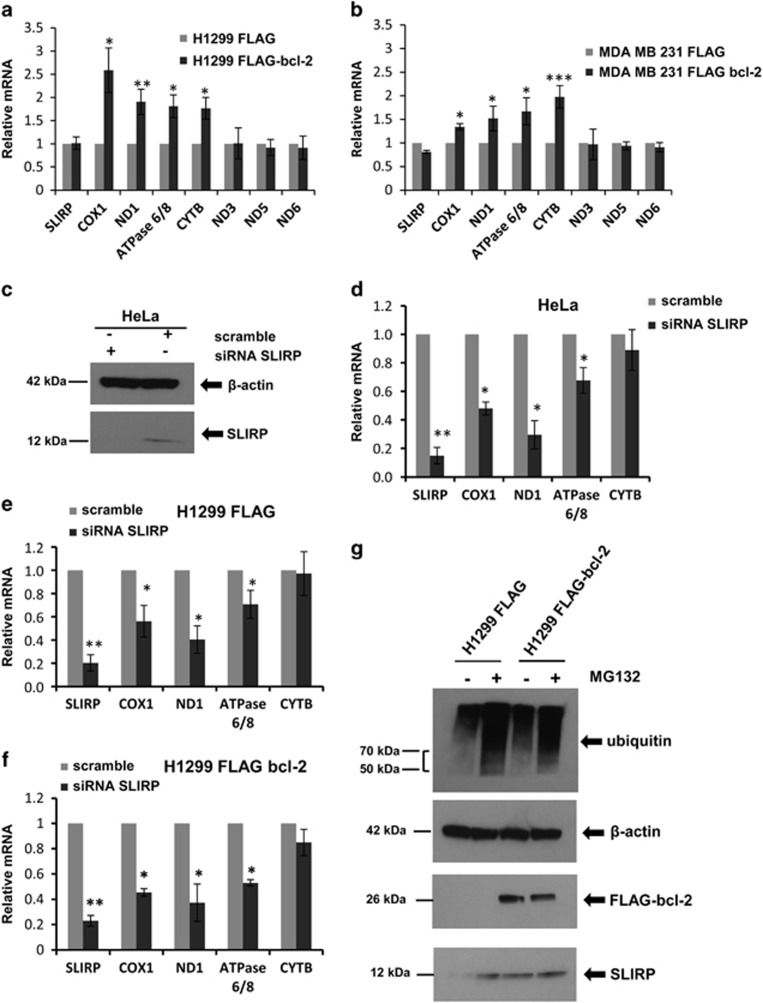
Analysis of mitochondrial mRNA levels. Mitochondrial mRNA levels determined by qRT-PCR in H1299 (**a**) and MDA-MB 231 (**b**) control and bcl-2-overexpressing cell lines. Values are expressed as means of ratio±S.E.M., where ‘ratio' was calculated considering bcl-2-overexpressing cells versus control cells. *P-*values are calculated between control and bcl-2-overexpressing cells. (**c**) Western blot analysis of SLIRP downregulation in HeLa cells. SLIRP silencing was achieved by using a targeted pool of siRNA (siRNA SLIRP). A pool of siRNA against scramble target sequence was used as control (scramble). (**d–f**) Mitochondrial mRNA levels determined by qRT-PCR in (**d**) HeLa, (**e**) H1299 control (H1299 FLAG) and (**f**) H1299 bcl-2-overexpressing (H1299 FLAG bcl-2) cells transfected with siRNA scramble or siRNA SLIRP. Values are expressed as means of ratio±S.E.M., where ‘ratio' was calculated considering siRNA SLIRP versus siRNA scramble cells. (**g**) Western blot analysis of FLAG bcl-2, SLIRP and poly-ubiquitinated proteins from H1299 control and bcl-2-overexpressing cells in the presence or absence of 10 *μ*M MG132 for 6 h. (**c** and **g**) *β*-Actin was used as control loading. (**a, b, d–f**) *P<0.05; **P<0.01; ***P<0.001

The possible involvement of SLIRP in bcl-2-mediated functions was explored in H1299 cells. To this purpose, the effect of SLIRP silencing on the ability of bcl-2 to protect from apoptosis and ROS production was evaluated in both control and bcl-2-overexpressing cells. SLIRP silencing was achieved by using a pool of small interfering RNA (siRNA) and the downregulation of SLIRP was observed both at protein (Figure 4a) and mRNA levels (Figures 5c, e and f). We evaluated the response of cells to camptothecin (CPT) and cisplatin (DDP), drugs able to activate the apoptotic program in several experimental models^22, 23^ by performing an Annexin V/propidium iodide (PI) staining that allows the discrimination of viable cells (Annexin V^−^/PI^−^), early apoptotic (Annexin V^+^/PI^−^) and late apoptotic/necrotic cells (Annexin V^+^/PI^+^). As shown in Figure 4b, CPT treatment induced ~40% of early and ~20% of late apoptotic/necrotic events in H1299 control cells, whereas in accordance with the known anti-apoptotic function of bcl-2, it induced apoptosis or necrotic cell death in less than 10% in bcl-2-overexpressing cells. Notably, SLIRP silencing did not affect apoptosis induced by CPT in H1299 control cells, as well as the protective role of bcl-2 in bcl-2-overexpressing cells (Figure 4b). Similar results were observed after DDP treatment that induced ~20% of early and ~20% of late apoptotic/necrotic events in H1299 control cells, whereas, as expected, bcl-2 overexpression protected cells from apoptosis induction. Both apoptosis induction by DDP in H1299 control cells and the protective role of bcl-2 were not affected by SLIRP silencing.

Next, we evaluated the possible involvement of SLIRP on bcl-2-mediated antioxidant-function. Figure 4c shows cytofluorimetric analysis of ROS production after treatment with H_2_O_2_ in H1299 control and bcl-2-overexpressing cells upon SLIRP silencing. As expected, bcl-2 overexpression determined a decrement of ROS level in respect to control cells, whereas SLIRP silencing was not able to influence the protective role played by bcl-2.

As it is reported that knockdown of SLIRP^24, 25^ decreases mitochondrial mRNA levels,^20^ we determined the effect of bcl-2 overexpression on the levels of seven transcripts, representative of mitochondrial mRNA and part of respiratory chain complexes I, III, IV and V. As reported in Figure 5a, a significant increment of the levels of cytochrome *b* (CYTB), cytochrome *c* oxidase (COX1), NADH-ubiquinone oxidoreductase chain 1 (ND1), ATP synthase (ATPase 6/8) was observed after bcl-2 forced expression in H1299 cells. Similarly, the increment of these four tested transcripts was also observed in breast cancer cells overexpressing bcl-2 (Figure 5b), even if at different extent. On the contrary, the transcript levels of NADH-ubiquinone oxidoreductase chain 3 (ND3), NADH-ubiquinone oxidoreductase chain 5 (ND5) and NADH-ubiquinone oxidoreductase chain 6 (ND6) were not modulated by bcl-2 overexpression in both lung and breast cancer cell lines. Next, the level of CYTB, COX1, ND1 and ATPase 6/8 transcripts was evaluated after SLIRP silencing in control and bcl-2-overexpressing cells. In accordance with published data,^20^ SLIRP silencing (Figure 5c) determined a remarkable downregulation of COX1, ND1 and ATPase 6/8in HeLa cells (Figure 5d), whereas a not significant decrement of CYTB was observed. As reported in Figures 5e and f, in H1299 cells, SLIRP knockdown was responsible of the significant downregulation of COX1, ND1 and ATPase 6/8, both in the absence and presence of bcl-2 overexpression and of the not significant minor modulation of CYTB transcript. As shown by Western blot analysis reported in Figure 5g, in bcl-2-overexpressing cells, an increment of SLIRP protein level, not associated to a significant modulation of its transcript (Figures 5a and b), was observed.

To evaluate a possible involvement of bcl-2 on SLIRP protein stabilization, which in turn may positively affects the level of mitochondrial mRNA, the effect of the proteasome inhibitor MG132 on SLIRP protein level was evaluated. As reported in Figure 5g in the presence of MG132, SLIRP accumulated in H1299 control cells, whereas the increment of SLIRP level was not evident in H1299 bcl-2-overexpressing cells, thus indicating a minor portion of SLIRP protein prone to be eliminated by proteasome after bcl-2 overexpression.

**Figure 6:**
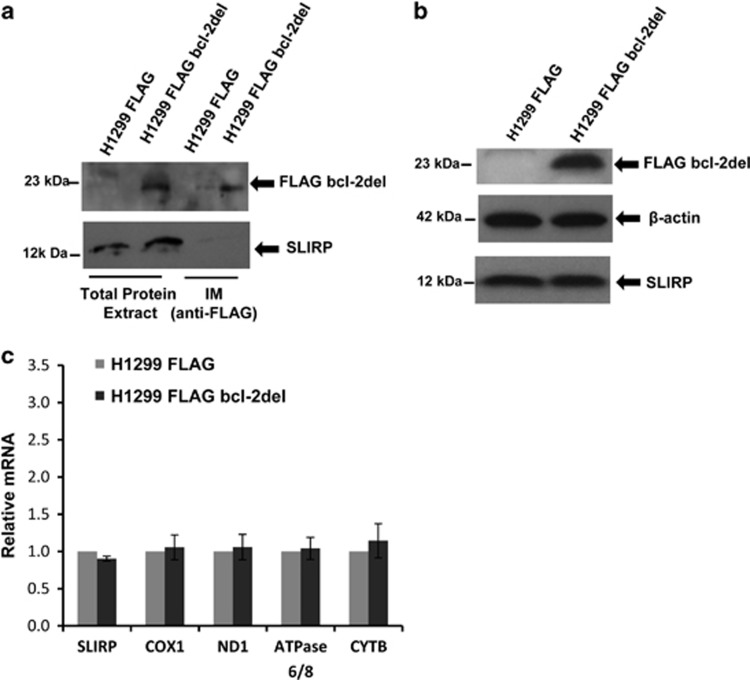
BH4 domain involvement in bcl-2 binding to SLIRP. (**a**) Western blot analysis of immunocomplexes (IMs) obtained using anti-FLAG antibody in H1299 control cells (H1299 FLAG) and cells overexpressing bcl-2 protein deleted of BH4 domain (H1299 FLAG bcl-2del). (**b**) Western blot analysis of SLIRP and FLAG bcl-2del expression from H1299 FLAG and H1299 FLAG bcl-2del. *β*-Actin was used as control loading. (**c**) Mitochondrial mRNA levels determined by qRT-PCR in H1299 FLAG and H1299 FLAG bcl-2del cells. Values are expressed as means of ratio±S.E.M., where ‘ratio' was calculated considering FLAG bcl-2del cells versus control cells

BH4 domain is one of the principal domains for the full multifunctional ability of bcl-2, and it appears to be mainly involved in the interaction of bcl-2 with other proteins not belonging to bcl-2 family.^12, 13, 14, 15, 16, 17, 18, 19^ To deeper investigate the mechanism of bcl-2-mediated regulation of mitochondrial mRNA, the role of BH4 domain in bcl-2/SLIRP interaction was evaluated. For this purpose, the H1299 cell line overexpressing the mutant form of bcl-2 protein, lacking the BH4 domain (corresponding to the 1–36 amino-acid sequence) and fused to FLAG sequence at N-terminal (H1299 FLAG bcl-2del) was generated. As shown by Western blotting analysis of IMs (Figure 6a), the deletion of BH4 domain reduced the affinity of bcl-2 protein in the binding of SLIRP, thus suggesting that BH4 domain may mediate the interaction between bcl-2 and SLIRP. In accordance with this evidence, the level of SLIRP protein was not increased in H1299 FLAG bcl-2del extract with respect to that of controls cells (Figure 6b). No significant variation in mRNA expression was observed in cells overexpressing the bcl-2-deleted protein with respect to control cells (Figure 6c), underlining the role of BH4 in bcl-2 contribution to mitochondrial transcripts level modulation.

## Discussion

In this work, we investigated the bcl-2 interactome in human lung adenocarcinoma cells by single-step affinity purification coupled to mass spectrometry protein identification. This approach is a powerful large-scale throughput platform useful to generate protein–protein interaction networks,^26, 27^ although some limitations may derive from the production of false positives associated to the generation of large data set. To overcome these limitations, validation experiments, such as co-localization, co-immunoprecipitation and functional assays, were performed. In the effort to functionally annotate the proteins identified in complex with bcl-2, which include direct interactors but also proteins with varying degrees of interconnectivity, the bioinformatics supports Panther and IPA were employed. As expected among the most significant networks generated by IPA there was the cell death- and survival-associated function, which is in line with the canonical bcl-2 and bcl-2 homolog activity, consisting of the inhibition of death by regulating mitochondrial outer membrane permeabilization.^28^ In accordance with this knowledge, mitochondrial associated functions such as mitochondrial dysfunction and oxidative phosphorylation were highlighted by IPA among top canonical pathways. Among proteins identified in this study and associated to mitochondrial function, VDAC1 and COX5A have been already reported as bcl-2-interacting proteins able to regulate cellular redox state.^7, 9^ Other known bcl-2 interaction proteins were not found, because of the specific conditions of experimental procedure of IM preparation (i.e., condition of growth, used buffer, cross-linking), separation by SDS-PAGE and mass spectrometry analysis used in this study.

On the basis of strengthened evidences of bcl-2 involvement in mitochondria associated functions, as intrinsic apoptotic pathway and ROS production, we selected for the validating phase SLIRP, a novel putative bcl-2-interacting protein with known relevant impact on mitochondria maintaining. SLIRP is widely expressed in human normal and cancer tissues and in cancer cell lines with different histotype, including lung and breast carcinoma (http://www.proteinatlas.org).^21^ It was first identified for its ability to bind a specific steroid receptor RNA activator sequence, acting as repressor of nuclear receptor signaling.^21^ The role of SLIRP in cancer development has not been deeply investigated to date, and although few data are reported regarding the role played by SLIRP in regulating hormone-dependent cancer,^21^ a better characterization of mitochondrial SLIRP function, being mainly localized at this organelle, is available in the literature.^20, 29^ *In silico* screen for assembly, maturation and regulatory factors of oxidative phosphorylation machine identified SLIRP as player of mitochondrial RNA homeostasis and the mechanism of SLIRP regulation of mitochondrial encoded mRNA has been characterized.^24^ SLIRP is part of a ribonucleoprotein complex that regulates posttranscriptional gene expression in mitochondria.^29^ In this contest, SLIRP interacts with LRPPRC and knockdown of either SLIRP^24^ or LRPPRC^25^ results in similar decreases in mitochondrial mRNA levels, indicating that the LRPPRC/SLIRP complex has a specific role in mRNA maturation or stabilization after transcription in mitochondria. In particular, it was demonstrated that the LRPPRC/SLIRP complex suppresses the mRNA degradation and promotes polyadenylation in human mitochondria.^20^ Interestingly, LRPPRC is also reported to inhibit autophagy by binding beclin 1 and bcl-2.^30^ These evidences prompted us to deeper investigate the interaction of bcl-2 with SLIRP, which was not reported before and which could reveal new bcl-2- and SLIRP-associated functions. Bcl-2 binding to SLIRP was validated by co-immunoprecipitation and co-localization experiments both in lung cancer and breast cancer cell models forced to overexpress bcl-2 and in HL60 and HeLa cell lines, expressing the endogenous bcl-2 protein. We demonstrated that this interaction is not involved in canonical bcl-2 functions. As expected, bcl-2 overexpression exerts anti-apoptotic effect reducing the percentage of apoptotic cells observed after treatment with CPT and DDP, but the downregulation of SLIRP, both at transcript and protein level, did not influence this response. The significant rise of ROS production upon H_2_O_2_ treatment was also dramatically reduced by bcl-2 overexpression, but not affected by SLIRP silencing.

To evaluate whether the bcl-2 binding to SLIRP was able to affect the expression of known SLIRP targets, the levels of seven transcripts, representative of mitochondrial mRNA and part of respiratory chain complexes I, III, IV and V were evaluated both in lung and breast models. In particular, we analyzed the expression of COX1, component of the respiratory chain that catalyzes the reduction of oxygen to water; ND1, ND3, ND5, ND6, core subunits of the mitochondrial membrane respiratory chain NADH dehydrogenase; ATPase 6/8, that produces ATP from ADP in the presence of a proton gradient across the membrane, which is generated by electron transport complexes of the respiratory chain and CYTB, component of the ubiquinol–cytochrome *c* reductase complex, which is a respiratory chain that generates an electrochemical potential coupled to ATP synthesis.

Both lung and breast cancer cells showed higher levels of COX1, ND1, ATPase 6/8 and CYTB after bcl-2 overexpression, but not a significant modulation of ND3, ND5 and ND6 transcripts. Moreover, although SLIRP silencing did not affect the levels of ND3, ND5 and ND6, it induced the downregulation of COX1, ND1 and ATPase 6/8 mRNA. These data, in agreement with previous results obtained in HeLa cell line,^20^ were observed regardless of bcl-2 expression, thus evidencing that the coexistence of bcl-2 overexpression and presence of endogenous SLIRP is necessary to explain the increased levels of mRNA observed in cells forced to express bcl-2. Regarding the effect of SLIRP silencing on CYTB transcript level, it strictly depends on the experimental model used. According to Chujo *et al*, CYTB was mildly affected by SLIRP in HeLa cells but it was significantly decreased upon SLIRP knockdown in MCH58 immortalized human fibroblasts^24^ and it was not decreased by SLIRP siRNA in our experimental condition. We also demonstrated an increased SLIRP protein, but not mRNA, level after bcl-2 overexpression. To evaluate a possible involvement of bcl-2 on SLIRP protein stabilization, which in turn may positively affect mitochondrial mRNA levels, a proteasome inhibitor demonstrated SLIRP accumulation in control, but not in bcl-2-overexpressing cells. This observation indicates that bcl-2 can render SLIRP less susceptible to ubiquitin modification, which targets proteins for proteasome degradation, thus promoting the stabilization of SLIRP protein and positively affecting the level of mitochondrial mRNA. To this regard, it is interesting to underline that among the identified proteins listed in Supplementary Tables 1, we also reported proteasome subunits (alpha type 1, 2, 4, 5) and 26 S proteasome regulatory subunits, supporting the idea of protein degradation machinery involvement in regulating bcl-2/SLIRP binding.

It is largely demonstrated that the structural organization of bcl-2 protein into functional domains is necessary to explain all known cellular functions. In particular, the role of BH4 domain in mediating the anti-apoptotic bcl-2 functions, as well as several non-canonical bcl-2 functions, through the binding with different regulating proteins has been extensively investigated. Here, we provided evidences that full-length bcl-2 protein is necessary to induce higher level of mitochondrial mRNA. In fact, overexpression of the deleted form of bcl-2 lacking the BH4 domain suppressed the positive effect on mRNA expression mediated by bcl-2, indicating that BH4 domain has a role in this novel bcl-2/SLIRP interaction. This consideration is also supported by the evidence of a reduced co-immunoprecipitation of bcl-2 and SLIRP observed in IM obtained from cells overexpressing the BH4 deleted form of bcl-2 and by the unvaried SLIRP protein level observed between H1299 control and bcl-2del cells.

In conclusion, we evidenced, for the first time, a novel mitochondrial associated function related to bcl-2. Mitochondria are key organelle of the cells, being involved not only in obtaining energy through the oxidative phosphorylation system, but also in other regulating signals through mitochondrial ROS, such as regulation of membrane potential and cell death, modulation of calcium signaling and steroid production.^31^ A unique aspect of mitochondria is the presence of own mitochondrial DNA, which encodes only a few crucial proteins. Consequently, the proper mitochondria functionality is guaranteed by a fine-tuned importation of proteins encoded by nuclear DNA. Several nuclear factors are known or are predicted to play a role in mitochondrial RNA biology,^32^ but among them bcl-2 has not been reported until now. In this view, our work represents the first evidence of bcl-2 involvement in regulating the level of mitochondrial transcripts through the interaction with SLIRP.

## Materials and Methods

### Cell culture, transfection and treatment

Lung H1299 and breast MDA-MB-231 human adenocarcinoma cell lines were cultured in RPMI (Euroclone, Pero, Italy) and Dulbecco's modified essential medium (Lonza, Verviers, Belgium) respectively, in the presence of geneticin (Sigma-Aldrich, St Louis, MO, USA) and 10% fetal bovine serum (HyClone, Thermoscientific, South Logan, UT, USA). Human epithelial cervix adenocarcinoma HeLa and human acute promyelocytic leukemia HL60 cell lines were maintained in 10% fetal bovine serum and Dulbecco's modified essential medium or RPMI, respectively.

H1299 and MDA-MB-231 stable clones overexpressing the bcl-2 wild-type protein fused to FLAG epitope were obtained transfecting parental cell lines with plasmids kindly provided by Professor Giulio Taglialatela, University of Texas Medical Branch at Galveston, Texas, USA. The pCMV-Tag-2B plasmid containing the human bcl-2 codifying sequence cloned in frame with the FLAG peptide sequence at the N-terminal was used to overexpress bcl-2. The empty vector pCMV-Tag-2B was used as control plasmid. After transfection by JetPRime reagent (Polyplus transfection, Illkirch, France) according to the manufacturer's protocol, H1299 and MDA-MB-231 cells were cultured in the presence of 800 *μ*g/ml and 1000 *μ*g/ml geneticin, respectively. Same procedures of transfection and selection of clones were used to obtain stable H1299 clone overexpressing the mutant form of bcl-2 protein, lacking the 1–36 aa sequence (corresponding to the BH4 domain), fused to FLAG sequence at N-terminal. To this purpose, the sequence encoding the human bcl-2 lacking the BH4 domain was excised from the pIDTSmart plasmid purchased by IDT (Integrated DNA Technologies Munich, Germany) using *Eco*RI/*Xho*I restriction enzymes and the obtained fragment was inserted in frame with FLAG peptide sequence in the pCMV-Tag-2B vector using the corresponding *Eco*RI/*Xho*I restriction sites.

Pooled siRNA oligonucleotides against SLIRP or scramble target sequences were purchased from DharmaconRNA Technologies (siGENOME SMARTpool, Lafayette, CO, USA). For siRNA transfection, cells were seeded and after 24 h transfected with 50 nM pooled oligonucleotides mixture by using Lipofectamine2000 (Invitrogen, Grand Island, NY, USA) following the manufacturer's protocol. After 6 h, medium was changed. Gene silencing efficacy by siRNA after 4 days of transfection was assessed by Western blot or quantitative real-time PCR (qRT-PCR) analyses.

For treatment with the proteasome inhibitor MG132 (Sigma-Aldrich), cells were seeded and after 24 h medium was replaced with medium containing 10 *μ*M MG132. Cells were collected after 6 h treatment.

### Antibodies and reagents

Dithiothreitol, iodoacetamide, paraformaldehyde (PFA), CPT, proteases and phosphatases inhibitors cocktails were from Sigma-Aldrich. CHAPS was from Applichem (Darmstadt, Germany). DDP was from Pfizer (New York, NY, USA). Rabbit polyclonal antibody against SLIRP (Abcam Cambridge, UK), mouse monoclonal antibody against FLAG peptide (Sigma-Aldrich), mouse monoclonal against *β*-actin (Sigma-Aldrich), mouse monoclonal antibody against bcl-2 (Santa Cruz Biotechnology, Inc, Dallas, TX, USA) and rabbit polyclonal antibody against bcl-2 (Santa Cruz Biotechnology) were used. Lipofectamine2000 and BCA reagents were purchased from Invitrogen. Enhanced chemiluminescence (ECL) reagents from Pierce Biotechnology (Rockford, IL, USA), Protein A and Protein G agarose beads from Amersham Biosciences Europe (Milan, Italy), sequencing-grade modified trypsin from Promega (Madison, WI, USA) were used. Acetonitrile (ACN), formic acid (FA) and water for mass spectrometry were from Sigma. Water used in this study was deionized using a Milli-Q purification system (Millipore, Billerica, MA, USA).

### Immunoprecipitation for mass spectrometry

Cells were washed with phosphate-buffered saline (PBS) and incubated in 1.0% (w/v) PFA in PBS for 10 min at 37 °C. To stop the cross-linking reaction, 1.25 M glycine was added to a final concentration of 125 mM for 5 min at room temperature (RT).^8^ Cells were washed three times with PBS, harvested and lysed in 40 mM HEPES, pH 7.4, 150 mM NaCl, 0.3% CHAPS, in the presence of proteases and phosphatases inhibitors. Cellular debris was removed by centrifugation for 15 min at 10 000 × *g*, 4 °C. Supernatants were collected and protein concentrations were measured in duplicate using a BCA protein assay kit.

Protein extracts (500 *μ*g) for each control and test sample were pre-cleared for 1 h at 4 °C by the addition of 20 *μ*l of Protein A/G agarose beads. The pre-cleared lysates were centrifuged and transferred to a new microfuge tube. 2 *μ*g of FLAG-specific antibody and fresh Protein A/G agarose beads were added and allowed to complex overnight at 4 °C. Samples were washed and after complete removal of the supernatant with a microliter syringe, immunoprecipitated proteins were eluted with 20 *μ*l of Laemnli sample buffer, without reducing agent and boiled for 20 min at 95 °C. Two eluted samples (500 *μ*g × 2 of starting materials) for each condition were pooled and separated by 4–20% precast polyacrylamide gel (Bio-Rad, Hercules, CA, USA). After Coomassie blue staining each individual lane was fractionated into 12 slices of equal dimension for analysis. In addition, a small fraction of the immunoprecipitate (6 *μ*l) after 4–20% SDS-PAGE was transferred and immunoblotted to test FLAG-bcl-2 precipitation.

### Mass spectrometry analysis

Gel slices were washed in 100 mM ammonium bicarbonate (pH 8) and 50% ACN until complete destaining and, after reduction by dithiothreitol and alkylation by iodoacetamide, were digested with sequencing-grade trypsin at 37 °C. After incubation overnight, peptides were extracted sequentially three times with 50% ACN and 0.1% FA in water. The original supernatant and those obtained from sequential extractions were combined and completely dried down. Dried peptide fractions were resuspended in 20 *μ*l of 5% (v/v) ACN in 0.1% (v/v) FA. Each sample was separated by a Proxeon Easy-nLC II (Thermo Scientific, Waltham, MA, USA) chromatographic system equipped with an EASY-Column C18, 5 *μ*m, 100 *μ*m × 2 cm precolumn (Thermo Scientific) and an Acclaim PepMap100 C18, 5 *μ*m, 75  *μ*m × 25 cm (Dionex, Thermo Scientific) nanoscale column. Peptides were captured on precolumn in mobile phase A (0.1% FA) and washed for 8 min, then separated with a gradient of 10–30% mobile phase B (0.1% AF in ACN) over 120 min at flow rate of 300 nl/min. The chromatographic system was interfaced to an amaZon ion trap (Bruker-Daltonics) operating in AutoMSn (with *n*=2) in Enhanced Resolution (maximum speed=8100 *m*/*z* per s) for MS mode and Ultrascan Mode for MS/MS. The mass-acquisition mode involved scans in the range from 300 to 1500 *m*/*z* followed by three tandem scans in the ion trap. The three most intense peaks (over an intensity threshold of 25 000 a.u.) from each scan were selected in the ion trap for further fragmentation. Proteomic data were analyzed by Compass DataAnalysis 4.0 software (Bruker-Daltonics, Bremen, Germany) for mass spectra deconvolution. Protein identification was achieved by ProteinScape 2.1 Bioinformatics Platform (Bruker-Daltonics) running database search against SwissProt 2013_02 restricted to Homo sapiens taxonomy (20 248 sequences), and using Mascot algorithm (version 2.4), fixing the following parameter: maximal error tolerances of 0.3 Da for precursor, 0.6 Da for fragments, carbamidomethylation of cysteines as fixed modification, oxidation of methionines as variable modification, one allowed missing cleavage on tryptic peptides, decoy option active, significant threshold 0.05. Protein List Compilation was generated by Protein Extractor and Assessment was achieved by applying these parameters: (i) accept proteins if false discovery rate (%)<1.0; (ii) accept peptides if: Mascot>32; (iii) for each peptide, accept: top hit compound only. In case of MS/MS spectra matching peptides from more than one identified protein, accept: highest scoring peptide (rank 1 peptide).

### Bioinformatics analysis

Modulated proteins identified by proteomic analysis were further analyzed by the PANTHER Classification System (http://www.pantherdb.org) and QIAGEN's IPA (Qiagen, Redwood City, CA, USA). Using PANTHER resource, it is possible to categorize genes by their molecular functions or biological processes on the basis of published papers and by evolutionary relationships to predict function when experimental evidence is missing. IPA highlights protein networks or pathways starting from a continuous updated database of known protein–protein interactions based on direct (physical) and indirect (functional) associations. The algorithm gives back a probability score for each possible network. Scores of 10 or higher (negative log of the *P-value*) have a high confidence of not being generated by random chance alone.^23, 33, 34^

### Western blot analysis

Cells were lysed by sonication in 10 mM Tris-HCl, pH 7.4, 2% SDS, in the presence of proteases inhibitors. Protein concentrations were measured using a BCA protein assay kit. 40 *μ*g of total protein extracts were separated by SDS-PAGE using 4–20% precast polyacrylamide gel. Gels were transferred using the Trans-Blot Turbo Transfer System (Bio-Rad) onto nitrocellulose membranes and blocked in 5% non-fat dry milk in 1 × tris-buffered saline (TBS) containing 0.05% Tween-20 (T-TBS) and then incubated over night at 4 °C in primary antibody diluted in 1% non-fat dry milk in T-TBS. Membranes were washed in 1 × T-TBS and incubated for 1 h in the appropriate secondary antibody in 1% non-fat dry milk in T-TBS. The blots were washed three times with 1 × T-TBS, detected using the Pierce ECL Plus kit (Pierce, Rockford, IL, USA).

### Immunocytochemistry

Cells were washed in PBS and fixed with 4% PFA in complete medium for 15 min. To stain mitochondria, MitoTracker (Invitrogen) was applied to cells, before fixation, 15 min at 100 nM. After permeabilization with 0.2% Triton X-100 in PBS for 5 min, cells were blocked in 10% bovine serum albumin in PBS and incubated for 2 h at RT with primary antibodies. We used the antibodies directed against FLAG tag (1 : 500) and SLIRP (1 : 200) diluted in 3% bovine serum albumin in PBS. Cells were then washed in PBS and incubated 1 h at RT with anti-mouse (TRITC, Jackson ImmunoResearch, West Grove, PA, USA) and anti-rabbit (FITC, Jackson ImmunoResearch) secondary antibodies. Nuclei were stained with 1 mg/ml DAPI. Images were scanned under a × 63 and × 100 oil immersion objectives and, to avoid bleed-through effects, each fluorescent signal was scanned independently by using a LeicaDMIRE2 microscope equipped with a Leica DFC350FX camera, elaborated by a Leica FW4000 deconvolution software (Leica, Solms, Germany) and processed using Adobe PhotoShop CS5 software (Adobe System Incorporated, San Jose, CA, USA) to adjust image brightness and contrast.

### Flow cytometric analysis

Flow cytometric analysis (BD Accuri™ C6, BD Biosciences, Franklin Lakes, NJ, USA) was performed to evaluate apoptosis by Annexin V-FITC/PI staining^35^ and ROS level.^36^ Cells were transfected by using siRNA against SLIRP or scramble siRNA and after 24 h medium was replaced with medium containing 0.5 *μ*M CPT or 20 *μ*M DDP. After 24 h treatment with DDP or 48 h treatment with CPT, cells were collected and stained using the Annexin V-FITC apoptosis detection kit by BD Biosciences according to the instruction. To evaluate ROS levels, after 4 days of transfection with siRNA against SLIRP or scramble siRNA, medium was changed and replaced with medium containing 5 mM H_2_O_2_. After 30 min treatment, cells were removed from growth media by centrifugation, resuspended in pre-warmed PBS containing 1 *μ*M 5–6-Chloromethyl-20,70-dichlorofluorescindiacetate, DCFDA (Molecular Probes, Invitrogen, Carlsbad, CA, USA) and incubated at 37 °C for 30 min in the dark. After removed, the loading buffer cells were returned into pre-warmed growth media to allow short recovery prior flow cytometry analysis.

### qRT-PCR

Total RNA was extracted using a Qiagen RNeasy Mini kit (Qiagen) according to the manufacturer's instructions. Reverse transcription was performed using RevertAid Reverse Transcriptase (Thermo Scientific). Reaction conditions were: 50 °C for 60 min, 85 °C for 5 min, 4 °C until stopped.

qRT-PCR was performed using a Gene-Amp 5700 sequence detection system (Applied Biosystems, Foster City, CA, USA), using the SYBR green dye detection method. The mRNA levels were normalized using *β*-actin. Primers used to analyze each gene are listed in Supplementary Table 9. All qPCR mixtures contained 500 ng of cDNA template, 300 nM of each primer, 15 *μ*l of Master mix (Applied Biosystem) in a final volume of 30 *μ*l. Cycling conditions were 50 °C for 2 min, 95 °C for 10 min followed by 46 cycles at 95 °C for 15 s and 60 °C for 1 min, with final dissociation step at 95 °C for 15 s, 60 °C for 1 min and 95 °C for 15 s.

The fold change in gene expression levels, expressed in unitless values, was evaluated using the 2^–ΔΔCt^ method.^37^

### Statistical analysis

Experiments were replicated at least three times, and the data were expressed as mean±standard error of the mean (S.E.M.). Differences between conditions were analyzed with *t-*test and were considered to be statistically significant for *P*<0.05.

## Abbreviations

BH: bcl-2 homology
ROS: reactive oxygen species
PBS: phosphate-buffered saline
TBS: tris-buffered saline
PFA: paraformaldehyde
ACN: acetonitrile
FA: formic acid
nLC-MS/MS: nanoLiquid chromatography tandem mass spectrometry
mRNA: messenger RNA
qRT-PCR: quantitative real-time PCR
S.E.M.: standard error of the mean
PI: propidium iodide
CPT: camptothecin
DDP: cisplatin
SLIRP: SRA stem–loop interacting RNA-binding protein
VDAC1: voltage-dependent anion channel 1
COX5A: cytochrome *c* oxidase subunits Va
LRPPRC: leucine-rich pentatricopeptide repeat-containing
CYTB: cytochrome *b*
COX1: cytochrome *c* oxidase
ND1: NADH-ubiquinone oxidoreductase chain 1
ATPase 6/8: ATP synthase
ND3: NADH-ubiquinone oxidoreductase chain 3
ND5: NADH-ubiquinone oxidoreductase chain 5
ND6: NADH-ubiquinone oxidoreductase chain 6
RT: room temperature
IM: immunocomplex
IPA: Ingenuity Pathway Analysis software
