# Supplementary figures and images for: Affinity purification-mass spectrometry analysis of bcl-2 interactome identified SLIRP as a novel interacting protein
Source: Cell Death Dis. 2016 Feb 11;7(2):e2090–. doi: 10.1038/cddis.2015.357 (PMC4849145; doi:10.1038/cddis.2015.357)

100X

nuclei

bcl-2

SLIRP

merge

MDA MB 231  
Flag bcl-2

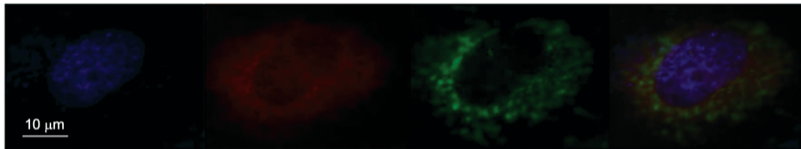

100X

nuclei

bcl-2

SLIRP

merge

H1299  
Flag bcl-2

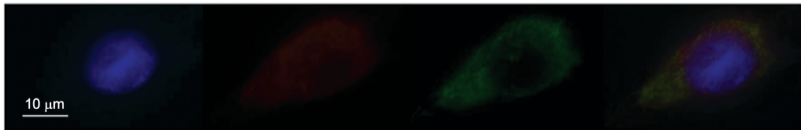

Supplement: Supplementary Figure 1 [file cddis2015357x1.pdf]
