# Supplementary material for: Affinity purification-mass spectrometry analysis of bcl-2 interactome identified SLIRP as a novel interacting protein
Source: Cell Death Dis. 2016 Feb 11;7(2):e2090–. doi: 10.1038/cddis.2015.357 (PMC4849145; doi:10.1038/cddis.2015.357)
Supplement: Supplementary Table 1 [file cddis2015357x2.docx]

**Supplementary Table 1. List of identified protein.** For each identified proteins the SwissProt accession number, protein name, theoretical mass and pI, the sequence coverage and the matched peptides are reported.

**Theoretical Mascot % Seq. Matched**

**Accession Protein Name M_r_(kDa)/pI Score Coverage Peptides**

1433E_HUMAN 14-3-3 protein epsilon 29.2/4.5 566.4 43.5 9

1433F_HUMAN 14-3-3 protein eta 28.2/4.6 250.7 21.1 4

1433G_HUMAN 14-3-3 protein gamma 28.3/4.7 393.7 30.4 7

1B57_HUMAN HLA class I histocompatibility antigen, B-57 alpha chain 40.2/5.9 58.4 9.1 1

AAKG2_HUMAN 5'-AMP-activated protein kinase subunit gamma-2 63.0/9.8 52.8 4.0 2

AATM_HUMAN Aspartate aminotransferase, mitochondrial 47.5/9.8 361.5 19.5 5

ADT2_HUMAN ADP/ATP translocase 2 32.8/10.2 564.8 37.2 7

ADT3_HUMAN ADP/ATP translocase 3 32.8/10.3 516.1 37.2 7

AIMP1_HUMAN Aminoacyl tRNA synthase complex-interacting 34.3/9.4 52.0 3.8 1

multifunctional protein 1

AMOT_HUMAN Angiomotin 118.0/7.5 76.6 2.0 3

ANX11_HUMAN Annexin A11 54.4/8.5 81.6 7.3 1

ANXA2_HUMAN Annexin A2 38.6/8.5 1081.8 63.7 13

ANXA4_HUMAN Annexin A4 35.9/5.8 37.0 5.0 1

ARF4_HUMAN ADP-ribosylation factor 4 20.5/7.5 231.7 32.8 5

ARF5_HUMAN ADP-ribosylation factor 5 20.5/6.4 214.1 28.9 4

ARP3_HUMAN Actin-related protein 3 47.3/5.5 138.6 9.8 1

ARPC4_HUMAN Actin-related protein 2/3 complex subunit 4 19.7/9.4 153.4 17.9 2

AT1B3_HUMAN Sodium/potassium-transporting ATPase subunit beta-3 31.5/9.3 42.6 5.0 1

ATS10_HUMAN A disintegrin and metalloproteinase with 120.8/9.7 48.8 1.6 2

thrombospondin motifs 10

BAG2_HUMAN BAG family molecular chaperone regulator 2 23.8/6.3 69.5 9.5 1

BAP31_HUMAN B-cell receptor-associated protein 31 28.0/9.1 183.6 19.9 2

BCL2_HUMAN Apoptosis regulator Bcl-2 26.2/6.9 164.0 14.2 2

CALL5_HUMAN Calmodulin-like protein 5 15.9/4.1 76.9 21.2 1

CALM_HUMAN Calmodulin 16.8/3.9 90.9 29.5 3

CAP1_HUMAN Adenylyl cyclase-associated protein 1 51.9/9.1 157.5 16.8 1

CAPZB_HUMAN F-actin-capping protein subunit beta 31.3/5.2 167.7 15.2 3

CATA_HUMAN Catalase 59.7/7.0 32.8 2.5 1

CE290_HUMAN Centrosomal protein of 290 kDa 290.2/5.7 146.7 2.9 7

CENPF_HUMAN Centromere protein F 367.5/4.9 185.7 3.4 1

CHCH3_HUMAN Coiled-coil-helix-coiled-coil-helix domain-containing protein 3, 26.1/9.3 110.4 11.5 2

mitochondrial

CHRD1_HUMAN Cysteine and histidine-rich domain-containing protein 1 37.5/9.2 33.6 4.5 1

CISY_HUMAN Citrate synthase, mitochondrial 51.7/9.1 97.9 10.3 4

CLCA_HUMAN Clathrin light chain A 27.1/4.3 188.9 13.7 3

CLCB_HUMAN Clathrin light chain B 25.2/4.4 109.6 11.8 2

CLK3_HUMAN Dual specificity protein kinase CLK3 73.5/10.7 35.6 3.1 1

CNN3_HUMAN Calponin-3 36.45.6 210.9 20.4 2

COF1_HUMAN Cofilin-1 18.5/9.1 175.6 30.1 3

COTL1_HUMAN Coactosin-like protein 15.9/5.4 64.8 10.6 1

COX2_HUMAN Cytochrome c oxidase subunit 2 25.5/4.5 112.7 11.9 2

COX5A_HUMAN Cytochrome c oxidase subunit 5A, mitochondrial 16.8/6.4 68.9 20.7 2

COX6C_HUMAN Cytochrome c oxidase subunit 6C 8.8/10.8 39.1 10.7 1

CUX2_HUMAN Homeobox protein cut-like 2 161.6/5.3 91.9 2.6 1

CYBP_HUMAN Calcyclin-binding protein 26.2/9.0 183.3 38.2 2

DBLOH_HUMAN Diablo homolog, mitochondrial 27.1/5.6 68.2 4.2 1

DDX6_HUMAN Probable ATP-dependent RNA helicase DDX6 54.4/9.6 69.0 6.4 3

DEST_HUMAN Destrin 18.5/9.2 35.4 6.7 1

DHSA_HUMAN Succinate dehydrogenase [ubiquinone] flavoprotein subunit, 72.6/7.3 50.3 2.1 1

mitochondrial

DHSB_HUMAN Succinate dehydrogenase [ubiquinone] iron-sulfur subunit, 31.6/9.9 70.2 11.4 3

mitochondrial

DOP2_HUMAN Protein dopey-2 258.1/5.9 111.5 1.7 1

DPM1_HUMAN Dolichol-phosphate mannosyltransferase 29.6/10.0 40.7 4.2 1

DRG1_HUMAN Developmentally-regulated GTP-binding protein 1 40.5/9.6 161.5 13.4 2

DX39B_HUMAN Spliceosome RNA helicase DDX39B 49.0/5.3 117.3 8.6 1

DYH12_HUMAN Dynein heavy chain 12, axonemal 356.7/5.8 83.4 1.9 4

ECI1_HUMAN Enoyl-CoA delta isomerase 1, mitochondrial 32.8/9.7 105.2 9.3 2

EIF2A_HUMAN Eukaryotic translation initiation factor 2A 64.9/9.6 87.7 5.0 2

EIF3E_HUMAN Eukaryotic translation initiation factor 3 subunit E 52.2/5.7 63.5 7.2 3

EIF3F_HUMAN Eukaryotic translation initiation factor 3 subunit F 37.5/5.1 89.1 10.1 1

EIF3M_HUMAN Eukaryotic translation initiation factor 3 subunit M 42.5/5.3 71.1 5.1 1

ERF1_HUMAN Eukaryotic peptide chain release factor subunit 1 49.0/5.4 132.8 9.4 1

ERP29_HUMAN Endoplasmic reticulum resident protein 29 29.0/7.5 84.3 8.4 2

FA49B_HUMAN Protein FAM49B 36.7/5.7 102.5 12.7 4

FA7_HUMAN Coagulation factor VII 51.6/7.2 74.3 5.8 3

FAM3C_HUMAN Protein FAM3C 24.7/9.4 59.1 12.3 2

FANCJ_HUMAN Fanconi anemia group J protein 140.8/6.5 49.5 1.9 2

FHL1_HUMAN Four and a half LIM domains protein 1 36.2/10.5 80.6 5.6 1

FSIP2_HUMAN Fibrous sheath-interacting protein 2 780.1/6.3 69.5 0.3 3

FYV1_HUMAN 1-phosphatidylinositol-3-phosphate 5-kinase 237.0/6.2 71.8 0.9 2

G6PI_HUMAN Glucose-6-phosphate isomerase 63.1/9.1 281.9 18.3 3

GALT6_HUMAN Polypeptide N-acetylgalactosaminyltransferase 6 71.1/9.4 51.6 2.3 2

GDIB_HUMAN Rab GDP dissociation inhibitor beta 50.6/6.1 87.5 9.0 1

GFPT1_HUMAN Glucosamine--fructose-6-phosphate aminotransferase 78.8/6.7 78.9 3.4 1

[isomerizing] 1

GLYM_HUMAN Serine hydroxymethyltransferase, mitochondrial 56.0/9.5 83.3 6.7 1

GRSF1_HUMAN G-rich sequence factor 1 53.1/5.8 90.4 8.1 1

HAUS5_HUMAN HAUS augmin-like complex subunit 5 71.6/9.9 39.4 1.9 1

HNRPQ_HUMAN Heterogeneous nuclear ribonucleoprotein Q 69.6/9.1 79.9 5.5 3

HXA6_HUMAN Homeobox protein Hox-A6 26.3/9.8 34.2 6.0 1

IDH3A_HUMAN Isocitrate dehydrogenase [NAD] subunit alpha, mitochondrial 39.6/6.5 106.8 12.3 1

IF2A_HUMAN Eukaryotic translation initiation factor 2 subunit 1 36.1/4.9 267.8 22.9 2

IF2G_HUMAN Eukaryotic translation initiation factor 2 subunit 3 51.1/9.5 139.7 8.9 3

IF4A3_HUMAN Eukaryotic initiation factor 4A-III 46.8/6.3 93.5 8.3 4

IF4E_HUMAN Eukaryotic translation initiation factor 4E 25.1/5.8 41.7 5.1 1

IF4H_HUMAN Eukaryotic translation initiation factor 4H 27.4/7.5 144.3 14.1 2

IF5A1_HUMAN Eukaryotic translation initiation factor 5A-1 16.8/4.9 115.5 12.3 1

IF6_HUMAN Eukaryotic translation initiation factor 6 26.6/4.4 93.8 21.2 2

IFT57_HUMAN Intraflagellar transport protein 57 homolog 49.1/4.8 56.8 6.3 2

ILF2_HUMAN Interleukin enhancer-binding factor 2 43.0/5.0 98.9 9.7 1

IMA1_HUMAN Importin subunit alpha-1 57.8/5.1 194.6 9.8 3

JPH3_HUMAN Junctophilin-3 81.4/10.0 35.9 1.6 1

KCRB_HUMAN Creatine kinase B-type 42.6/5.2 82.9 8.9 1

KIF4B_HUMAN Chromosome-associated kinesin KIF4B 139.9/5.8 54.3 1.1 2

LDHB_HUMAN L-lactate dehydrogenase B chain 36.6/5.7 616.7 30.8 8

LIS1_HUMAN Platelet-activating factor acetylhydrolase IB subunit alpha 46.6/7.2 58.6 5.6 1

LRC8E_HUMAN Leucine-rich repeat-containing protein 8E 90.2/6.5 62.2 2.0 1

LSM12_HUMAN Protein LSM12 homolog 21.7/8.9 35.7 6.7 1

LTBP2_HUMAN Latent-transforming growth factor beta-binding protein 2 194.9/4.9 64.6 1.9 3

MARCS_HUMAN Myristoylated alanine-rich C-kinase substrate 31.5/4.3 110.5 14.5 2

MGST3_HUMAN Microsomal glutathione S-transferase 3 16.5/10.1 35.0 8.6 1

MIF_HUMAN Macrophage migration inhibitory factor 12.5/9.1 87.6 17.4 1

MLX_HUMAN Max-like protein X 33.3/9.1 64.5 7.4 3

MOB1A_HUMAN MOB kinase activator 1A 25.1/6.5 85.8 11.1 1

MOES_HUMAN Moesin 67.8/6.0 135.9 6.2 2

MYH11_HUMAN Myosin-11 227.2/5.3 102.3 1.6 4

MYL6_HUMAN Myosin light polypeptide 6 16.9/4.4 273.2 47.7 4

NALD2_HUMAN N-acetylated-alpha-linked acidic dipeptidase 2 83.5/9.0 49.2 1.8 1

NCEH1_HUMAN Neutral cholesterol ester hydrolase 1 45.8/6.9 60.3 6.9 2

NDUS3_HUMAN NADH dehydrogenase [ubiquinone] iron-sulfur protein 3, 30.2/7.8 74.9 9.8 2

mitochondrial

NDUS8_HUMAN NADH dehydrogenase [ubiquinone] iron-sulfur protein 8, 23.7/6.0 77.0 9.5 1

mitochondrial

NFX1_HUMAN Transcriptional repressor NF-X1 124.3/10.2 34.1 0.7 1

NONO_HUMAN Non-POU domain-containing octamer-binding protein 54.2/9.6 351.1 23.4 2

NP1L1_HUMAN Nucleosome assembly protein 1-like 1 45.3/4.2 187.1 12.5 3

NSMA_HUMAN Sphingomyelin phosphodiesterase 2 47.6/6.5 65.7 7.3 3

OCAD1_HUMAN OCIA domain-containing protein 1 27.6/7.8 67.7 13.5 3

OLA1_HUMAN Obg-like ATPase 1 44.7/8.6 76.3 3.8 1

OST48_HUMAN Dolichyl-diphosphooligosaccharide-protein glycosyltransferase 50.8/6.1 61.9 4.8 2

48 kDa subunit

PDCD5_HUMAN Programmed cell death protein 5 14.3/5.7 135.0 19.2 2

PDCD6_HUMAN Programmed cell death protein 6 21.9/5.0 71.3 12.0 1

PGK1_HUMAN Phosphoglycerate kinase 1 44.6/9.2 810.9 50.1 10

PI4KA_HUMAN Phosphatidylinositol 4-kinase alpha 231.2/6.4 83.9 2.0 4

PPIA_HUMAN Peptidyl-prolyl cis-trans isomerase A 37.5/5.9 204.5 20.6 3

PRDX1_HUMAN Peroxiredoxin-1 22.1/9.2 467.0 45.7 8

PRDX3_HUMAN Thioredoxin-dependent peroxide reductase, mitochondrial 27.7/8.9 134.6 10.2 2

PRS10_HUMAN 26S protease regulatory subunit S10B 44.1/7.8 34.6 4.4 1

PRS4_HUMAN 26S protease regulatory subunit 4 49.2/5.8 63.0 2.7 1

PRS7_HUMAN 26S protease regulatory subunit 7 48.6/5.6 80.8 6.7 3

PSA1_HUMAN Proteasome subunit alpha type-1 29.5/6.2 88.4 12.9 1

PSA2_HUMAN Proteasome subunit alpha type-2 25.9/7.7 81.5 16.7 1

PSA4_HUMAN Proteasome subunit alpha type-4 29.5/8.7 172.1 16.5 1

PSA5_HUMAN Proteasome subunit alpha type-5 26.4/4.6 285.0 29.5 4

PSB5_HUMAN Proteasome subunit beta type-5 28.5/6.5 40.0 3.8 1

PSMD3_HUMAN 26S proteasome non-ATPase regulatory subunit 3 60.9/9.0 103.6 8.1 1

PSME1_HUMAN Proteasome activator complex subunit 1 28.7/5.7 113.0 12.4 1

QCR7_HUMAN Cytochrome b-c1 complex subunit 7 13.5/9.2 41.8 11.7 1

RA1L2_HUMAN Heterogeneous nuclear ribonucleoprotein A1-like 2 34.2/9.6 38.5 3.1 1

RAB10_HUMAN Ras-related protein Rab-10 22.5/9.4 276.7 25.5 4

RAB18_HUMAN Ras-related protein Rab-18 23.0/5.0 134.9 24.3 2

RAB21_HUMAN Ras-related protein Rab-21 24.3/9.2 84.4 20.4 1

RAE1_HUMAN Rab proteins geranylgeranyltransferase component A 1 73.4/4.5 36.4 1.4 1

RANG_HUMAN Ran-specific GTPase-activating protein 23.3/5.1 120.7 14.9 2

RAP1B_HUMAN Ras-related protein Rap-1b 20.8/5.5 237.8 34.8 3

RAP2B_HUMAN Ras-related protein Rap-2b 20.5/4.6 70.2 6.6 1

RASN_HUMAN GTPase NRas 21.2/4.9 51.8 6.3 1

RB11A_HUMAN Ras-related protein Rab-11A 24.4/6.1 74.2 10.6 2

RB11B_HUMAN Ras-related protein Rab-11B 24.5/5.6 381.5 42.2 5

RBBP4_HUMAN Histone-binding protein RBBP4 47.6/4.6 63.7 3.5 2

RBMX_HUMAN RNA-binding motif protein, X chromosome 42.3/10.0 211.8 17.1 3

RL10_HUMAN 60S ribosomal protein L10 24.6/11.0 287.2 25.2 3

RL13_HUMAN 60S ribosomal protein L13 24.2/12.2 338.4 29.4 6

RL21_HUMAN 60S ribosomal protein L21 18.6/11.0 118.5 31.9 1

RL36_HUMAN 60S ribosomal protein L36 12.2/12.2 119.5 28.6 2

RL38_HUMAN 60S ribosomal protein L38 8.2/10.7 39.3 14.3 1

RM01_HUMAN 39S ribosomal protein L1, mitochondrial 36.9/9.5 51.7 7.4 1

RMD1_HUMAN Regulator of microtubule dynamics protein 1 35.8/9.2 40.9 2.9 1

ROA0_HUMAN Heterogeneous nuclear ribonucleoprotein A0 30.8/9.8 64.4 8.9 1

ROA3_HUMAN Heterogeneous nuclear ribonucleoprotein A3 39.6/9.6 164.0 15.9 3

ROAA_HUMAN Heterogeneous nuclear ribonucleoprotein A/B 36.2/8.8 120.4 9.3 2

RPC7_HUMAN DNA-directed RNA polymerase III subunit RPC7 25.9/4.4 68.3 6.7 3

RS27A_HUMAN Ubiquitin-40S ribosomal protein S27a 18.0/10.4 233.4 46.2 2

RS29_HUMAN 40S ribosomal protein S29 6.7/11.8 50.8 14.3 1

RT22_HUMAN 28S ribosomal protein S22, mitochondrial 41.3/8.7 104.2 10.6 1

RTN3_HUMAN Reticulon-3 112.5/4.7 97.8 3.8 1

RU2A_HUMAN U2 small nuclear ribonucleoprotein A' 28.4/9.4 141.7 14.5 2

S11IP_HUMAN Serine/threonine-protein kinase 11-interacting protein 121.3/5.2 58.9 1.0 2

SBDS_HUMAN Ribosome maturation protein SBDS 28.7/9.7 58.2 6.8 1

SEP11_HUMAN Septin-11 49.4/6.4 72.8 6.5 1

SEPT2_HUMAN Septin-2 41.5/6.1 279.8 19.4 3

SLIRP_HUMAN SRA stem-loop-interacting RNA-binding protein, mitochondrial 12.3/11.0 44.0 9.2 1

SMAD6_HUMAN Mothers against decapentaplegic homolog 6 53.5/9.6 36.5 1.8 1

SMD2_HUMAN Small nuclear ribonucleoprotein Sm D2 13.5/10.6 83.0 16.9 2

SMD3_HUMAN Small nuclear ribonucleoprotein Sm D3 13.9/11.1 98.7 23.0 1

SNP23_HUMAN Synaptosomal-associated protein 23 23.3/4.7 56.0 13.7 1

SNRPA_HUMAN U1 small nuclear ribonucleoprotein A 31.3/10.3 57.8 3.5 1

SNX14_HUMAN Sorting nexin-14 110.1/6.3 65.4 1.5 1

SPTN1_HUMAN Spectrin alpha chain, non-erythrocytic 1 284.4/5.1 58.4 0.5 2

SQRD_HUMAN Sulfide:quinone oxidoreductase, mitochondrial 49.9/9.7 37.3 1.6 1

SRSF1_HUMAN Serine/arginine-rich splicing factor 1 27.7/10.8 120.6 13.7 2

SRSF2_HUMAN Serine/arginine-rich splicing factor 2 25.5/12.4 32.8 3.6 1

SSRA_HUMAN Translocon-associated protein subunit alpha 32.2/4.2 147.6 11.9 3

ST134_HUMAN Putative protein FAM10A4 27.4/4.8 172.8 15.4 3

STRAP_HUMAN Serine-threonine kinase receptor-associated protein 38.4/4.8 157.8 15.4 2

SUMO4_HUMAN Small ubiquitin-related modifier 4 10.7/7.5 58.0 12.6 1

SYK_HUMAN Lysyl-tRNA synthetase 68.0/5.9 41.1 1.8 1

TCP4_HUMAN Activated RNA polymerase II transcriptional coactivator p15 14.4/10.1 102.5 20.5 1

TCPE_HUMAN T-complex protein 1 subunit epsilon 59.6/5.3 404.2 18.5 5

TCPG_HUMAN T-complex protein 1 subunit gamma 60.5/6.1 676.7 31.2 8

TCPQ_HUMAN T-complex protein 1 subunit theta 59.6/5.3 1408.0 43.2 21

TFAM_HUMAN Transcription factor A, mitochondrial 29.1/10.3 115.6 13.8 1

TGFB3_HUMAN Transforming growth factor beta-3 47.3/9.3 32.1 2.7 1

THIC_HUMAN Acetyl-CoA acetyltransferase, cytosolic 41.3/6.5 63.1 8.1 1

THIL_HUMAN Acetyl-CoA acetyltransferase, mitochondrial 45.2/9.6 153.1 10.1 2

THIM_HUMAN 3-ketoacyl-CoA thiolase, mitochondrial 41.9/9.3 43.6 3.3 1

THIO_HUMAN Thioredoxin 11.7/4.7 71.1 12.4 1

TIM50_HUMAN Mitochondrial import inner membrane translocase subunit TIM50 39.6/9.3 321.5 24.6 6

TMM33_HUMAN Transmembrane protein 33 28.0/10.6 156.0 11.7 2

TMX1_HUMAN Thioredoxin-related transmembrane protein 1 31.8/4.8 67.7 14.3 3

TOM40_HUMAN Mitochondrial import receptor subunit TOM40 homolog 37.9/7.0 249.4 15.8 5

TPIS_HUMAN Triosephosphate isomerase 30.8/5.6 329.7 29.0 4

TR112_HUMAN TRM112-like protein 14.2/5.1 73.9 28.8 3

TRM44_HUMAN Probable tRNA (uracil-O(2)-)-methyltransferase 84.6/7.2 65.0 5.0 3

TYSY_HUMAN Thymidylate synthase 35.7/6.6 56.0 7.0 1

UAP1_HUMAN UDP-N-acetylhexosamine pyrophosphorylase 58.7/5.9 92.4 5.7 1

UB2V2_HUMAN Ubiquitin-conjugating enzyme E2 variant 2 16.4/9.0 36.8 6.9 1

UCRI_HUMAN Cytochrome b-c1 complex subunit Rieske, mitochondrial 29.6/9.4 143.8 18.6 2

UTRO_HUMAN Utrophin 394.2/5.1 154.2 2.3 7

VAMP3_HUMAN Vesicle-associated membrane protein 3 11.3/9.7 41.7 17.0 1

VAPA_HUMAN Vesicle-associated membrane protein-associated protein A 27.9/9.6 127.3 11.2 2

VAT1L_HUMAN Synaptic vesicle membrane protein VAT-1 homolog-like 45.9/4.8 54.1 4.8 2

VDAC1_HUMAN Voltage-dependent anion-selective channel protein 1 30.8/9.2 446.8 35.0 6

VDAC3_HUMAN Voltage-dependent anion-selective channel protein 3 30.6/9.5 241.2 19.1 3

VP13A_HUMAN Vacuolar protein sorting-associated protein 13A 360.0/5.9 58.6 0.5 2

VTA1_HUMAN Vacuolar protein sorting-associated protein VTA1 homolog 33.9/5.8 36.2 3.3 1

WDR62_HUMAN WD repeat-containing protein 62 165.8/5.5 97.8 3.2 1
