# Supplementary material for: Affinity purification-mass spectrometry analysis of bcl-2 interactome identified SLIRP as a novel interacting protein
Source: Cell Death Dis. 2016 Feb 11;7(2):e2090–. doi: 10.1038/cddis.2015.357 (PMC4849145; doi:10.1038/cddis.2015.357)
Supplement: Supplementary Table 2 [file cddis2015357x3.docx]

**Supplementary Table 2.** For each identified proteins details of identified peptides are reported. (P= number of missing cleavage; range= range of amino acid sequence)

**14-3-3 protein epsilon**

**Accession:** 1433E_HUMAN **Score:** 566.4

**MW [kDa]:** 29.2

**pI:** 4.5

**Sequence Coverage [%]:** 43.5

**No. of unique Peptides:** 9

**14-3-3 protein eta**

**Accession:** 1433F_HUMAN **Score:** 250.7

**MW [kDa]:** 28.2

**pI:** 4.6

**Sequence Coverage [%]:** 21.1

**No. of unique Peptides:** 4

**14-3-3 protein gamma**

**Accession:** 1433G_HUMAN **Score:** 393.7

**MW [kDa]:** 28.3

**pI:** 4.7

**Modification(s):** Oxidation **Sequence Coverage [%]:** 30.4

**HLA class I histocompatibility antigen**

**Accession:** 1B57_HUMAN **Score:** 58.4

**MW [kDa]:** 40.2

**pI:** 5.9

**Sequence Coverage [%]:** 9.1

**No. of unique Peptides:** 1

**Aspartate aminotransferase, mitochondrial**

**Accession:** AATM_HUMAN **Score:** 361.5

**MW [kDa]:** 47.5

**pI:** 9.8

**Modification(s):** Carbamidomethyl **Sequence Coverage [%]:** 19.5

**No. of unique Peptides:** 5

**ADP/ATP translocase 2**

**Accession:** ADT2_HUMAN **Score:** 564.8

**MW [kDa]:** 32.8

**pI:** 10.2

**Sequence Coverage [%]:** 37.2

**No. of unique Peptides:** 7

**ADP/ATP translocase 3**

**Accession:** ADT3_HUMAN **Score:** 516.1

**MW [kDa]:** 32.8

23 **pI:** 10.3

**Sequence Coverage [%]:** 37.2

**No. of unique Peptides:** 7

**Aminoacyl tRNA synthase complex-interacting multifunctional protein 1**

**Accession:** AIMP1_HUMAN **Score:** 52.0

**MW [kDa]:** 34.3

**pI:** 9.4

**Sequence Coverage [%]:** 3.8

**No. of unique Peptides:** 1

**Angiomotin**

**Accession:** AMOT_HUMAN **Score:** 76.6

**MW [kDa]:** 118.0

**pI:** 7.5

**Sequence Coverage [%]:** 2.0

**Annexin A11**

**Accession:** ANX11_HUMAN **Score:** 81.6

**MW [kDa]:** 54.4

**pI:** 8.5

**Modification(s):** Carbamidomethyl **Sequence Coverage [%]:** 7.3

**No. of unique Peptides:** 1

**Annexin A2 Accession:** ANXA2_HUMAN **Score:** 1081.8

**MW [kDa]:** 38.6

**pI:** 8.5

**Modification(s):** Carbamidomethyl **Sequence Coverage [%]:** 63.7

**No. of unique Peptides:** 13

**Annexin A4**

**Accession:** ANXA4_HUMAN **Score:** 37.0

**MW [kDa]:** 35.9

**pI:** 5.8

**Sequence Coverage [%]:** 5.0

**No. of unique Peptides:** 1

**ADP-ribosylation factor 4 Accession:** ARF4_HUMAN **Score:** 231.7

**MW [kDa]:** 20.5

**pI:** 7.5

**Sequence Coverage [%]:** 32.8

**No. of unique Peptides:** 5

**ADP-ribosylation factor 5**

**Accession:** ARF5_HUMAN **Score:** 214.1

**MW [kDa]:** 20.5

**pI:** 6.4

**Sequence Coverage [%]:** 28.9

**No. of unique Peptides:** 4

**Actin-related protein 3**

**Accession:** ARP3_HUMAN **Score:** 138.6

**MW [kDa]:** 47.3

**pI:** 5.5

**Sequence Coverage [%]:** 9.8

**No. of unique Peptides:** 1

**Actin-related protein 2/3 complex subunit 4**

**Accession:** ARPC4_HUMAN **Score:** 153.4

**MW [kDa]:** 19.7

**pI:** 9.4

**Sequence Coverage [%]:** 17.9

**No. of unique Peptides:** 2

**Sodium/potassium-transporting ATPase subunit beta-3**

**Accession:** AT1B3_HUMAN **Score:** 42.6

**MW [kDa]:** 31.5

**pI:** 9.3

**Sequence Coverage [%]:** 5.0

**No. of unique Peptides:** 1

**A disintegrin and metalloproteinase with thrombospondin motifs**

**Accession:** ATS10_HUMAN **Score:** 48.8

**MW [kDa]:** 120.8

**pI:** 9.7

**Sequence Coverage [%]:** 1.6

**BAG family molecular chaperone regulator 2**

**Accession:** BAG2_HUMAN **Score:** 69.5

**MW [kDa]:** 23.8

**pI:** 6.3

**Sequence Coverage [%]:** 9.5

**B-cell receptor-associated protein 31**

**Accession:** BAP31_HUMAN **Score:** 183.7

**MW [kDa]:** 28.0

**pI:** 9.1

**Sequence Coverage [%]:** 19.9

**No. of unique Peptides:** 2

**Apoptosis regulator Bcl-2**

**Accession:** BCL2_HUMAN **Score:** 164.0

**MW [kDa]:** 26.2

**pI:** 6.9

**Modification(s):** Oxidation **Sequence Coverage [%]:** 14.2

**No. of unique Peptides:** 2

**Calmodulin-like protein 5**

**Accession:** CALL5_HUMAN **Score:** 76.9

**MW [kDa]:** 15.9

**pI:** 4.1

**Sequence Coverage [%]:** 21.2

**No. of unique Peptides:** 1

**Calmodulin**

**Accession:** CALM_HUMAN **Score:** 90.9

**MW [kDa]:** 16.8

**pI:** 3.9

**Sequence Coverage [%]:** 29.5

**No. of unique Peptides:** 0

**Adenylyl cyclase-associated protein 1**

**Accession:** CAP1_HUMAN **Score:** 157.5

**MW [kDa]:** 51.9

**pI:** 9.1

**Modification(s):** Carbamidomethyl **Sequence Coverage [%]:** 16.8

**No. of unique Peptides:** 1

**F-actin-capping protein subunit beta**

**Accession:** CAPZB_HUMAN **Score:** 167.7

**MW [kDa]:** 31.3

**pI:** 5.2

**Modification(s):** Carbamidomethyl **Sequence Coverage [%]:** 15.2

**No. of unique Peptides:** 3

**Protein 482: Catalase - Homo sapiens (Human)**

**Accession:** CATA_HUMAN **Score:** 32.8

**Database:** SwissProt(SwissProt_55.3.fasta) **MW [kDa]:** 59.7

**Database Date:** 2008-09-07 **pI:** 7.0

**Sequence Coverage [%]:** 2.5

**No. of unique Peptides:** 0

**Protein 245: Centrosomal protein of 290 kDa OS=Homo sapiens GN=CEP290 PE=1 SV=2**

**Accession:** CE290_HUMAN **Score:** 146.7

**Database:** SwissProt(SwissProt_56.1.fasta) **MW [kDa]:** 290.2

**Database Date:** 2008-10-22 **pI:** 5.7

**Sequence Coverage [%]:** 2.9

**No. of unique Peptides:** 0

**Centromere protein F**

**Accession:** CENPF_HUMAN **Score:** 185.7

**MW [kDa]:** 367.5

**pI:** 4.9

**Modification(s):** Oxidation **Sequence Coverage [%]:** 3.4

**No. of unique Peptides:** 1

**Coiled-coil-helix-coiled-coil-helix domain-containing protein 3, mitochondrial**

**Accession:** CHCH3_HUMAN **Score:** 110.4

**MW [kDa]:** 26.1

**pI:** 9.3

**Sequence Coverage [%]:** 11.5

**No. of unique Peptides:** 2

**Protein 478: Cysteine and histidine-rich domain-containing protein 1 OS=Homo sapiens GN=**

**CHORDC1 PE=1 SV=2**

**Accession:** CHRD1_HUMAN **Score:** 33.6

**Database:** SwissProt(SwissProt_56.1.fasta) **MW [kDa]:** 37.5

**Database Date:** 2008-10-22 **pI:** 9.2

**Sequence Coverage [%]:** 4.5

**No. of unique Peptides:** 0

**Citrate synthase, mitochondrial**

**Accession:** CISY_HUMAN **Score:** 97.9

**MW [kDa]:** 51.7

**pI:** 9.1

**Sequence Coverage [%]:** 10.3

**No. of unique Peptides:** 4

**Clathrin light chain A**

**Accession:** CLCA_HUMAN **Score:** 188.9

**MW [kDa]:** 27.1

**pI:** 4.3

**Sequence Coverage [%]:** 13.7

**No. of unique Peptides:** 3

**Clathrin light chain B**

**Accession:** CLCB_HUMAN **Score:** 109.6

**MW [kDa]:** 25.2

**pI:** 4.4

**Modification(s):** Carbamidomethyl **Sequence Coverage [%]:** 11.8

**No. of unique Peptides:** 2

**Dual specificity protein kinase CLK3**

**Accession:** CLK3_HUMAN **Score:** 35.6

**MW [kDa]:** 73.5

**pI:** 10.7

**Sequence Coverage [%]:** 3.1

**No. of unique Peptides:** 1

**Calponin-3 Accession:** CNN3_HUMAN **Score:** 210.9

**MW [kDa]:** 36.4

**pI:** 5.6

**Sequence Coverage [%]:** 20.4

**No. of unique Peptides:** 2

**Cofilin-1 Accession:** COF1_HUMAN **Score:** 175.6

**MW [kDa]:** 18.5

**pI:** 9.1

**Sequence Coverage [%]:** 30.1

**No. of unique Peptides:** 3

**Coactosin-like protein**

**Accession:** COTL1_HUMAN **Score:** 64.8

**MW [kDa]:** 15.9

**pI:** 5.4

**Sequence Coverage [%]:** 10.6

**No. of unique Peptides:** 1

**Cytochrome c oxidase subunit 2**

**Accession:** COX2_HUMAN **Score:** 112.7

**MW [kDa]:** 25.5

**pI:** 4.5

**Sequence Coverage [%]:** 11.9

**No. of unique Peptides:** 2

**Cytochrome c oxidase subunit 5A, mitochondrial**

**Accession:** COX5A_HUMAN **Score:** 68.9

**MW [kDa]:** 16.8

**pI:** 6.4

**Sequence Coverage [%]:** 20.7

**No. of unique Peptides:** 2

**Cytochrome c oxidase subunit 6C**

**Accession:** COX6C_HUMAN **Score:** 39.1

**MW [kDa]:** 8.8

**pI:** 10.8

**Sequence Coverage [%]:** 10.7

**No. of unique Peptides:** 1

**Homeobox protein cut-like 2**

**Accession:** CUX2_HUMAN **Score:** 91.9

**MW [kDa]:** 161.6

**pI:** 5.3

**Modification(s):** Oxidation **Sequence Coverage [%]:** 2.6

**No. of unique Peptides:** 1

**Calcyclin-binding protein**

**Accession:** CYBP_HUMAN **Score:** 183.3

**MW [kDa]:** 26.2

**pI:** 9.0

**Modification(s):** Carbamidomethyl **Sequence Coverage [%]:** 38.2

**No. of unique Peptides:** 2

**Diablo homolog, mitochondrial**

**Accession:** DBLOH_HUMAN **Score:** 68.2

**MW [kDa]:** 27.1

**pI:** 5.6

**Sequence Coverage [%]:** 4.2

**No. of unique Peptides:** 1

**Protein 396: Probable ATP-dependent RNA helicase DDX6 OS=Homo sapiens GN=DDX6 PE=1 SV=2**

**Accession:** DDX6_HUMAN **Score:** 69.0

**Database:** SwissProt(SwissProt_2011_08.fasta) **MW [kDa]:** 54.4

**Database Date:** 2011-09-14 **pI:** 9.6

**Sequence Coverage [%]:** 6.4

**No. of unique Peptides:** 0

**Destrin**

**Accession:** DEST_HUMAN **Score:** 35.4

**MW [kDa]:** 18.5

**pI:** 9.2

**Sequence Coverage [%]:** 6.7

**No. of unique Peptides:** 1

**Succinate dehydrogenase [ubiquinone] flavoprotein subunit, mitochondrial**

**Accession:** DHSA_HUMAN **Score:** 50.3

**MW [kDa]:** 72.6

**pI:** 7.3

**Sequence Coverage [%]:** 2.1

**No. of unique Peptides:** 1

**Protein 390: Succinate dehydrogenase [ubiquinone] iron-sulfur subunit, mitochondrial OS=Homo**

**sapiens GN=SDHB PE=1 SV=3**

**Accession:** DHSB_HUMAN **Score:** 70.2

**Database:** SwissProt(SwissProt_2011_08.fasta) **MW [kDa]:** 31.6

**Database Date:** 2011-09-14 **pI:** 9.9

**Sequence Coverage [%]:** 11.4

**No. of unique Peptides:** 0

**Protein dopey-2**

**Accession:** DOP2_HUMAN **Score:** 111.5

**MW [kDa]:** 258.1

**pI:** 5.9

**Sequence Coverage [%]:** 1.7

**No. of unique Peptides:** 1

**Dolichol-phosphate mannosyltransferase**

**Accession:** DPM1_HUMAN **Score:** 40.7

**MW [kDa]:** 29.6

**pI:** 10.0

**Sequence Coverage [%]:** 4.2

**No. of unique Peptides:** 1

**Developmentally-regulated GTP-binding protein 1**

**Accession:** DRG1_HUMAN **Score:** 161.5

**MW [kDa]:** 40.5

**pI:** 9.6

**Sequence Coverage [%]:** 13.4

**No. of unique Peptides:** 2

**Spliceosome RNA helicase DDX39B**

**Accession:** DX39B_HUMAN **Score:** 117.3

**MW [kDa]:** 49.0

**pI:** 5.3

**Sequence Coverage [%]:** 8.6

**No. of unique Peptides:** 1

**Protein 360: Dynein heavy chain 12, axonemal OS=Homo sapiens GN=DNAH12 PE=2 SV=2**

**Accession:** DYH12_HUMAN **Score:** 83.4

**Database:** SwissProt(SwissProt_57.15.fasta) **MW [kDa]:** 356.7

**Database Date:** 2011-05-23 **pI:** 5.8

**Sequence Coverage [%]:** 1.9

**No. of unique Peptides:** 0

**Enoyl-CoA delta isomerase 1, mitochondrial**

**Accession:** ECI1_HUMAN **Score:** 105.2

**MW [kDa]:** 32.8

**pI:** 9.7

**Sequence Coverage [%]:** 9.3

**No. of unique Peptides:** 2

**Eukaryotic translation initiation factor 2A OS=Homo sapiens GN=EIF2A PE=1 SV=3**

**Accession:** EIF2A_HUMAN **Score:** 87.7

**MW [kDa]:** 64.9

**pI:** 9.6

**Sequence Coverage [%]:** 5.0

**No. of unique Peptides:** 2

**Protein 415: Eukaryotic translation initiation factor 3 subunit E OS=Homo sapiens GN=EIF3E PE=1**

**SV=1**

**Accession:** EIF3E_HUMAN **Score:** 63.5

**Database:** SwissProt(SwissProt_2011_08.fasta) **MW [kDa]:** 52.2

**Database Date:** 2011-09-14 **pI:** 5.7

**Sequence Coverage [%]:** 7.2

**No. of unique Peptides:** 0

**Eukaryotic translation initiation factor 3 subunit F SV=1**

**Accession:** EIF3F_HUMAN **Score:** 89.1

**MW [kDa]:** 37.5

**pI:** 5.1

**Sequence Coverage [%]:** 10.1

**No. of unique Peptides:** 1

**Eukaryotic translation initiation factor 3 subunit M**

**Accession:** EIF3M_HUMAN **Score:** 71.1

**MW [kDa]:** 42.5

**pI:** 5.3

**Modification(s):** Carbamidomethyl **Sequence Coverage [%]:** 5.1

**No. of unique Peptides:** 1

**Eukaryotic peptide chain release factor subunit 1**

**Accession:** ERF1_HUMAN **Score:** 132.8

**MW [kDa]:** 49.0

**pI:** 5.4

**Sequence Coverage [%]:** 9.4

**No. of unique Peptides:** 1

**Endoplasmic reticulum resident protein 29 Accession:** ERP29_HUMAN **Score:** 84.3

**MW [kDa]:** 29.0

**pI:** 7.5

**Sequence Coverage [%]:** 8.4

**No. of unique Peptides:** 2

**Protein 317: Protein FAM49B OS=Homo sapiens GN=FAM49B PE=1 SV=1**

**Accession:** FA49B_HUMAN **Score:** 102.5

**Database:** SwissProt(SwissProt_2011_08.fasta) **MW [kDa]:** 36.7

**Database Date:** 2011-09-14 **pI:** 5.7

**Sequence Coverage [%]:** 12.7

**No. of unique Peptides:** 0

**Protein 380: Coagulation factor VII OS=Homo sapiens GN=F7 PE=1 SV=1**

**Accession:** FA7_HUMAN **Score:** 74.3

**Database:** SwissProt(SwissProt_2011_08.fasta) **MW [kDa]:** 51.6

**Database Date:** 2011-09-14 **pI:** 7.2

**Sequence Coverage [%]:** 5.8

**No. of unique Peptides:** 0

**Protein 423: Protein FAM3C OS=Homo sapiens GN=FAM3C PE=1 SV=1**

**Accession:** FAM3C_HUMAN **Score:** 59.1

**Database:** SwissProt(SwissProt_2011_08.fasta) **MW [kDa]:** 24.7

**Database Date:** 2011-09-14 **pI:** 9.4

**Sequence Coverage [%]:** 12.3

**No. of unique Peptides:** 0

**Protein 446: Fanconi anemia group J protein OS=Homo sapiens GN=BRIP1 PE=1 SV=1**

**Accession:** FANCJ_HUMAN **Score:** 49.5

**Database:** SwissProt(SwissProt_56.1.fasta) **MW [kDa]:** 140.8

**Database Date:** 2008-10-22 **pI:** 6.5

**Sequence Coverage [%]:** 1.9

**No. of unique Peptides:** 0

**Four and a half LIM domains protein 1 Accession:** FHL1_HUMAN **Score:** 80.6

**MW [kDa]:** 36.2

**pI:** 10.5

**Sequence Coverage [%]:** 5.6

**No. of unique Peptides:** 1

**Fibrous sheath-interacting protein 2 OS=Homo sapiens GN=FSIP2 PE=1 SV=4**

**Accession:** FSIP2_HUMAN **Score:** 69.5

**MW [kDa]:** 780.1

**Database Date:** 2011-09-14 **pI:** 6.3

**Sequence Coverage [%]:** 0.3

**No. of unique Peptides:** 0

**1-phosphatidylinositol-3-phosphate 5-kinase**

**Accession:** FYV1_HUMAN **Score:** 71.8

**MW [kDa]:** 237.0

**pI:** 6.2

**Sequence Coverage [%]:** 0.9

**No. of unique Peptides:** 2

**Glucose-6-phosphate isomerase**

**Accession:** G6PI_HUMAN **Score:** 281.9

**MW [kDa]:** 63.1

**pI:** 9.1

**Sequence Coverage [%]:** 18.3

**No. of unique Peptides:** 3

**Protein 441: Polypeptide N-acetylgalactosaminyltransferase 6 OS=Homo sapiens GN=GALNT6 PE=**

**2 SV=2**

**Accession:** GALT6_HUMAN **Score:** 51.6

**Database:** SwissProt(SwissProt_2011_08.fasta) **MW [kDa]:** 71.1

**Database Date:** 2011-09-14 **pI:** 9.4

**Sequence Coverage [%]:** 2.3

**No. of unique Peptides:** 0

**Rab GDP dissociation inhibitor beta**

**Accession:** GDIB_HUMAN **Score:** 87.5

**MW [kDa]:** 50.6

**pI:** 6.1

**Sequence Coverage [%]:** 9.0

**No. of unique Peptides:** 1

**Glucosamine--fructose-6-phosphate aminotransferase [isomerizing] 1**

**Accession:** GFPT1_HUMAN **Score:** 78.9

**MW [kDa]:** 78.8

**pI:** 6.7

**Sequence Coverage [%]:** 3.4

**No. of unique Peptides:** 1

**Serine hydroxymethyltransferase, mitochondrial**

**Accession:** GLYM_HUMAN **Score:** 83.3

**MW [kDa]:** 56.0

**pI:** 9.5

**Sequence Coverage [%]:** 6.7

**No. of unique Peptides:** 1

**G-rich sequence factor 1**

**Accession:** GRSF1_HUMAN **Score:** 90.4

**MW [kDa]:** 53.1

**pI:** 5.8

**Sequence Coverage [%]:** 8.1

**No. of unique Peptides:** 1

**HAUS augmin-like complex subunit 5**

**Accession:** HAUS5_HUMAN **Score:** 39.4

**MW [kDa]:** 71.6

**pI:** 9.9

**Sequence Coverage [%]:** 1.9

**No. of unique Peptides:** 1

**Heterogeneous nuclear ribonucleoprotein Q**

**Accession:** HNRPQ_HUMAN **Score:** 79.9

**MW [kDa]:** 69.6

**pI:** 9.1

**Sequence Coverage [%]:** 5.5

**No. of unique Peptides:** 3

**Protein 477: Homeobox protein Hox-A6 OS=Homo sapiens GN=HOXA6 PE=2 SV=2**

**Accession:** HXA6_HUMAN **Score:** 34.2

**Database:** SwissProt(SwissProt_2011_08.fasta) **MW [kDa]:** 26.3

**Database Date:** 2011-09-14 **pI:** 9.8

**Sequence Coverage [%]:** 6.0

**No. of unique Peptides:** 0

**Isocitrate dehydrogenase [NAD] subunit alpha, mitochondrial**

**Accession:** IDH3A_HUMAN **Score:** 106.8

**MW [kDa]:** 39.6

**pI:** 6.5

**Sequence Coverage [%]:** 12.3

**No. of unique Peptides:** 1

**Eukaryotic translation initiation factor 2A Accession:** EIF2A_HUMAN **Score:** 87.7

**MW [kDa]:** 64.9

**pI:** 9.6

**Sequence Coverage [%]:** 5.0

**No. of unique Peptides:** 2

**Eukaryotic translation initiation factor 2 subunit 3**

**Accession:** IF2G_HUMAN **Score:** 139.7

**MW [kDa]:** 51.1

**pI:** 9.5

**Sequence Coverage [%]:** 8.9

**No. of unique Peptides:** 3

**Protein 336: Eukaryotic initiation factor 4A-III OS=Homo sapiens GN=EIF4A3 PE=1 SV=4**

**Accession:** IF4A3_HUMAN **Score:** 93.5

**Database:** SwissProt(SwissProt_2011_08.fasta) **MW [kDa]:** 46.8

**Database Date:** 2011-09-14 **pI:** 6.3

**Sequence Coverage [%]:** 8.3

**No. of unique Peptides:** 0

**Eukaryotic translation initiation factor 4E**

**Accession:** IF4E_HUMAN **Score:** 41.7

**MW [kDa]:** 25.1

**pI:** 5.8

**Modification(s):** Carbamidomethyl **Sequence Coverage [%]:** 5.1

**No. of unique Peptides:** 1

**Eukaryotic translation initiation factor 4H**

**Accession:** IF4H_HUMAN **Score:** 144.3

**MW [kDa]:** 27.4

**pI:** 7.5

**Sequence Coverage [%]:** 14.1

**No. of unique Peptides:** 2

**Eukaryotic translation initiation factor 5A-1**

**Accession:** IF5A1_HUMAN **Score:** 115.5

**MW [kDa]:** 16.8

**pI:** 4.9

**Sequence Coverage [%]:** 12.3

**No. of unique Peptides:** 1

**Eukaryotic translation initiation factor 6**

**Accession:** IF6_HUMAN **Score:** 93.8

**MW [kDa]:** 26.6

**pI:** 4.4

**Modification(s):** Carbamidomethyl **Sequence Coverage [%]:** 21.2

**No. of unique Peptides:** 2

**Intraflagellar transport protein 57 homolog**

**Accession:** IFT57_HUMAN **Score:** 56.8

**MW [kDa]:** 49.1

**pI:** 4.8

**Modification(s):** Oxidation **Sequence Coverage [%]:** 6.3

**No. of unique Peptides:** 2

**Importin subunit alpha-1**

**Accession:** IMA1_HUMAN **Score:** 194.6

**MW [kDa]:** 57.8

**pI:** 5.1

**Sequence Coverage [%]:** 9.8

**No. of unique Peptides:** 3

**Junctophilin-3 Accession:** JPH3_HUMAN **Score:** 35.9

**MW [kDa]:** 81.4

**pI:** 10.0

**Sequence Coverage [%]:** 1.6

**No. of unique Peptides:** 1

**Creatine kinase B-type**

**Accession:** KCRB_HUMAN **Score:** 82.9

**MW [kDa]:** 42.6

**pI:** 5.2

**Sequence Coverage [%]:** 8.9

**No. of unique Peptides:** 1

**Protein 432: Chromosome-associated kinesin KIF4B OS=Homo sapiens GN=KIF4B PE=1 SV=2**

**Accession:** KIF4B_HUMAN **Score:** 54.3

**Database:** SwissProt(SwissProt_56.1.fasta) **MW [kDa]:** 139.9

**Database Date:** 2008-10-22 **pI:** 5.8

**Sequence Coverage [%]:** 1.1

**No. of unique Peptides:** 0

**L-lactate dehydrogenase B**

**Accession:** LDHB_HUMAN **Score:** 616.7

**MW [kDa]:** 36.6

**pI:** 5.7

**Modification(s):** Carbamidomethyl **Sequence Coverage [%]:** 30.8

**No. of unique Peptides:** 8

**Platelet-activating factor acetylhydrolase IB subunit alpha**

**Accession:** LIS1_HUMAN **Score:** 58.6

**MW [kDa]:** 46.6

**pI:** 7.2

**Sequence Coverage [%]:** 5.6

**No. of unique Peptides:** 1

**Leucine-rich repeat-containing protein 8E**

**Accession:** LRC8E_HUMAN **Score:** 62.2

**MW [kDa]:** 90.2

**pI:** 6.5

**Modification(s):** Carbamidomethyl **Sequence Coverage [%]:** 2.0

**No. of unique Peptides:** 1

**Protein LSM12 homolog**

**Accession:** LSM12_HUMAN **Score:** 35.7

**MW [kDa]:** 21.7

**pI:** 8.9

**Sequence Coverage [%]:** 6.7

**No. of unique Peptides:** 1

**Protein 411: Latent-transforming growth factor beta-binding protein 2 OS=Homo sapiens GN=**

**LTBP2 PE=1 SV=3**

**Accession:** LTBP2_HUMAN **Score:** 64.7

**Database:** SwissProt(SwissProt_2011_08.fasta) **MW [kDa]:** 194.9

**Database Date:** 2011-09-14 **pI:** 4.9

**Sequence Coverage [%]:** 1.9

**No. of unique Peptides:** 0

**Myristoylated alanine-rich C-kinase substrate**

**Accession:** MARCS_HUMAN **Score:** 110.5

**MW [kDa]:** 31.5

**pI:** 4.3

**Sequence Coverage [%]:** 14.5

**No. of unique Peptides:** 2

**Microsomal glutathione S-transferase 3**

**Accession:** MGST3_HUMAN **Score:** 35.0

**MW [kDa]:** 16.5

**pI:** 10.1

**Sequence Coverage [%]:** 8.6

**No. of unique Peptides:** 1

**Macrophage migration inhibitory factor**

**Accession:** MIF_HUMAN **Score:** 73.3

**MW [kDa]:** 12.5

**Modification(s):** Carbamidomethyl **Sequence Coverage [%]:** 17.4

**No. of unique Peptides:** 2

**Max-like protein X**

**Accession:** MLX_HUMAN **Score:** 64.5

**MW [kDa]:** 33.3

**pI:** 9.1

**Sequence Coverage [%]:** 7.4

**No. of unique Peptides:** 3

**MOB kinase activator 1A**

**Accession:** MOB1A_HUMAN **Score:** 85.8

**MW [kDa]:** 25.1

**pI:** 6.5

**Sequence Coverage [%]:** 11.1

**No. of unique Peptides:** 1

**Moesin Accession:** MOES_HUMAN **Score:** 135.9

**MW [kDa]:** 67.8

**pI:** 6.0

**Sequence Coverage [%]:** 6.2

**No. of unique Peptides:** 2

**Protein 319: Myosin-11 OS=Homo sapiens GN=MYH11 PE=1 SV=3**

**Accession:** MYH11_HUMAN **Score:** 102.3

**Database:** SwissProt(SwissProt_56.1.fasta) **MW [kDa]:** 227.2

**Database Date:** 2008-10-22 **pI:** 5.3

**Sequence Coverage [%]:** 1.6

**No. of unique Peptides:** 0

**Myosin light polypeptide 6**

**Accession:** MYL6_HUMAN **Score:** 273.2

**MW [kDa]:** 16.9

**pI:** 4.4

**Modification(s):** Carbamidomethyl, Oxidation **Sequence Coverage [%]:** 47.7

**No. of unique Peptides:** 4

**N-acetylated-alpha-linked acidic dipeptidase 2**

**Accession:** NALD2_HUMAN **Score:** 49.2

**MW [kDa]:** 83.5

**pI:** 9.0

**Sequence Coverage [%]:** 1.8

**No. of unique Peptides:** 1

**Neutral cholesterol ester hydrolase 1**

**Accession:** NCEH1_HUMAN **Score:** 60.3

**MW [kDa]:** 45.8

**pI:** 6.9

**Sequence Coverage [%]:** 6.9 **No. of unique Peptides:** 2

**NADH dehydrogenase [ubiquinone] iron-sulfur protein 3,**

**Accession:** NDUS3_HUMAN **Score:** 74.9

**MW [kDa]:** 30.2

**pI:** 7.8

**Sequence Coverage [%]:** 9.8

**No. of unique Peptides:** 2

**NADH dehydrogenase [ubiquinone] iron-sulfur protein 8,**

**Accession:** NDUS8_HUMAN **Score:** 77.0

**MW [kDa]:** 23.7

**pI:** 6.0

**Sequence Coverage [%]:** 9.5

**No. of unique Peptides:** 1

**Protein 118: Transcriptional repressor NF-X1 OS=Homo sapiens GN=NFX1 PE=1 SV=2**

**Accession:** NFX1_HUMAN **Score:** 34.1

**Database: MW [kDa]:** 124.3

**pI:** 10.2

**Sequence Coverage [%]:** 0.7

**No. of unique Peptides:** 1

**Non-POU domain-containing octamer-binding protein**

**Accession:** NONO_HUMAN **Score:** 351.1

**MW [kDa]:** 54.2

**pI:** 9.6

**Sequence Coverage [%]:** 23.4

**No. of unique Peptides:** 2

**Nucleosome assembly protein 1-like 1**

**Accession:** NP1L1_HUMAN **Score:** 187.1

**MW [kDa]:** 45.3

**pI:** 4.2

**Sequence Coverage [%]:** 12.5

**No. of unique Peptides:** 3

**Protein 406: Sphingomyelin phosphodiesterase 2 OS=Homo sapiens GN=SMPD2 PE=1 SV=2**

**Accession:** NSMA_HUMAN **Score:** 65.7

**Database:** SwissProt(SwissProt_2011_08.fasta) **MW [kDa]:** 47.6

**Database Date:** 2011-09-14 **pI:** 6.5

**Sequence Coverage [%]:** 7.3

**No. of unique Peptides:** 0

**Protein 400: OCIA domain-containing protein 1 OS=Homo sapiens GN=OCIAD1 PE=1 SV=1**

**Accession:** OCAD1_HUMAN **Score:** 67.7

**Database:** SwissProt(SwissProt_2011_08.fasta) **MW [kDa]:** 27.6

**Database Date:** 2011-09-14 **pI:** 7.8

**Sequence Coverage [%]:** 13.5

**No. of unique Peptides:** 0

**Obg-like ATPase 1**

**Accession:** OLA1_HUMAN **Score:** 76.3

**MW [kDa]:** 44.7

**pI:** 8.6

**Sequence Coverage [%]:** 3.8

**No. of unique Peptides:** 1

**Dolichyl-diphosphooligosaccharide--protein glycosyltransferase 48 kDa subunit**

**Accession:** OST48_HUMAN **Score:** 61.9

**MW [kDa]:** 50.8

**pI:** 6.1

**Sequence Coverage [%]:** 4.8

**No. of unique Peptides:** 2

**Osteocalcin OS=Homo sapiens GN=BGLAP PE=1 SV=2**

**Accession:** OSTCN_HUMAN **Score:** 32.6

**Database:** SwissProt(SwissProt_2011_08.fasta) **MW [kDa]:** 11.0

**Database Date:** 2011-09-14 **pI:** 7.5

**Sequence Coverage [%]:** 15.0

**No. of unique Peptides:** 0

**Programmed cell death protein 5**

**Accession:** PDCD5_HUMAN **Score:** 135.0

**MW [kDa]:** 14.3

**pI:** 5.7

**Sequence Coverage [%]:** 19.2

**No. of unique Peptides:** 2

**Programmed cell death protein 6**

**Accession:** PDCD6_HUMAN **Score:** 71.3

**MW [kDa]:** 21.9

**pI:** 5.0

**Sequence Coverage [%]:** 12.0

**No. of unique Peptides:** 1

**Phosphoglycerate kinase 1**

**Accession:** PGK1_HUMAN **Score:** 810.9

**MW [kDa]:** 44.6

**pI:** 9.2

**Modification(s):** Carbamidomethyl **Sequence Coverage [%]:** 50.1

**No. of unique Peptides:** 10

**Protein 359: Phosphatidylinositol 4-kinase alpha OS=Homo sapiens GN=PI4KA PE=1 SV=3**

**Accession:** PI4KA_HUMAN **Score:** 83.9

**Database:** SwissProt(SwissProt_2011_08.fasta) **MW [kDa]:** 231.2

**Database Date:** 2011-09-14 **pI:** 6.4

**Sequence Coverage [%]:** 2.0

**No. of unique Peptides:** 0

**Peptidyl-prolyl cis-trans isomerase A**

**Accession:** PPIA_HUMAN **Score:** 130.8

**MW [kDa]:** 18.0

**pI:** 9.0

**Modification(s):** Carbamidomethyl **Sequence Coverage [%]:** 30.3

**No. of unique Peptides:** 4

**Peroxiredoxin-1**

**Accession:** PRDX1_HUMAN **Score:** 85.6

**MW [kDa]:** 22.1

**pI:** 9.2

**Sequence Coverage [%]:** 14.1

**No. of unique Peptides:** 3

**Thioredoxin-dependent peroxide reductase, mitochondrial**

**Accession:** PRDX3_HUMAN **Score:** 134.6

**MW [kDa]:** 27.7

**pI:** 8.9

**Sequence Coverage [%]:** 10.2

**No. of unique Peptides:** 2

**Protein 476: 26S protease regulatory subunit S10B OS=Homo sapiens GN=PSMC6 PE=1 SV=1**

**Accession:** PRS10_HUMAN **Score:** 34.6

**Database:** SwissProt(SwissProt_56.1.fasta) **MW [kDa]:** 44.1

**Database Date:** 2008-10-22 **pI:** 7.8

**Sequence Coverage [%]:** 4.4

**No. of unique Peptides:** 1

**26S protease regulatory subunit 4**

**Accession:** PRS4_HUMAN **Score:** 63.0

**MW [kDa]:** 49.2

**pI:** 5.8

**Sequence Coverage [%]:** 2.7

**No. of unique Peptides:** 1

**Protein 367: 26S protease regulatory subunit 7 OS=Homo sapiens GN=PSMC2 PE=1 SV=3**

**Accession:** PRS7_HUMAN **Score:** 80.8

**Database:** SwissProt(SwissProt_56.1.fasta) **MW [kDa]:** 48.6

**Database Date:** 2008-10-22 **pI:** 5.6

**Sequence Coverage [%]:** 6.7

**No. of unique Peptides:** 0

**Proteasome subunit alpha type-1**

**Accession:** PSA1_HUMAN **Score:** 88.4

**MW [kDa]:** 29.5

**pI:** 6.2

**Sequence Coverage [%]:** 12.9

**No. of unique Peptides:** 1

**Proteasome subunit alpha type-2**

**Accession:** PSA2_HUMAN **Score:** 81.5

**MW [kDa]:** 25.9

**pI:** 7.7

**Sequence Coverage [%]:** 16.7

**No. of unique Peptides:** 1

**Proteasome subunit alpha type-4 Accession:** PSA4_HUMAN **Score:** 172.1

**MW [kDa]:** 29.5

**pI:** 8.7

**Sequence Coverage [%]:** 16.5

**No. of unique Peptides:** 1

**Proteasome subunit alpha type-5**

**Accession:** PSA5_HUMAN **Score:** 285.0

**MW [kDa]:** 26.4

**pI:** 4.6

**Sequence Coverage [%]:** 29.5

**No. of unique Peptides:** 4

**Proteasome subunit beta type-5**

**Accession:** PSB5_HUMAN **Score:** 40.0

**MW [kDa]:** 28.5

**pI:** 6.5

**Sequence Coverage [%]:** 3.8

**No. of unique Peptides:** 1

**26S proteasome non-ATPase regulatory subunit 3**

**Accession:** PSMD3_HUMAN **Score:** 103.7

**MW [kDa]:** 60.9

**pI:** 9.0

**Sequence Coverage [%]:** 8.1

**No. of unique Peptides:** 1

**Proteasome activator complex subunit 1 Accession:** PSME1_HUMAN **Score:** 113.0

**MW [kDa]:** 28.7

**pI:** 5.7

**Sequence Coverage [%]:** 12.4

**No. of unique Peptides:** 1

**Cytochrome b-c1 complex subunit 7**

**Accession:** QCR7_HUMAN **Score:** 41.8

**MW [kDa]:** 13.5

**pI:** 9.2

**Sequence Coverage [%]:** 11.7

**No. of unique Peptides:** 1

**Heterogeneous nuclear ribonucleoprotein A1-like 2**

**Accession:** RA1L2_HUMAN **Score:** 38.5

**MW [kDa]:** 34.2

**pI:** 9.6

**Sequence Coverage [%]:** 3.1

**No. of unique Peptides:** 1

**Ras-related protein Rab-10**

**Accession:** RAB10_HUMAN **Score:** 119.6

**MW [kDa]:** 22.5

**pI:** 9.4

**Sequence Coverage [%]:** 16.5

**No. of unique Peptides:** 3

**Ras-related protein Rab-18**

**Accession:** RAB18_HUMAN **Score:** 134.9

**MW [kDa]:** 23.0

**pI:** 5.0

**Sequence Coverage [%]:** 24.3

**No. of unique Peptides:** 2

**Ras-related protein Rab-21**

**Accession:** RAB21_HUMAN **Score:** 84.4

**MW [kDa]:** 24.3

**pI:** 9.2

**Sequence Coverage [%]:** 20.4

**No. of unique Peptides:** 1

**Rab proteins geranylgeranyltransferase component A 1**

**Accession:** RAE1_HUMAN **Score:** 36.4

**MW [kDa]:** 73.4

**pI:** 4.5

**Sequence Coverage [%]:** 1.4

**No. of unique Peptides:** 1

**Ran-specific GTPase-activating protein**

**Accession:** RANG_HUMAN **Score:** 120.7

**MW [kDa]:** 23.3

**pI:** 5.1

**Sequence Coverage [%]:** 14.9

**No. of unique Peptides:** 2

**Ras-related protein Rap-1b**

**Accession:** RAP1B_HUMAN **Score:** 237.8

**MW [kDa]:** 20.8

**pI:** 5.5

**Sequence Coverage [%]:** 34.8

**No. of unique Peptides:** 3

**Ras-related protein Rap-2b**

**Accession:** RAP2B_HUMAN **Score:** 70.2

**MW [kDa]:** 20.5

**pI:** 4.6

**Sequence Coverage [%]:** 6.6

**No. of unique Peptides:** 1

**GTPase NRas Accession:** RASN_HUMAN **Score:** 51.8

**Database:** SwissProt(SwissProt_2011_08.fasta) **MW [kDa]:** 21.2

**Database Date:** 2011-09-14 **pI:** 4.9

**Sequence Coverage [%]:** 6.3

**No. of unique Peptides:** 1

**Ras-related protein Rab-11A**

**Accession:** RB11A_HUMAN **Score:** 74.2

**MW [kDa]:** 24.4

**pI:** 6.1

**Sequence Coverage [%]:** 10.6

**No. of unique Peptides:** 2

**Ras-related protein Rab-11B**

**Accession:** RB11B_HUMAN **Score:** 381.5

**MW [kDa]:** 24.5

**pI:** 5.6

**Sequence Coverage [%]:** 42.2

**No. of unique Peptides:** 5

**Protein 414: Histone-binding protein RBBP4 OS=Homo sapiens GN=RBBP4 PE=1 SV=3**

**Accession:** RBBP4_HUMAN **Score:** 63.7

**Database:** SwissProt(SwissProt_56.1.fasta) **MW [kDa]:** 47.6

**Database Date:** 2008-10-22 **pI:** 4.6

**Sequence Coverage [%]:** 3.5

**No. of unique Peptides:** 0

**RNA-binding motif protein, X chromosome**

**Accession:** RBMX_HUMAN **Score:** 211.8

**MW [kDa]:** 42.3

**pI:** 0.0

**Sequence Coverage [%]:** 17.1

**No. of unique Peptides:** 3

**60S ribosomal protein L10 Accession:** RL10_HUMAN **Score:** 287.2

**MW [kDa]:** 24.6

**pI:** 11.0

**Modification(s):** Oxidation **Sequence Coverage [%]:** 25.2

**No. of unique Peptides:** 3

**60S ribosomal protein L21**

**Accession:** RL21_HUMAN **Score:** 118.5

**MW [kDa]:** 18.6

**pI:** 11.0

**Sequence Coverage [%]:** 31.9

**No. of unique Peptides:** 1

**60S ribosomal protein L36 Accession:** RL36_HUMAN **Score:** 119.5

**MW [kDa]:** 12.2

**pI:** 12.2

**Sequence Coverage [%]:** 28.6

**No. of unique Peptides:** 2

**39S ribosomal protein L1, mitochondrial**

**Accession:** RM01_HUMAN **Score:** 51.7

**MW [kDa]:** 36.9

**pI:** 9.5

**Sequence Coverage [%]:** 7.4

**No. of unique Peptides:** 1

**Regulator of microtubule dynamics protein 1**

**Accession:** RMD1_HUMAN **Score:** 40.9

**MW [kDa]:** 35.8

**pI:** 9.2

**Sequence Coverage [%]:** 2.9

**No. of unique Peptides:** 1

**Regulator of microtubule dynamics protein 1**

**Accession:** RMD1_HUMAN **Score:** 40.9

**MW [kDa]:** 35.8

**pI:** 9.2

**Sequence Coverage [%]:** 2.9

**No. of unique Peptides:** 1

**Heterogeneous nuclear ribonucleoprotein A0**

**Accession:** ROA0_HUMAN **Score:** 64.4

**MW [kDa]:** 30.8

**pI:** 9.8

**Sequence Coverage [%]:** 8.9

**No. of unique Peptides:** 1

**Heterogeneous nuclear ribonucleoprotein A3**

**Accession:** ROA3_HUMAN **Score:** 164.0

**MW [kDa]:** 39.6

**pI:** 9.6

**Sequence Coverage [%]:** 15.9

**No. of unique Peptides:** 3

**Heterogeneous nuclear ribonucleoprotein A/B OS=Homo sapiens GN=HNRNPAB PE=**

**1 SV=2**

**Accession:** ROAA_HUMAN **Score:** 120.5

**MW [kDa]:** 36.2

**pI:** 8.8

**Modification(s):** Carbamidomethyl **Sequence Coverage [%]:** 9.3

**No. of unique Peptides:** 2

**Protein 398: DNA-directed RNA polymerase III subunit RPC7 OS=Homo sapiens GN=POLR3G PE=1**

**SV=2**

**Accession:** RPC7_HUMAN **Score:** 68.3

**Database:** SwissProt(SwissProt_2011_08.fasta) **MW [kDa]:** 25.9

**Database Date:** 2011-09-14 **pI:** 4.4

**Sequence Coverage [%]:** 6.7

**No. of unique Peptides:** 0

**Ubiquitin-40S ribosomal protein S27a**

**Accession:** RS27A_HUMAN **Score:** 233.4

**MW [kDa]:** 18.0

**pI:** 10.4

**Sequence Coverage [%]:** 46.2

**No. of unique Peptides:** 2

**40S ribosomal protein S29**

**Accession:** RS29_HUMAN **Score:** 50.8

**MW [kDa]:** 6.7

**pI:** 11.8

**Sequence Coverage [%]:** 14.3

**No. of unique Peptides:** 1

**28S ribosomal protein S22, mitochondrial**

**Accession:** RT22_HUMAN **Score:** 104.2

**MW [kDa]:** 41.3

**pI:** 8.7

**Sequence Coverage [%]:** 10.6

**No. of unique Peptides:** 1

**Reticulon**

**Accession:** RTN3_HUMAN **Score:** 97.8

**MW [kDa]:** 112.5

**pI:** 4.7

**Sequence Coverage [%]:** 3.8

**No. of unique Peptides:** 1

**U2 small nuclear ribonucleoprotein A**

**Accession:** RU2A_HUMAN **Score:** 141.7

**MW [kDa]:** 28.4

**pI:** 9.4

**Sequence Coverage [%]:** 14.5

**No. of unique Peptides:** 2

**Serine/threonine-protein kinase 11-interacting protein**

**Accession:** S11IP_HUMAN **Score:** 58.9

**MW [kDa]:** 121.3

**pI:** 5.2

**Modification(s):** Oxidation **Sequence Coverage [%]:** 1.0

**No. of unique Peptides:** 2

**Ribosome maturation protein SBDS**

**Accession:** SBDS_HUMAN **Score:** 58.3

**MW [kDa]:** 28.7

**pI:** 9.7

**Sequence Coverage [%]:** 6.8

**No. of unique Peptides:** 1

**Septin-11**

**Accession:** SEP11_HUMAN **Score:** 72.8

**MW [kDa]:** 49.4

**pI:** 6.4

**Sequence Coverage [%]:** 6.5

**No. of unique Peptides:** 1

**Septin-2**

**Accession:** SEPT2_HUMAN **Score:** 60.3

**MW [kDa]:** 41.5

**pI:** 6.1

**Sequence Coverage [%]:** 5.3

**No. of unique Peptides:** 2

**SRA stem-loop-interacting RNA-binding protein, mitochondrial**

**Accession:** SLIRP_HUMAN **Score:** 44.0

**MW [kDa]:** 12.3

**pI:** 11.0

**Sequence Coverage [%]:** 9.2

**No. of unique Peptides:** 1

**Mothers against decapentaplegic homolog 6**

**Accession:** SMAD6_HUMAN **Score:** 36.5

**MW [kDa]:** 53.5

**pI:** 9.6

**Sequence Coverage [%]:** 1.8

**No. of unique Peptides:** 1

**Small nuclear ribonucleoprotein Sm D2**

**Accession:** SMD2_HUMAN **Score:** 83.0

**MW [kDa]:** 13.5

**pI:** 10.6

**Modification(s):** Carbamidomethyl **Sequence Coverage [%]:** 16.9

**No. of unique Peptides:** 2

**26S proteasome non-ATPase regulatory subunit 3**

**Accession:** PSMD3_HUMAN **Score:** 103.7

**MW [kDa]:** 60.9

**pI:** 9.0

**Sequence Coverage [%]:** 8.1

**No. of unique Peptides:** 1

**Synaptosomal-associated protein 23 OS=Homo sapiens GN=SNAP23 PE=1 SV=1**

**Accession:** SNP23_HUMAN **Score:** 56.0

**MW [kDa]:** 23.3

**pI:** 4.7

**Sequence Coverage [%]:** 13.7

**No. of unique Peptides:** 1

**U1 small nuclear ribonucleoprotein A**

**Accession:** SNRPA_HUMAN **Score:** 57.8

**MW [kDa]:** 31.3

**pI:** 10.3

**Sequence Coverage [%]:** 3.5

**No. of unique Peptides:** 1

**Sorting nexin-14**

**Accession:** SNX14_HUMAN **Score:** 65.4

**MW [kDa]:** 110.1

**pI:** 6.3

**Sequence Coverage [%]:** 1.5

**No. of unique Peptides:** 1

**Spectrin alpha chain, non-erythrocytic 1**

**Accession:** SPTN1_HUMAN **Score:** 58.4

**MW [kDa]:** 284.4

**pI:** 5.1

**Sequence Coverage [%]:** 0.5

**No. of unique Peptides:** 2

**Sulfide:quinone oxidoreductase, mitochondrial**

**Accession:** SQRD_HUMAN **Score:** 37.3

**MW [kDa]:** 49.9

**pI:** 9.7

**Modification(s):** Oxidation **Sequence Coverage [%]:** 1.6

**No. of unique Peptides:** 1

**Serine/arginine-rich splicing factor 1**

**Accession:** SRSF1_HUMAN **Score:** 120.6

**MW [kDa]:** 27.7

**pI:** 10.8

**Modification(s):** Carbamidomethyl **Sequence Coverage [%]:** 13.7

**No. of unique Peptides:** 2

**Protein 481: Serine/arginine-rich splicing factor 2 OS=Homo sapiens GN=SRSF2 PE=1 SV=4**

**Accession:** SRSF2_HUMAN **Score:** 32.8

**Database:** SwissProt(SwissProt_2011_08.fasta) **MW [kDa]:** 25.5

**Database Date:** 2011-09-14 **pI:** 12.4

**Sequence Coverage [%]:** 3.6

**No. of unique Peptides:** 0

**Translocon-associated protein subunit alpha**

**Accession:** SSRA_HUMAN **Score:** 147.6

**MW [kDa]:** 32.2

**pI:** 4.2

**Sequence Coverage [%]:** 11.9

**No. of unique Peptides:** 3

**Putative protein FAM10A4**

**Accession:** ST134_HUMAN **Score:** 172.8

**MW [kDa]:** 27.4

**pI:** 4.8

**Sequence Coverage [%]:** 15.4

**No. of unique Peptides:** 3

**Serine-threonine kinase receptor-associated protein**

**Accession:** STRAP_HUMAN **Score:** 157.8

**MW [kDa]:** 38.4

**pI:** 4.8

**Sequence Coverage [%]:** 15.4

**No. of unique Peptides:** 2

**Small ubiquitin-related modifier 4**

**Accession:** SUMO4_HUMAN **Score:** 58.0

**MW [kDa]:** 10.7

**pI:** 7.5

**Sequence Coverage [%]:** 12.6

**No. of unique Peptides:** 1

**Lysyl-tRNA synthetase OS=Homo sapiens GN=KARS PE=1 SV=3**

**Accession:** SYK_HUMAN **Score:** 41.1

**MW [kDa]:** 68.0

**pI:** 5.9

**Sequence Coverage [%]:** 1.8

**No. of unique Peptides:** 1

**Activated RNA polymerase II transcriptional coactivator p15**

**Accession:** TCP4_HUMAN **Score:** 102.5

**MW [kDa]:** 14.4

**pI:** 10.1

**Sequence Coverage [%]:** 20.5

**No. of unique Peptides:** 1

**T-complex protein 1 subunit epsilon**

**Accession:** TCPE_HUMAN **Score:** 404.2

**MW [kDa]:** 59.6

**pI:** 5.3

**Sequence Coverage [%]:** 18.5

**No. of unique Peptides:** 5

**T-complex protein 1 subunit gamma**

**Accession:** TCPG_HUMAN **Score:** 676.7

**MW [kDa]:** 60.5

**pI:** 6.1

**Modification(s):** Carbamidomethyl **Sequence Coverage [%]:** 31.2

**No. of unique Peptides:** 8

**T-complex protein 1 subunit theta OS=Homo sapiens GN=CCT8 PE=1 SV=4**

**Accession:** TCPQ_HUMAN **Score:** 1408.0

**MW [kDa]:** 59.6

**pI:** 5.3

**Modification(s):** Carbamidomethyl, Oxidation **Sequence Coverage [%]:** 43.2

**No. of unique Peptides:** 21

**Transcription factor A, mitochondrial**

**Accession:** TFAM_HUMAN **Score:** 115.6

**Database:** SwissProt(SwissProt_56.1.fasta) **MW [kDa]:** 29.1

**Database Date:** 2008-10-22 **pI:** 10.3

**Sequence Coverage [%]:** 13.8

**No. of unique Peptides:** 1

**Transforming growth factor beta-3**

**Accession:** TGFB3_HUMAN **Score:** 32.1

**MW [kDa]:** 47.3

**pI:** 9.3

**Sequence Coverage [%]:** 2.7

**No. of unique Peptides:** 1

**Acetyl-CoA acetyltransferase, cytosolic**

**Accession:** THIC_HUMAN **Score:** 63.1

**MW [kDa]:** 41.3

**pI:** 6.5

**Sequence Coverage [%]:** 8.1

**No. of unique Peptides:** 1

**Acetyl-CoA acetyltransferase, mitochondrial**

**Accession:** THIL_HUMAN **Score:** 153.1

**MW [kDa]:** 45.2

**pI:** 9.6

**Sequence Coverage [%]:** 10.1

**No. of unique Peptides:** 2

**3-ketoacyl-CoA thiolase, mitochondrial**

**Accession:** THIM_HUMAN **Score:** 43.6

**MW [kDa]:** 41.9

**pI:** 9.3

**Sequence Coverage [%]:** 3.3

**No. of unique Peptides:** 1

**Thioredoxin**

**Accession:** THIO_HUMAN **Score:** 71.1

**MW [kDa]:** 11.7

**pI:** 4.7

**Sequence Coverage [%]:** 12.4

**No. of unique Peptides:** 1

**Mitochondrial import inner membrane translocase subunit TIM50**

**Accession:** TIM50_HUMAN **Score:** 321.5

**MW [kDa]:** 39.6

**pI:** 9.3

**Sequence Coverage [%]:** 24.6

**No. of unique Peptides:** 6

**Transmembrane protein 33**

**Accession:** TMM33_HUMAN **Score:** 156.0

**MW [kDa]:** 28.0

**pI:** 10.6

**Sequence Coverage [%]:** 11.7

**No. of unique Peptides:** 2

**Protein 402: Thioredoxin-related transmembrane protein 1 OS=Homo sapiens GN=TMX1 PE=1 SV=1**

**Accession:** TMX1_HUMAN **Score:** 67.7

**Database:** SwissProt(SwissProt_2011_08.fasta) **MW [kDa]:** 31.8

**Database Date:** 2011-09-14 **pI:** 4.8

**Sequence Coverage [%]:** 14.3

**No. of unique Peptides:** 0

**Mitochondrial import receptor subunit TOM40**

**Accession:** TOM40_HUMAN **Score:** 249.4

**MW [kDa]:** 37.9

**pI:** 7.0

**Sequence Coverage [%]:** 15.8

**No. of unique Peptides:** 5

**Triosephosphate isomerase**

**Accession:** TPIS_HUMAN **Score:** 329.7

**MW [kDa]:** 30.8

**pI:** 5.6

**Modification(s):** Carbamidomethyl **Sequence Coverage [%]:** 29.0

**No. of unique Peptides:** 4

**Protein 382: TRM112-like protein OS=Homo sapiens GN=AD-001 PE=1 SV=1**

**Accession:** TR112_HUMAN **Score:** 73.9

**Database:** SwissProt(SwissProt_56.1.fasta) **MW [kDa]:** 14.2

**Database Date:** 2008-10-22 **pI:** 5.1

**Sequence Coverage [%]:** 28.8

**No. of unique Peptides:** 0

**Protein 409: Probable tRNA (uracil-O(2)-)-methyltransferase OS=Homo sapiens GN=METTL19 PE=2**

**SV=2**

**Accession:** TRM44_HUMAN **Score:** 65.0

**Database:** SwissProt(SwissProt_2011_08.fasta) **MW [kDa]:** 84.6

**Database Date:** 2011-09-14 **pI:** 7.2

**Sequence Coverage [%]:** 5.0

**No. of unique Peptides:** 0

**Thymidylate synthase**

**Accession:** TYSY_HUMAN **Score:** 56.0

**MW [kDa]:** 35.7

**pI:** 6.6

**Sequence Coverage [%]:** 7.0

**No. of unique Peptides:** 1

**UDP-N-acetylhexosamine pyrophosphorylase**

**Accession:** UAP1_HUMAN **Score:** 92.4

**MW [kDa]:** 58.7

**pI:** 5.9

**Sequence Coverage [%]:** 5.7

**No. of unique Peptides:** 1

**Ubiquitin-conjugating enzyme E2 variant 2**

**Accession:** UB2V2_HUMAN **Score:** 36.8

**MW [kDa]:** 16.4

**pI:** 9.0

**Sequence Coverage [%]:** 6.9

**No. of unique Peptides:** 1

**Cytochrome b-c1 complex subunit Rieske, mitochondrial**

**Accession:** UCRI_HUMAN **Score:** 143.8

**MW [kDa]:** 29.6

**pI:** 9.4

**Sequence Coverage [%]:** 18.6

**No. of unique Peptides:** 2

**Protein 236: Utrophin OS=Homo sapiens GN=UTRN PE=1 SV=2**

**Accession:** UTRO_HUMAN **Score:** 154.2

**Database:** SwissProt(SwissProt_57.15.fasta) **MW [kDa]:** 394.2

**Database Date:** 2011-05-23 **pI:** 5.1

**Sequence Coverage [%]:** 2.3

**No. of unique Peptides:** 0

**Vesicle-associated membrane protein 3**

**Accession:** VAMP3_HUMAN **Score:** 41.7

**MW [kDa]:** 11.3

**pI:** 9.7

**Sequence Coverage [%]:** 17.0

**No. of unique Peptides:** 1

**Vesicle-associated membrane protein-associated protein A**

**Accession:** VAPA_HUMAN **Score:** 127.3

**MW [kDa]:** 27.9

**pI:** 9.6

**Sequence Coverage [%]:** 11.2

**No. of unique Peptides:** 2

**Synaptic vesicle membrane protein VAT-1 homolog-like**

**Accession:** VAT1L_HUMAN **Score:** 54.1

**MW [kDa]:** 45.9

**pI:** 4.8

**Sequence Coverage [%]:** 4.8

**No. of unique Peptides:** 0

**Voltage-dependent anion-selective channel protein 1**

**Accession:** VDAC1_HUMAN **Score:** 446.8

**MW [kDa]:** 30.8

**pI:** 9.2

**Modification(s):** Carbamidomethyl **Sequence Coverage [%]:** 35.0

**No. of unique Peptides:** 6

**Voltage-dependent anion-selective channel protein 3**

**Accession:** VDAC3_HUMAN **Score:** 241.3

**MW [kDa]:** 30.6

**pI:** 9.5

**Sequence Coverage [%]:** 19.1

**No. of unique Peptides:** 3

**Vacuolar protein sorting-associated protein 13A**

**Accession:** VP13A_HUMAN **Score:** 58.6

**MW [kDa]:** 360.0

**pI:** 5.9

**Sequence Coverage [%]:** 0.5

**No. of unique Peptides:** 2

**Vacuolar protein sorting-associated protein VTA1 homolog**

**Accession:** VTA1_HUMAN **Score:** 36.1

**MW [kDa]:** 33.9

**pI:** 5.8

**Sequence Coverage [%]:** 3.3

**No. of unique Peptides:** 1

**WD repeat-containing protein 62**

**Accession:** WDR62_HUMAN **Score:** 97.8

) **MW [kDa]:** 165.8

**pI:** 5.5

**Sequence Coverage [%]:** 3.2

**No. of unique Peptides:** 1
