# Supplementary material for: Affinity purification-mass spectrometry analysis of bcl-2 interactome identified SLIRP as a novel interacting protein
Source: Cell Death Dis. 2016 Feb 11;7(2):e2090–. doi: 10.1038/cddis.2015.357 (PMC4849145; doi:10.1038/cddis.2015.357)

1B57


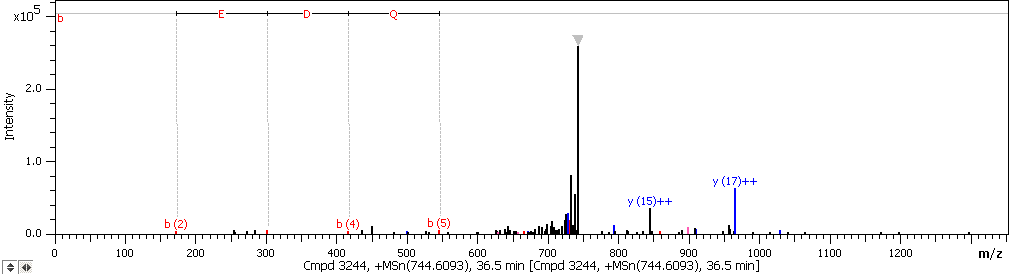


AIMP1


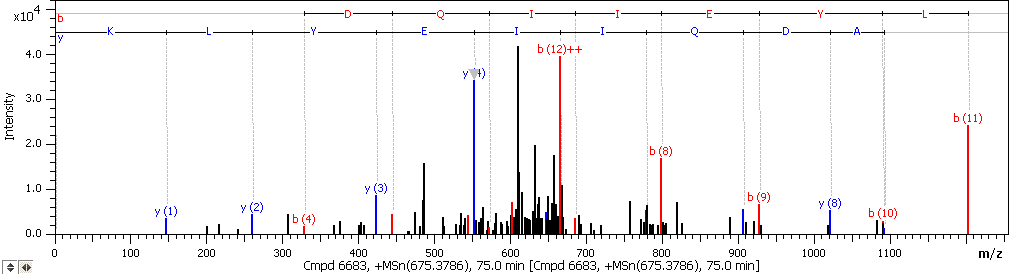


ANX11


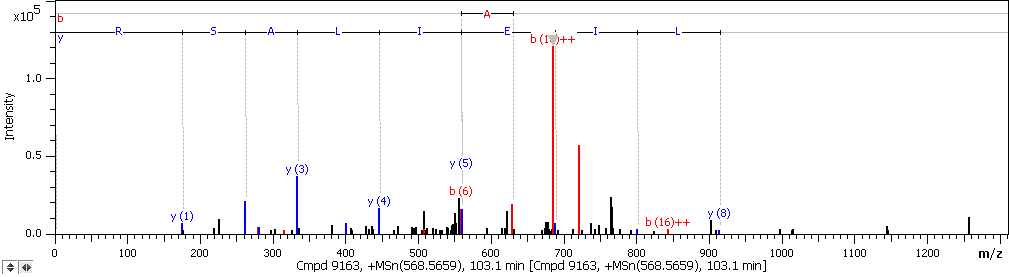


ANXA4


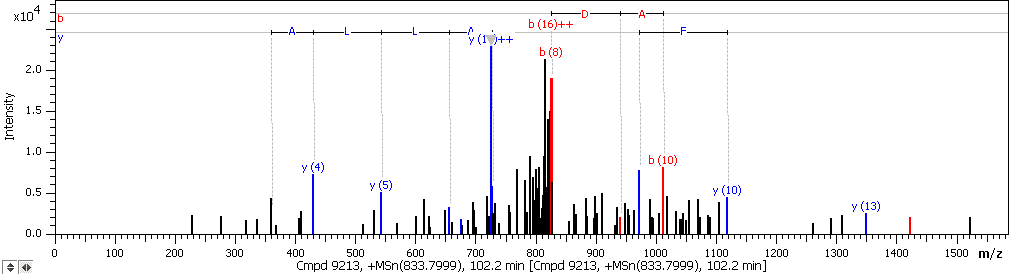


ARP3


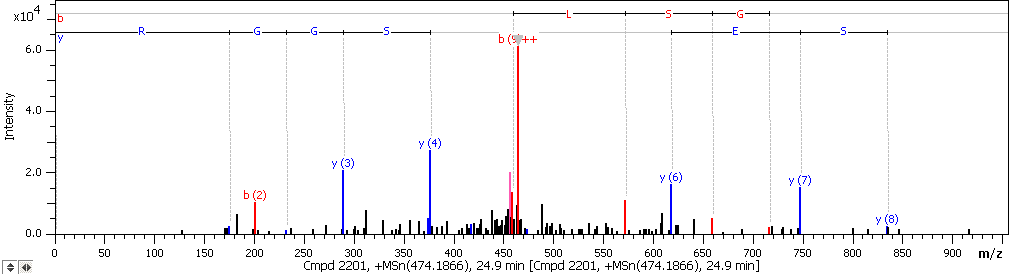


AT1B3


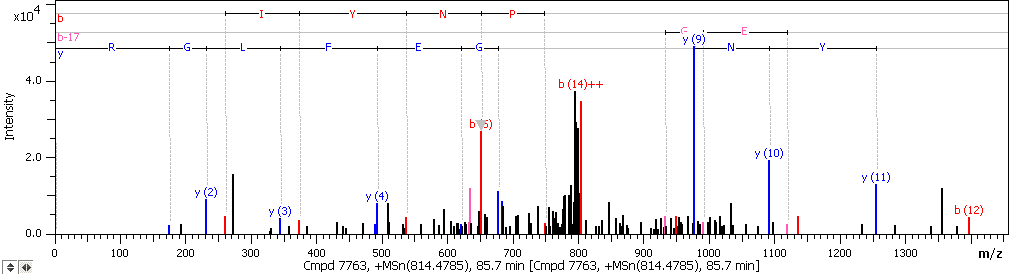


BAG2


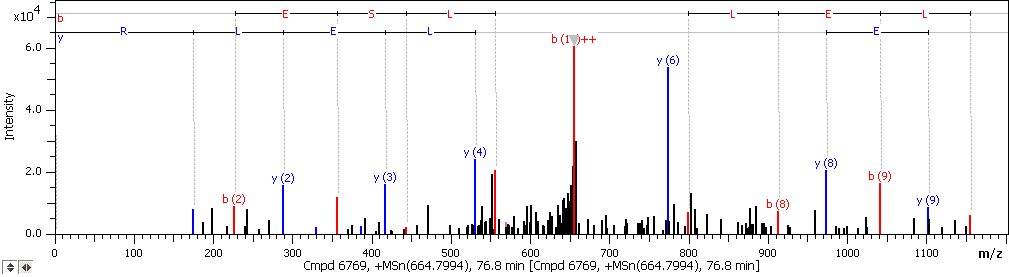


CALL5


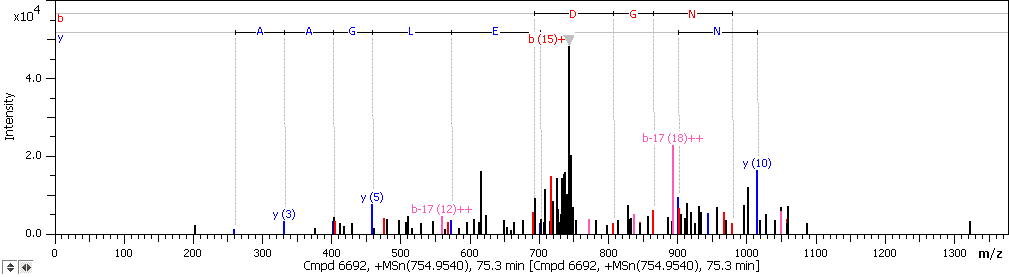


CAP1


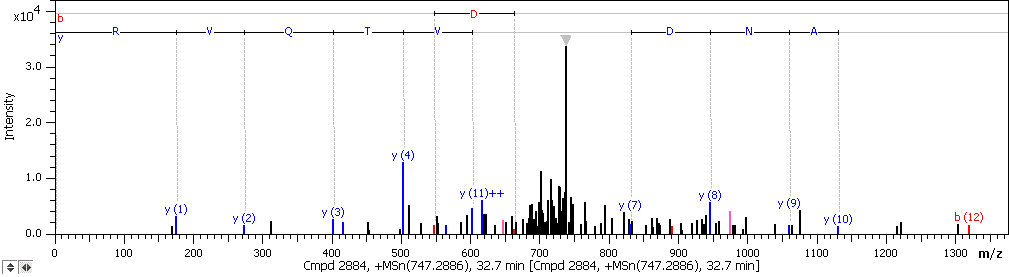


CENPF


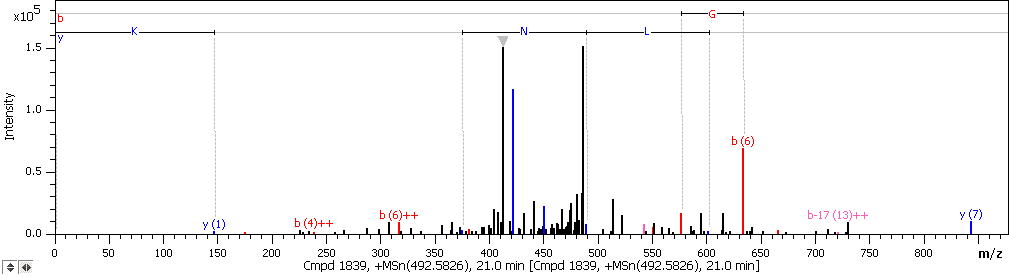


CHDR1


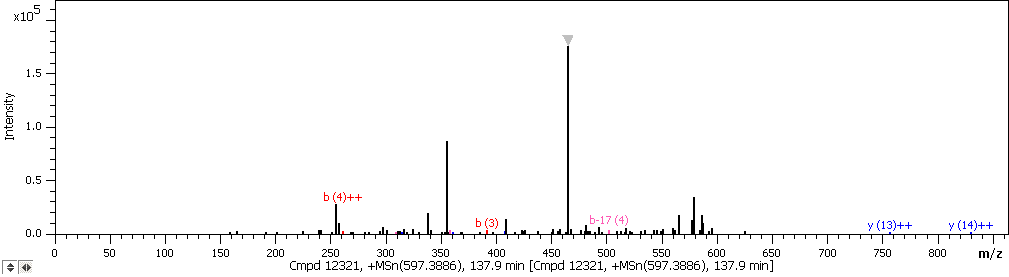


CLK3


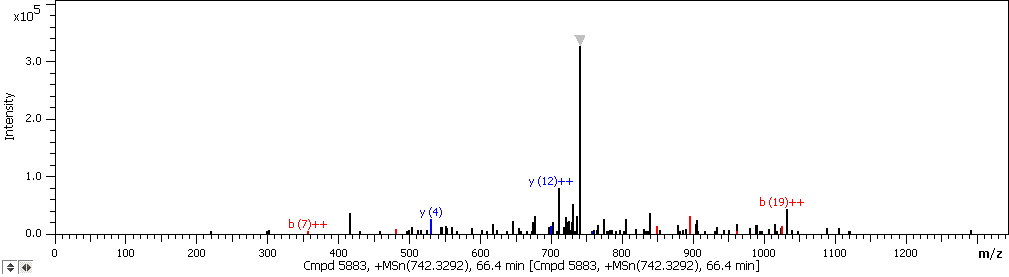


COTL1


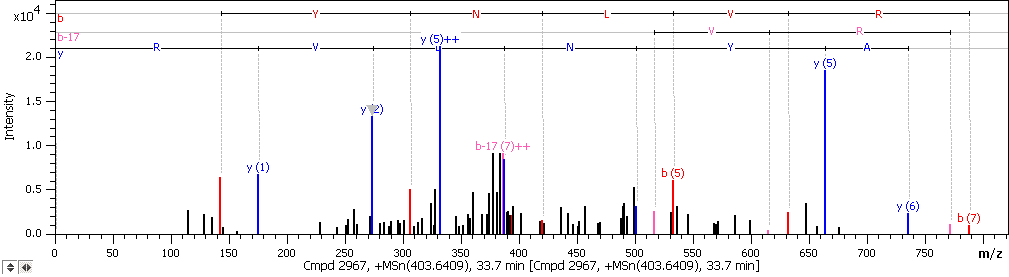


COX6C

CUX2


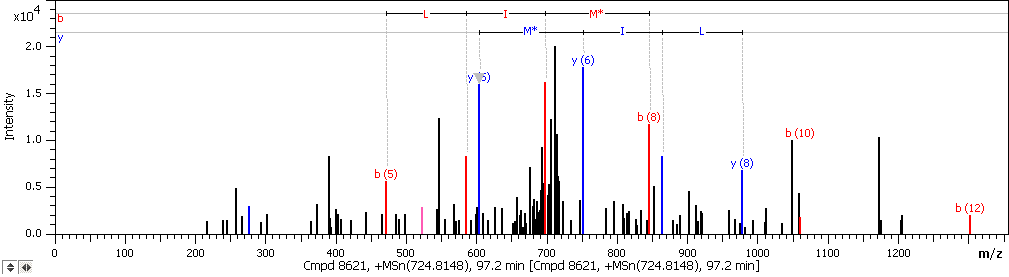


DBLOH


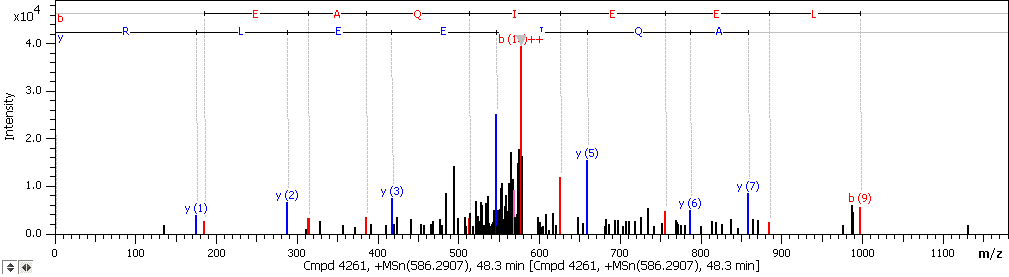


DEND3


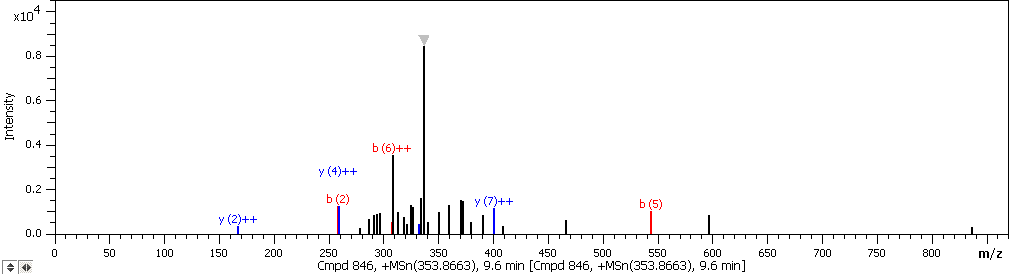


DEST


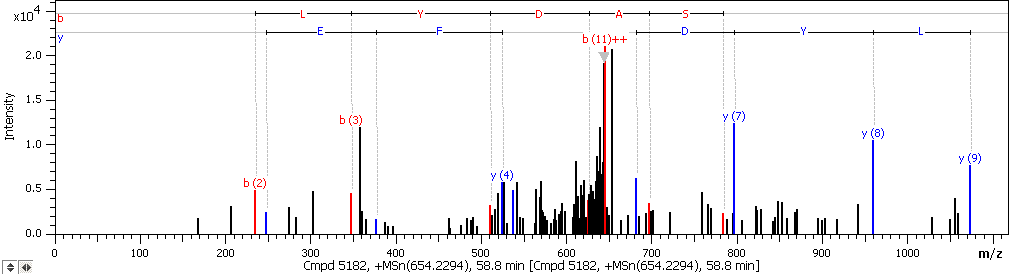


DHSA

DOP 2


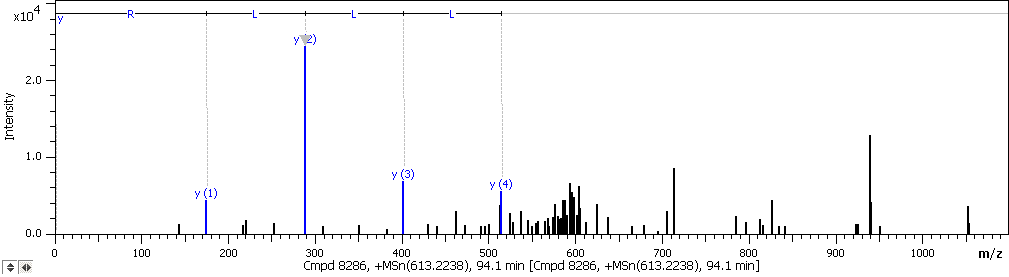


DPM1


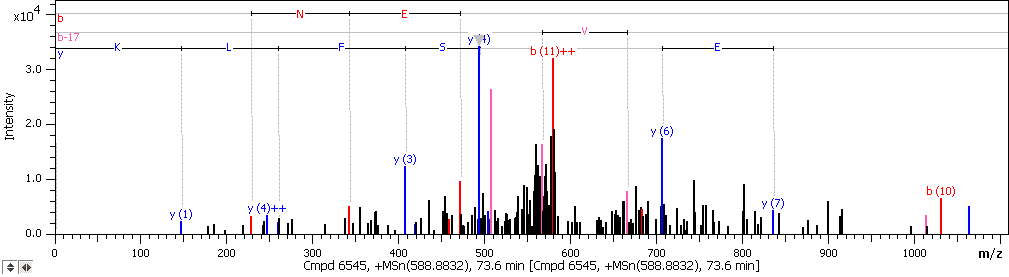


DX39B


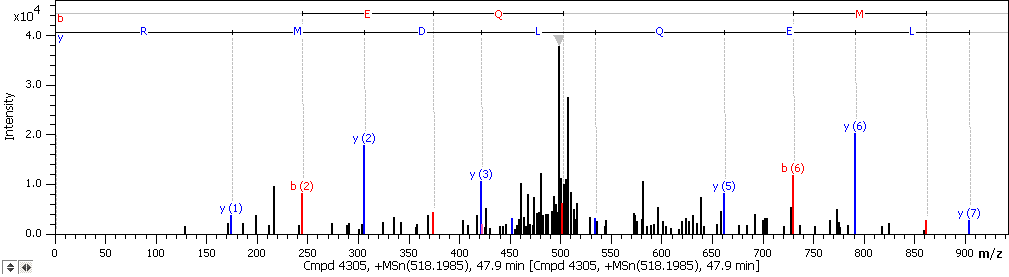


EIF3F


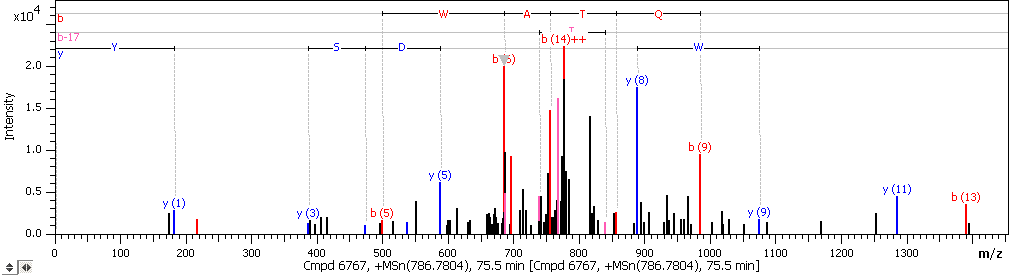


EIF3M


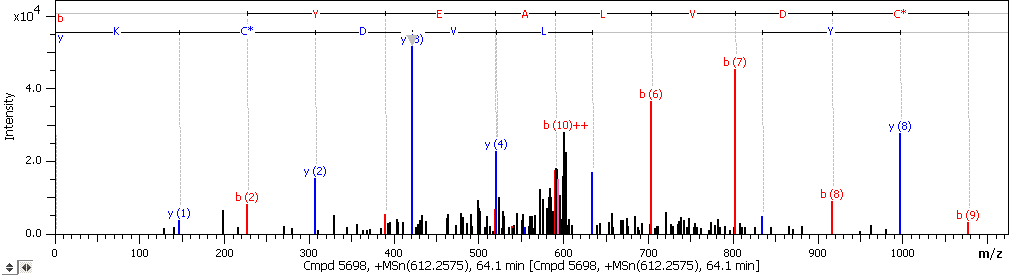


ERF1


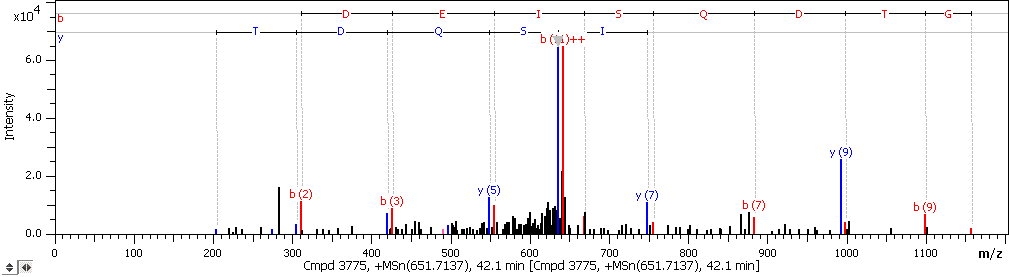


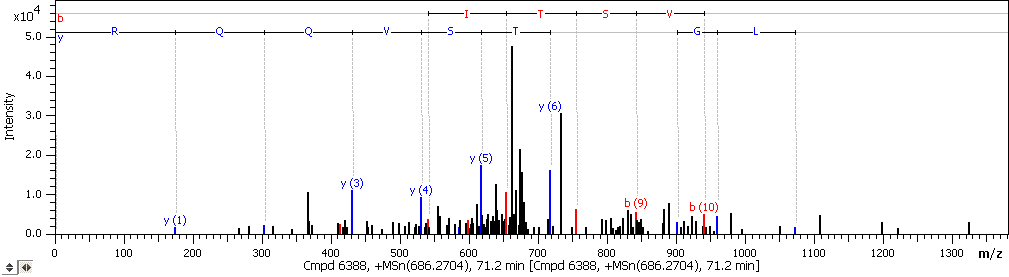


FA49B


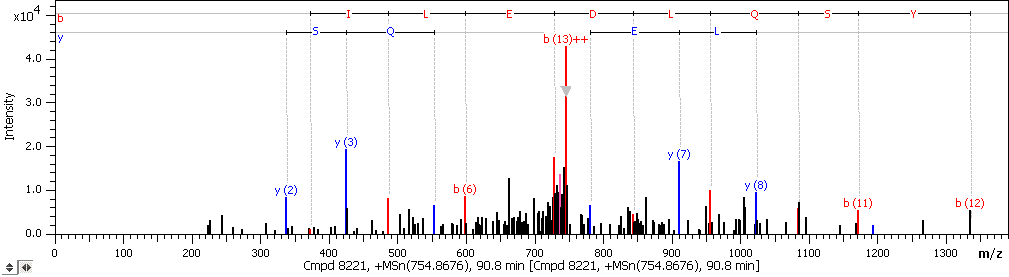


FHL1


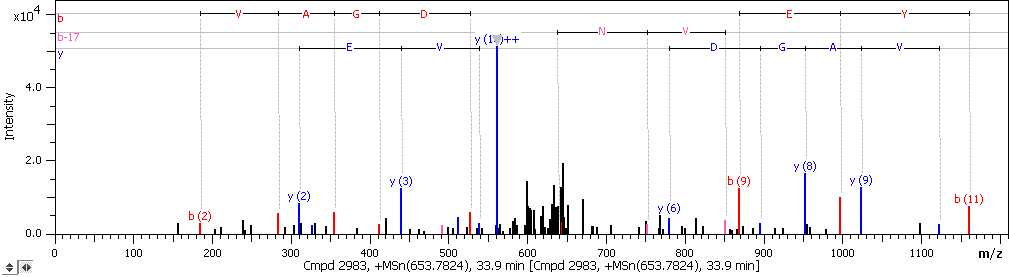


GDIB


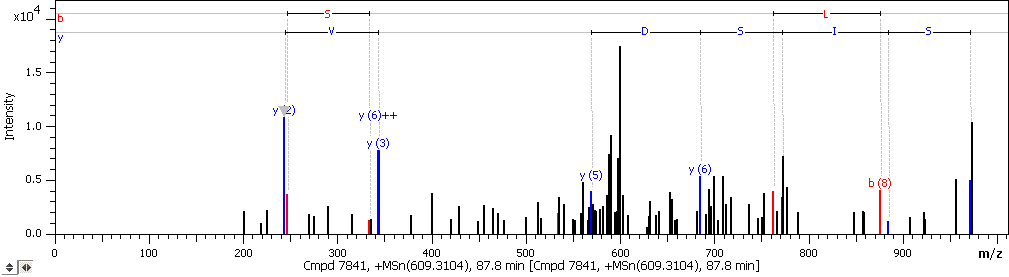


GFPT1


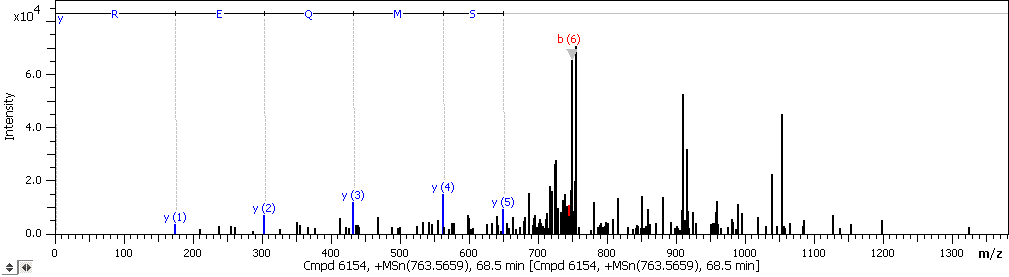


GLYM


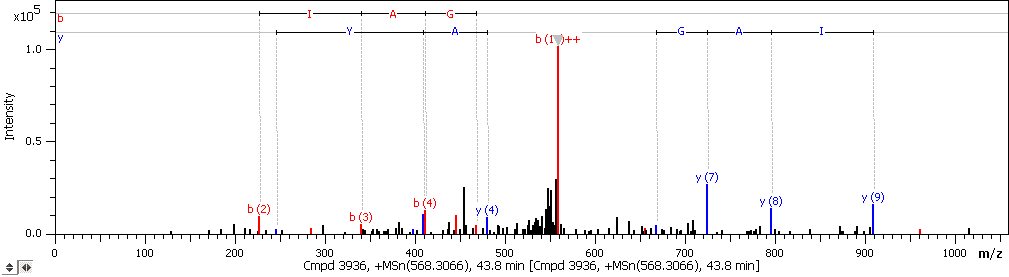


GRSF1


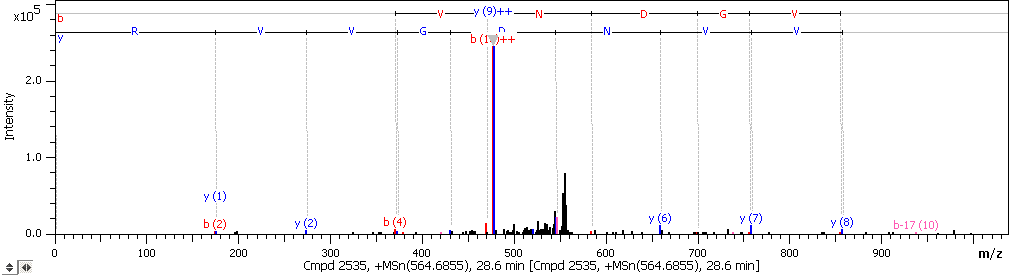


HAUS5

HXA6


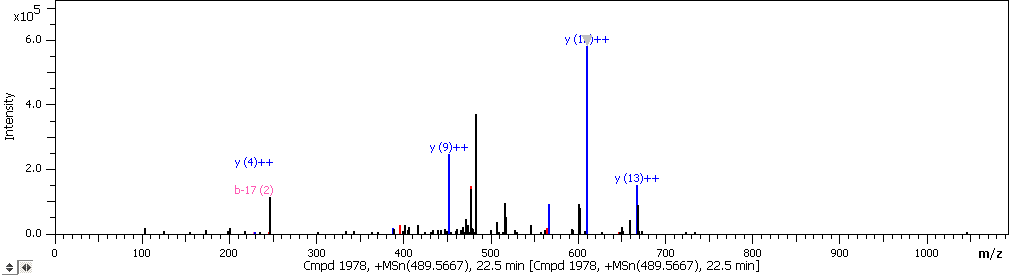


IDH3A


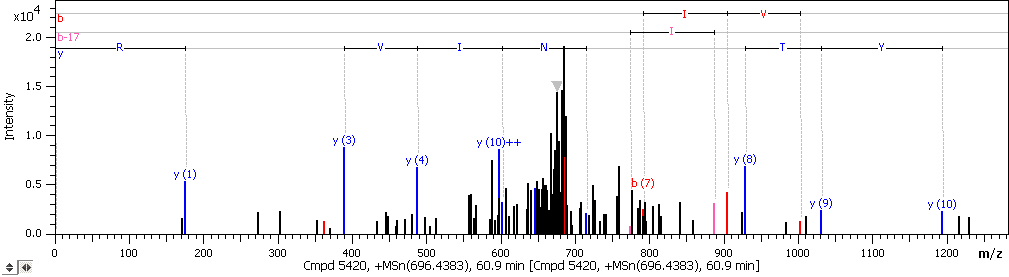


IF4E


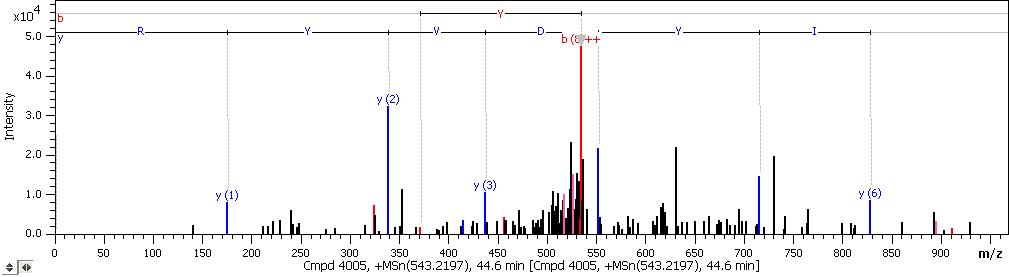


IF5A1


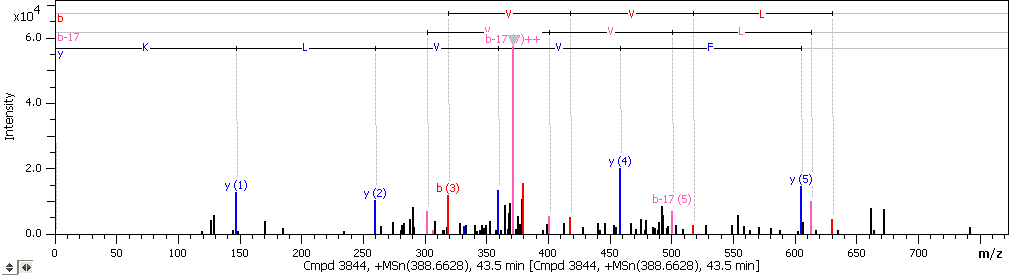


ILF2


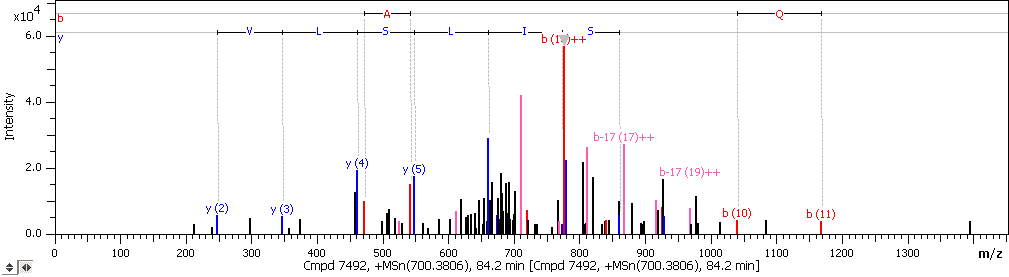


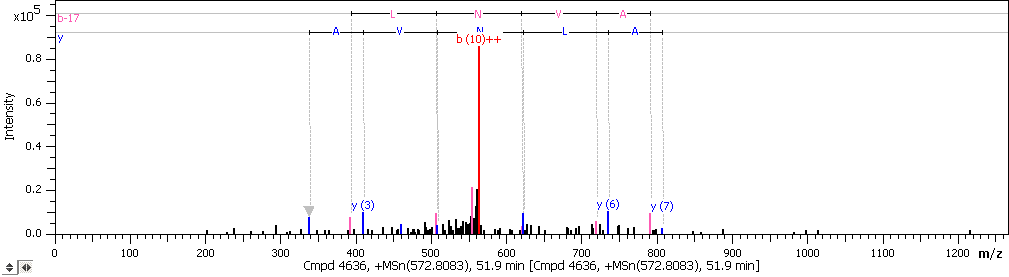


JPH3


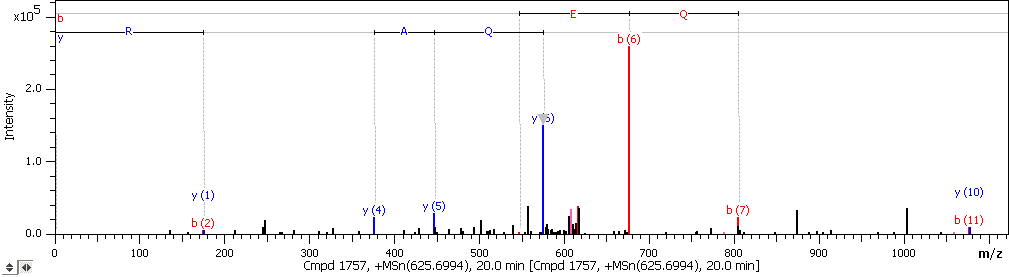


KCRB


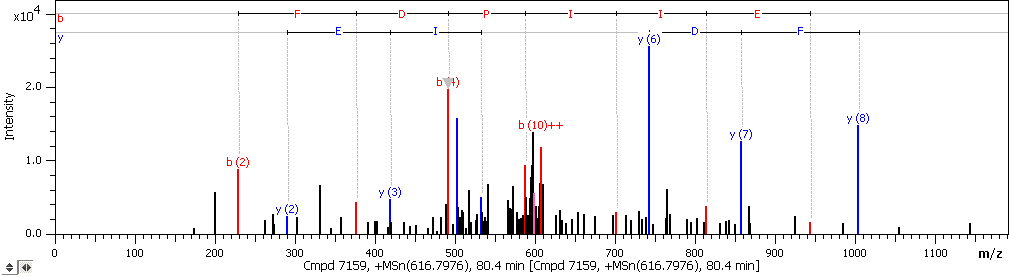


LIS1


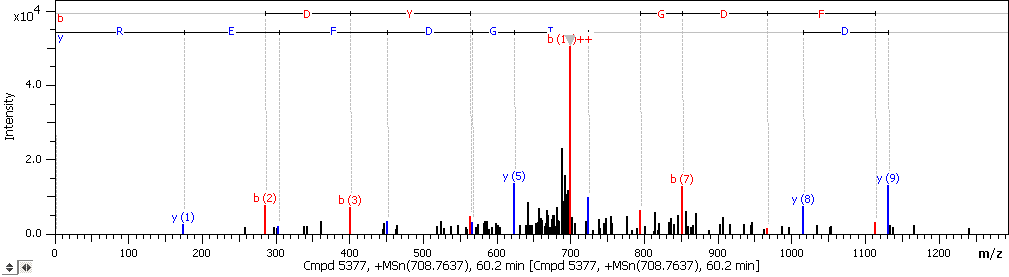


LRC8E


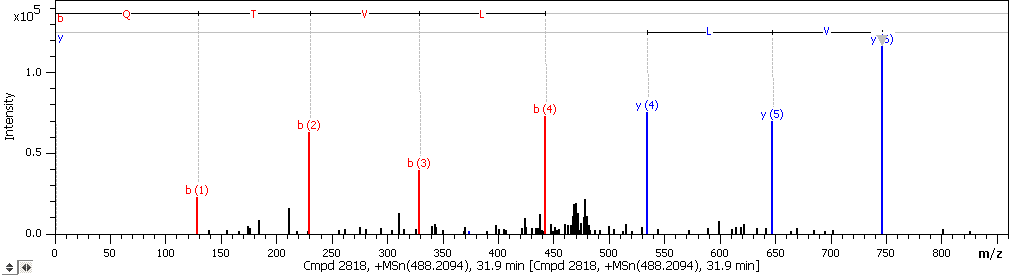


LSM12


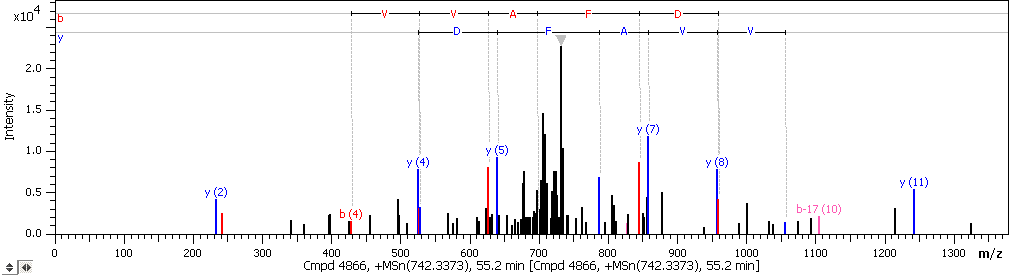


MGST3


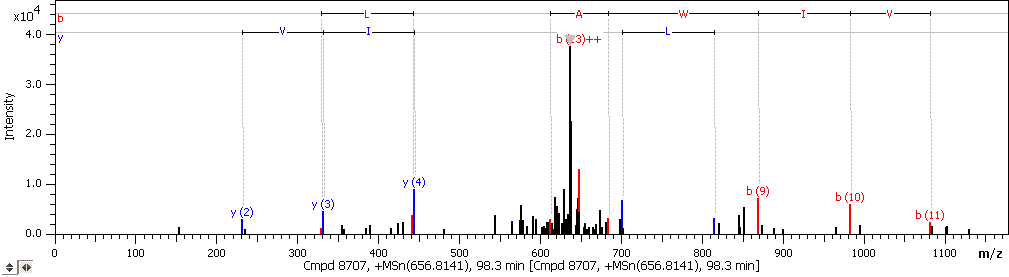


MIF


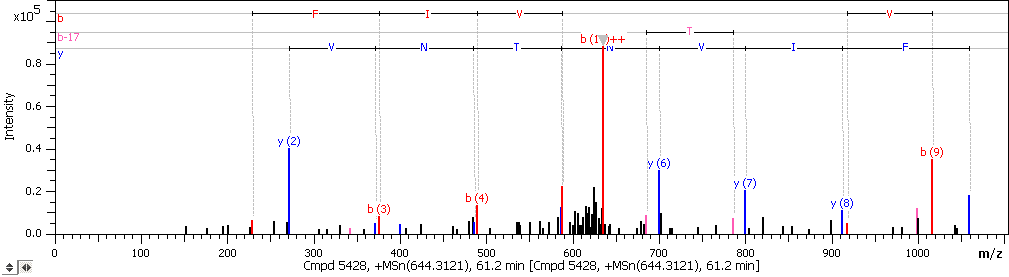


MOB1A


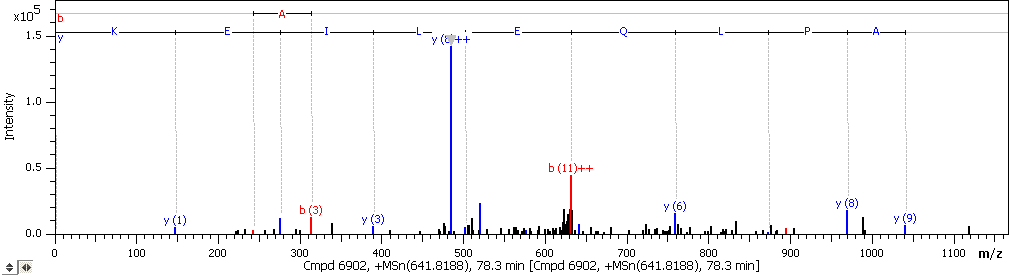


NALD2


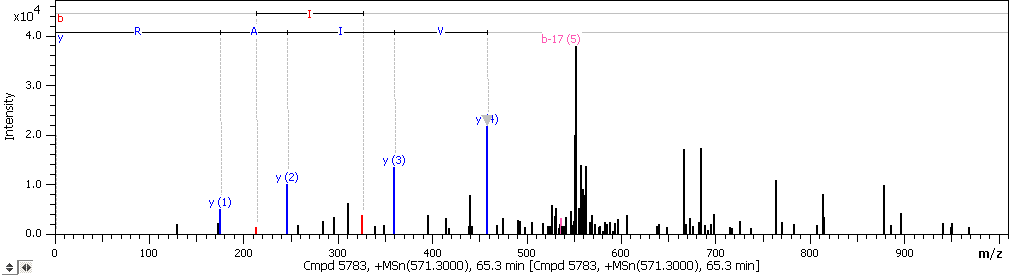


NDUS8


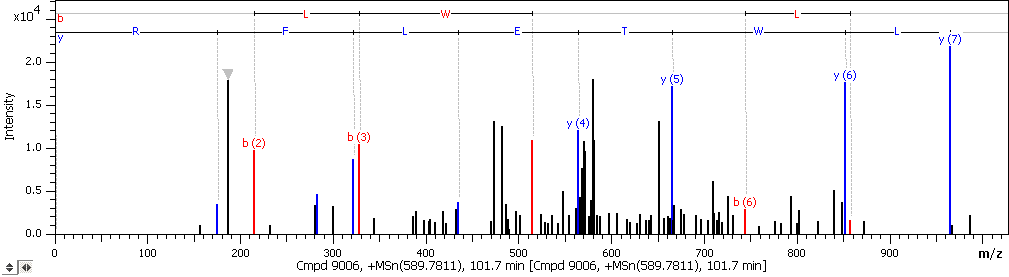


NFX1


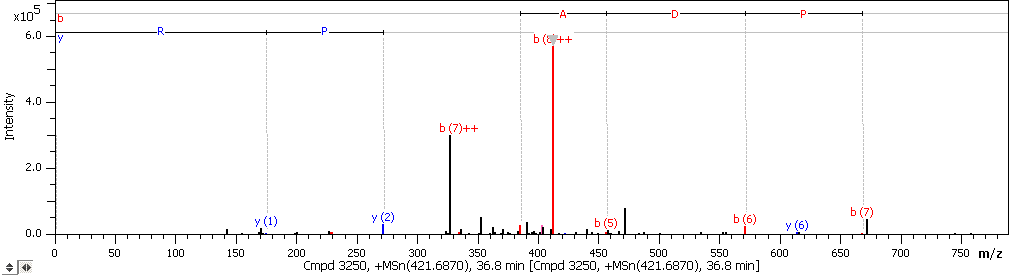


OLA1


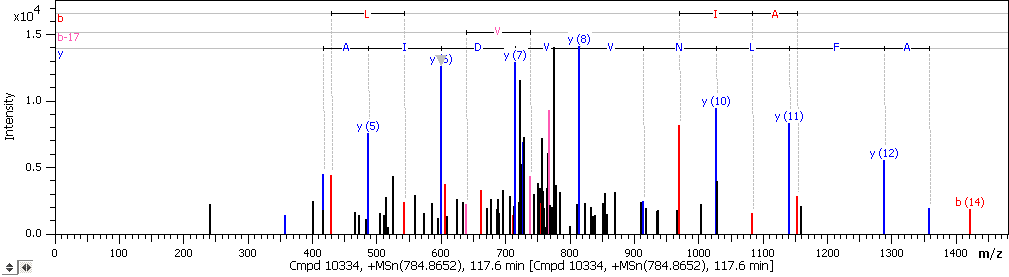


PDCD6


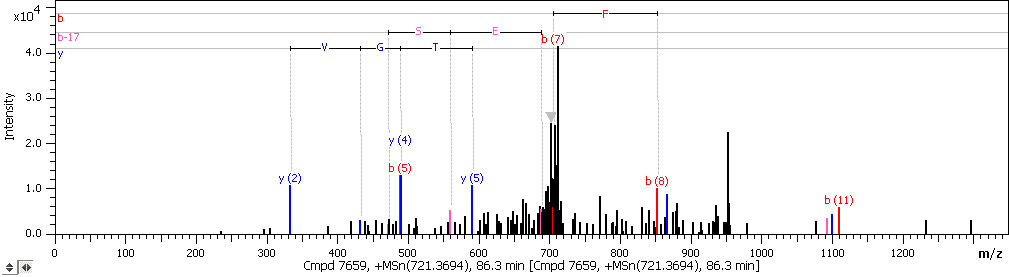


PRS10


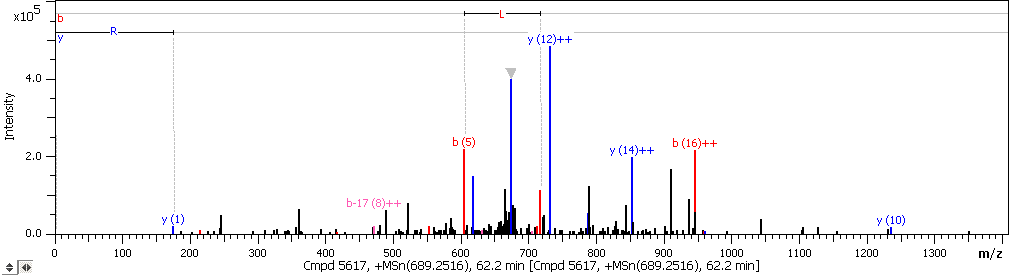


PRS4


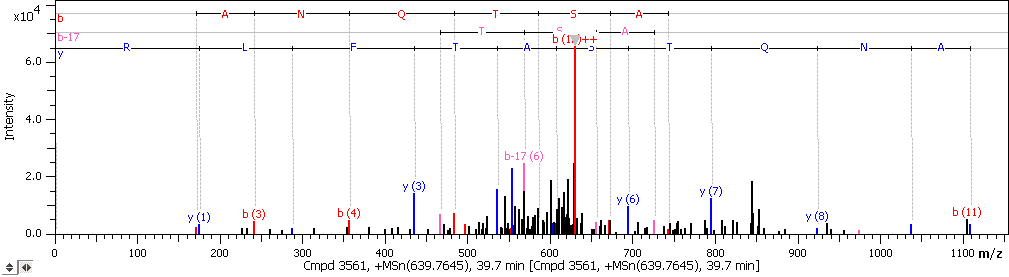


PSA1


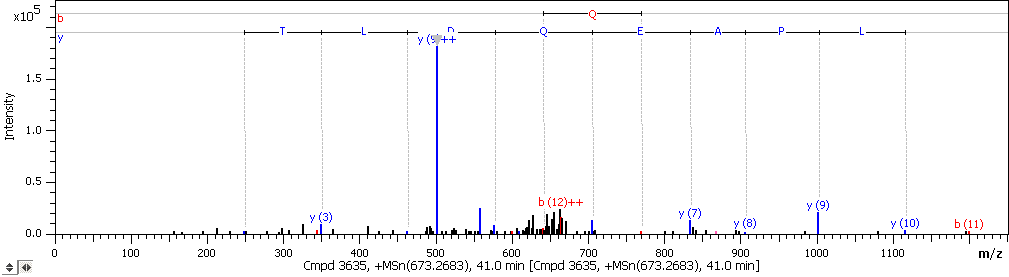


PSA2


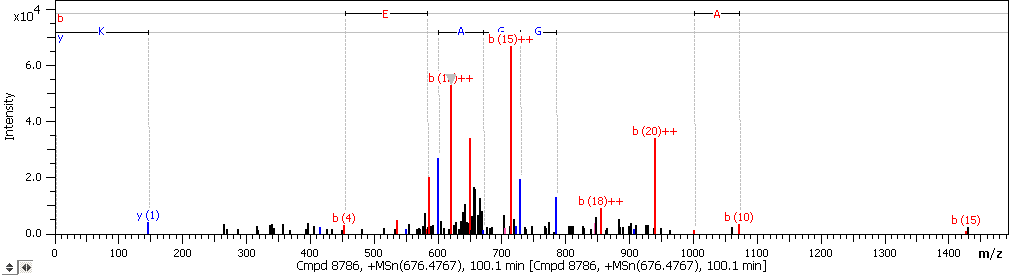


PSA4


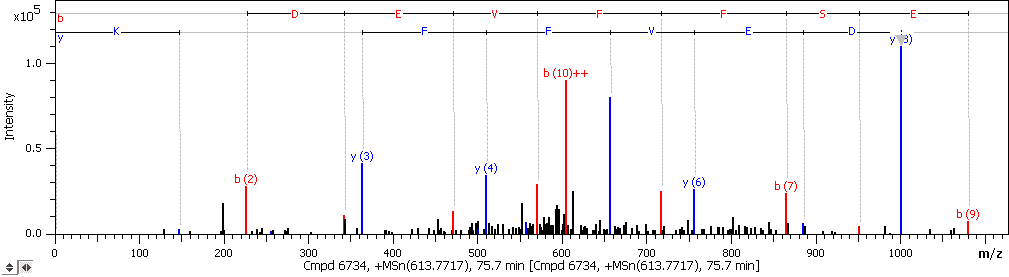


PSB5


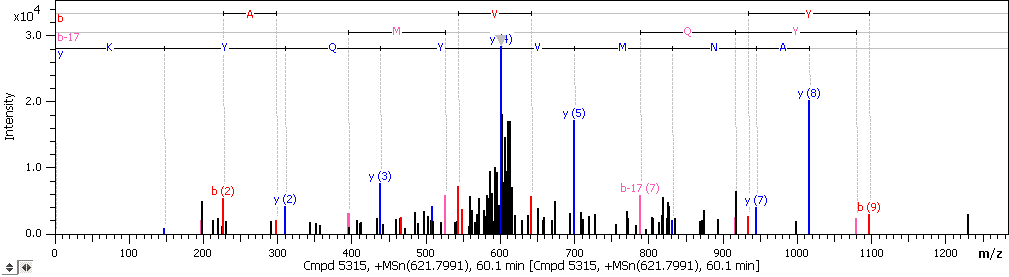


PSMD3


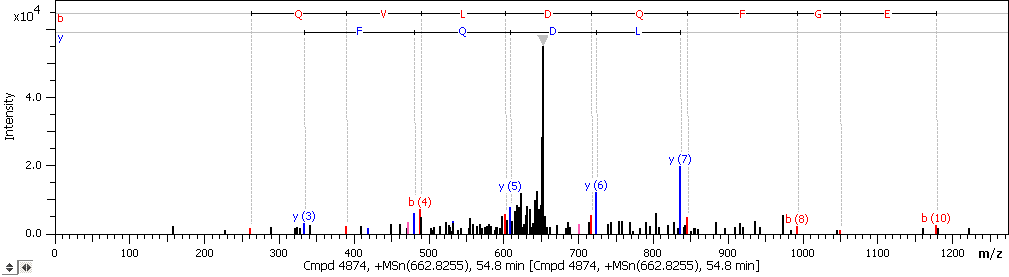


PSME1


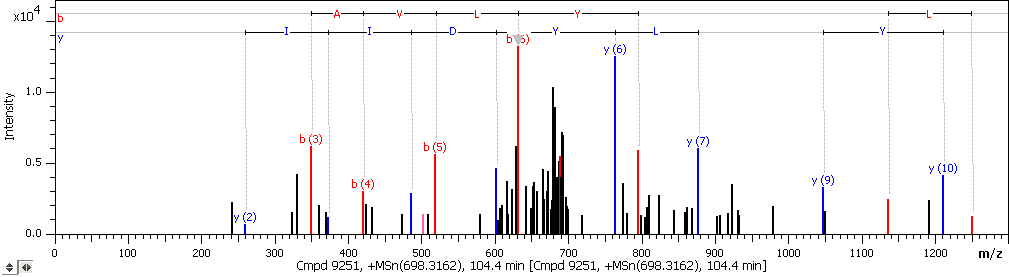


QCR7


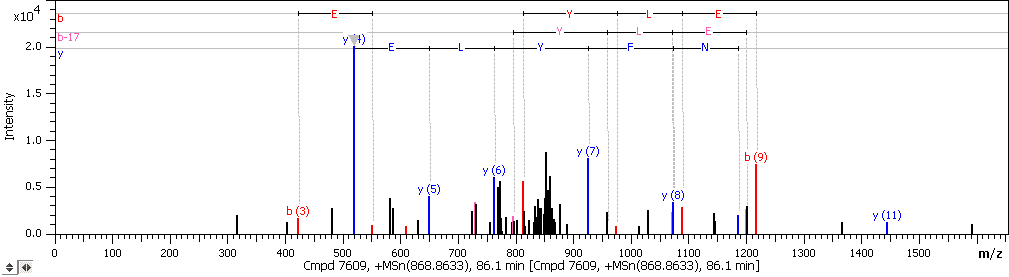


RAB21


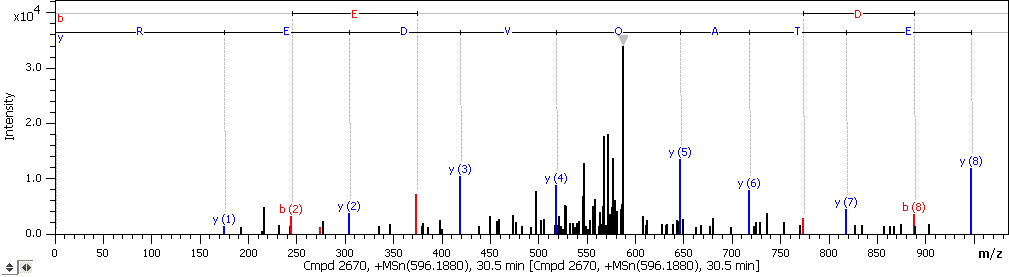


RA1L2


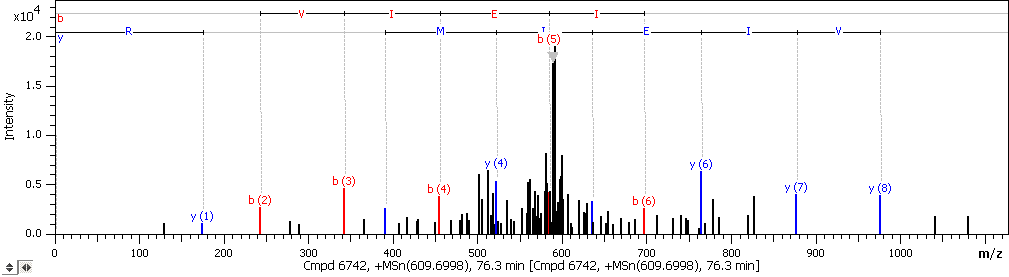


RAE1


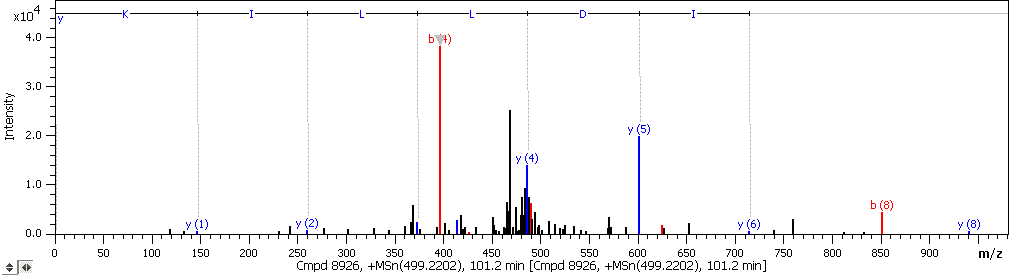


RAP2B


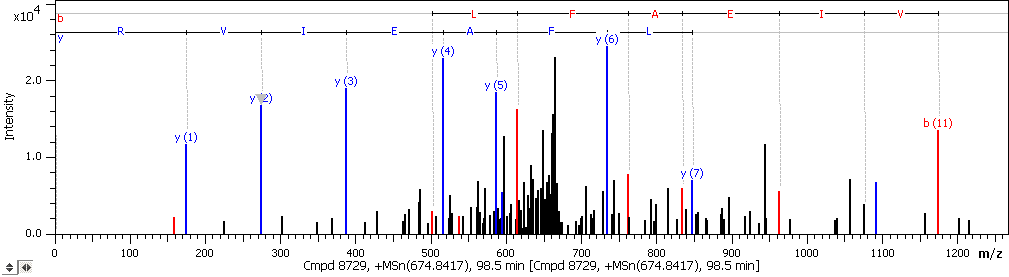


RASN


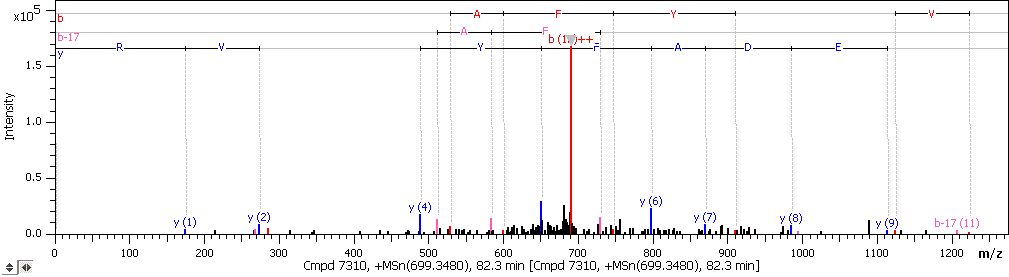


RL21


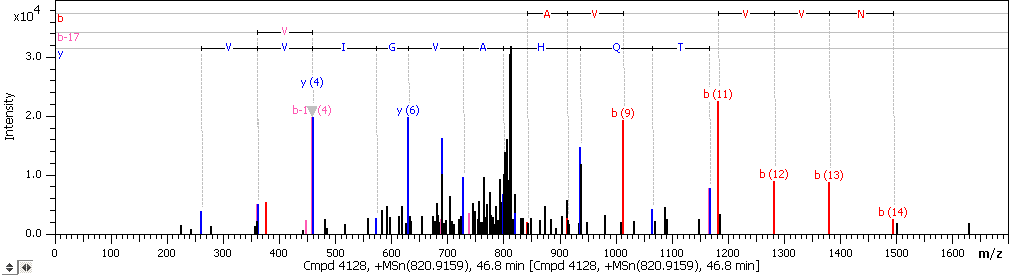


RL38


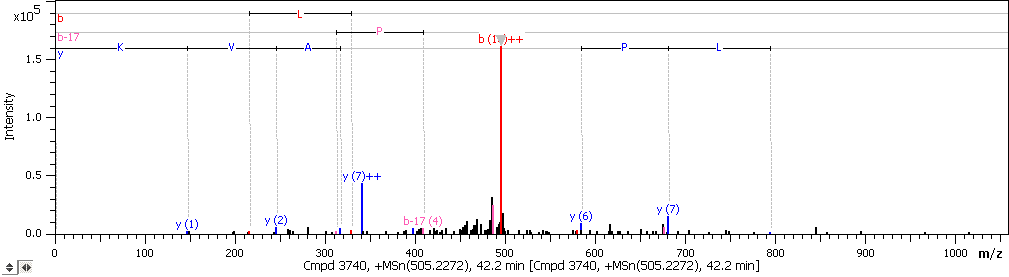


RM01


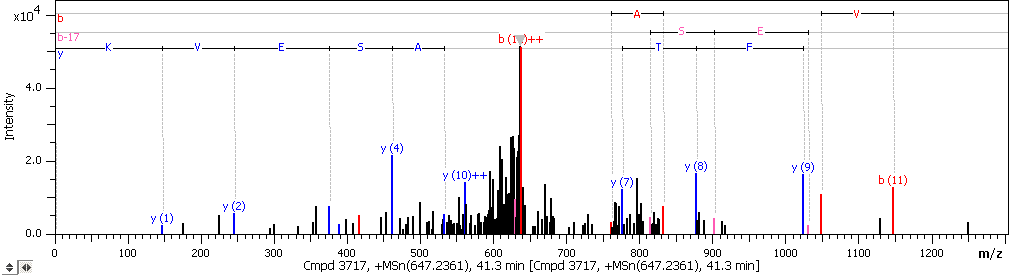


RMD1


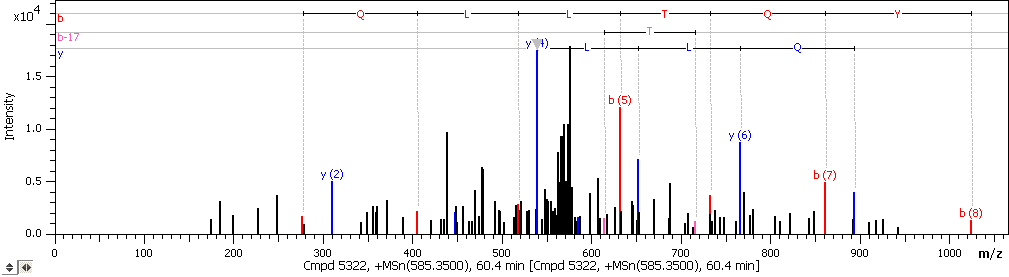


ROA0


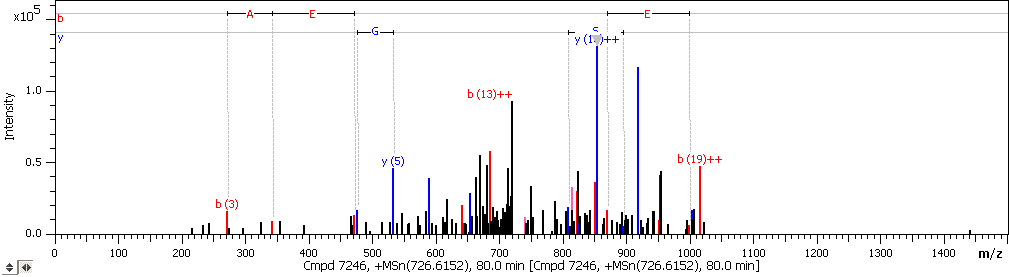


RS29


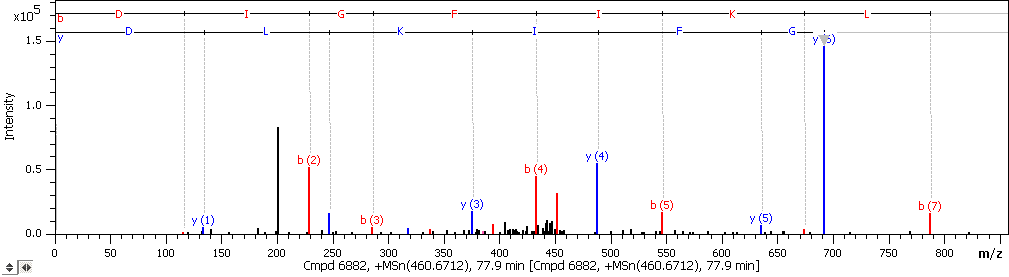


RT22


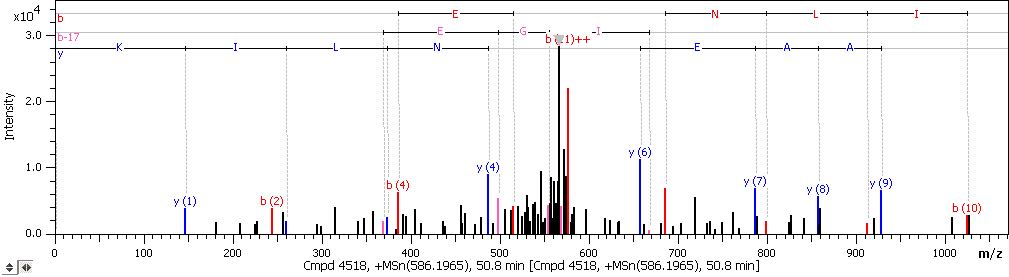


RTN3


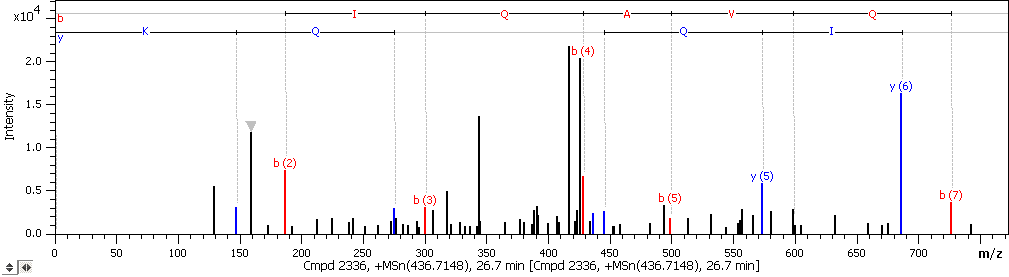


SBDS


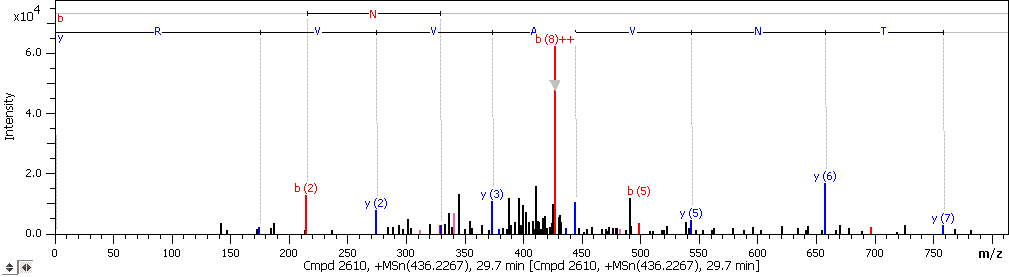


SEP11


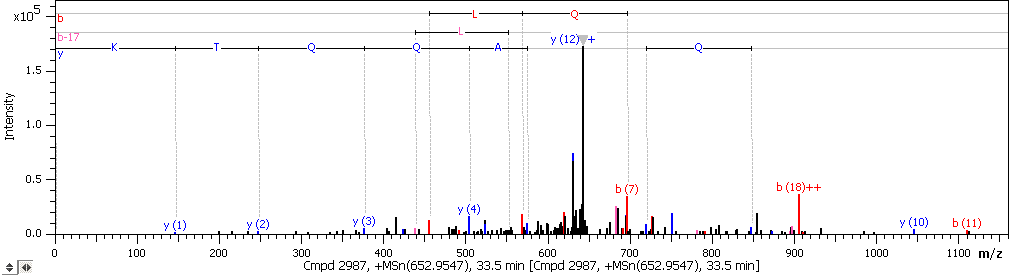


SLIRP


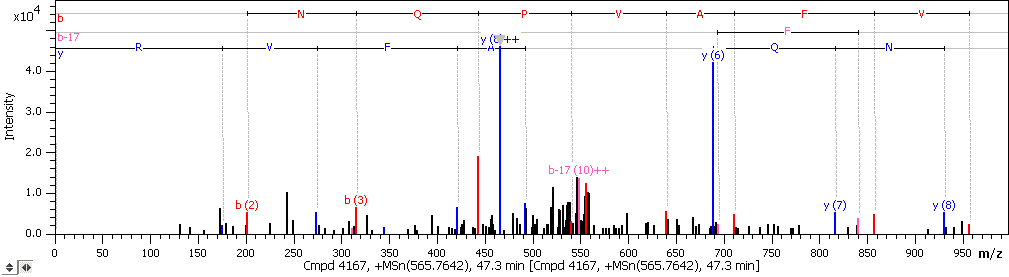


SMAD6


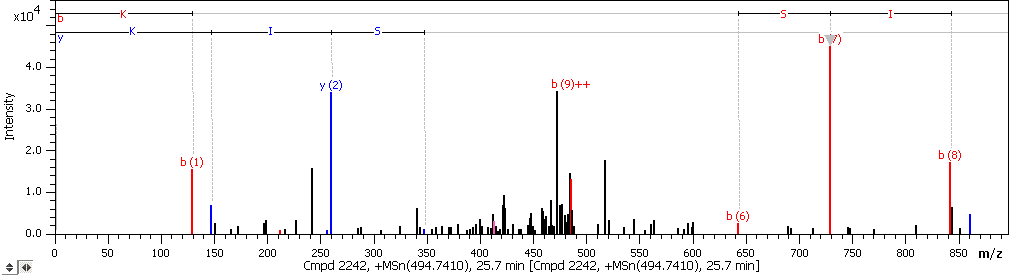


SMD3


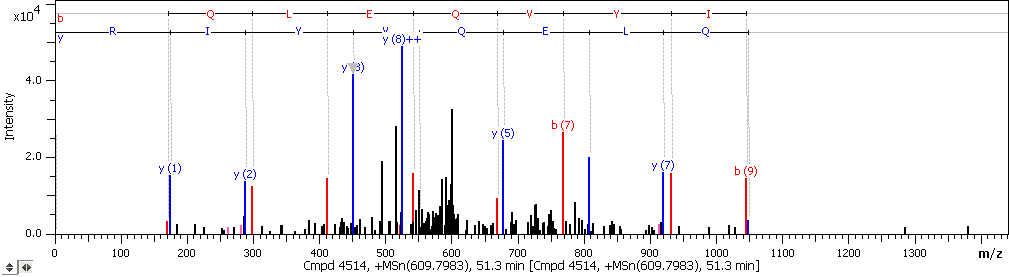


SNP23


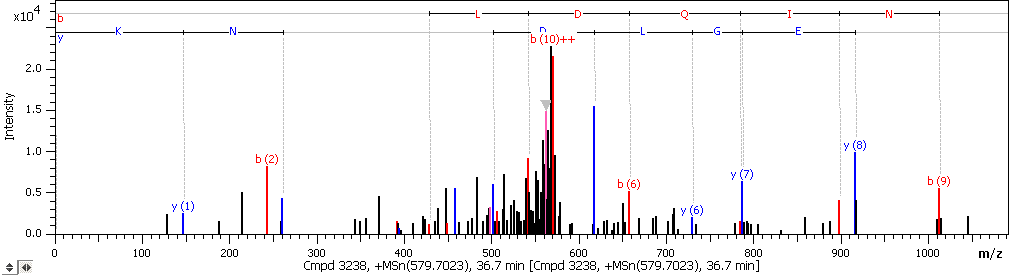


SNRPA


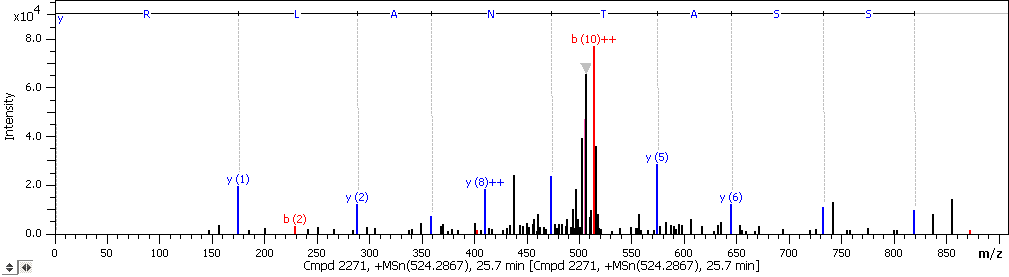


SNX14


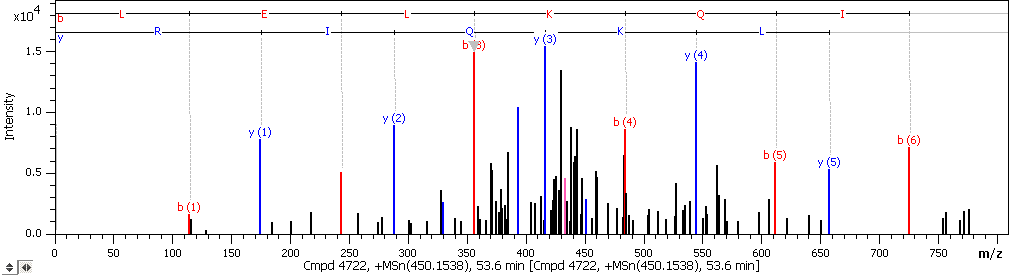


SQRD


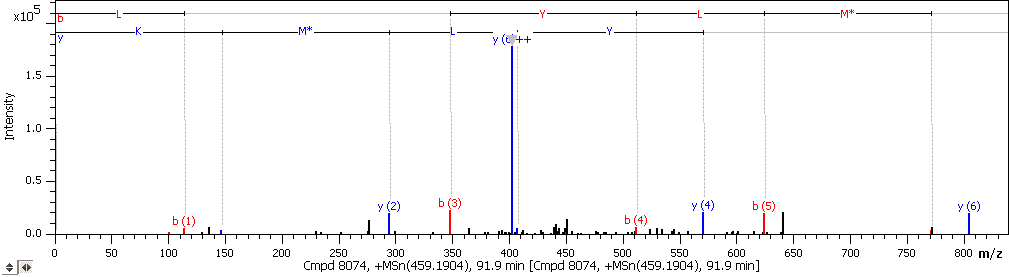


SRSF2


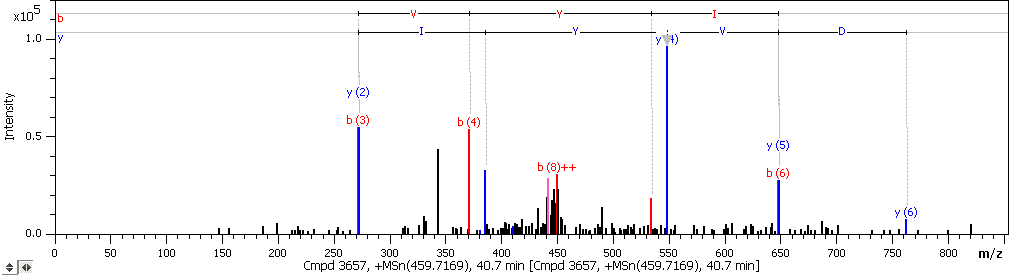


SUMO4


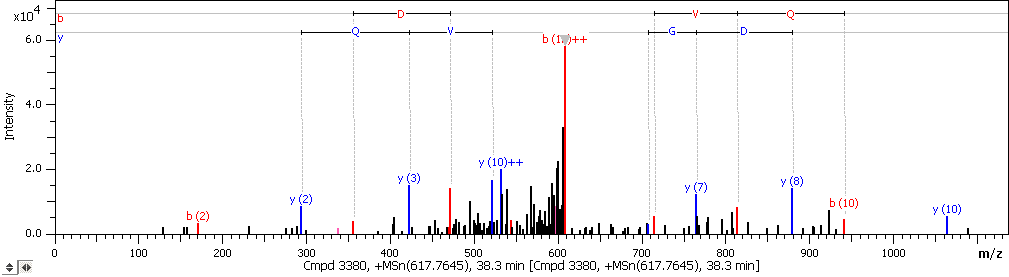


SYK


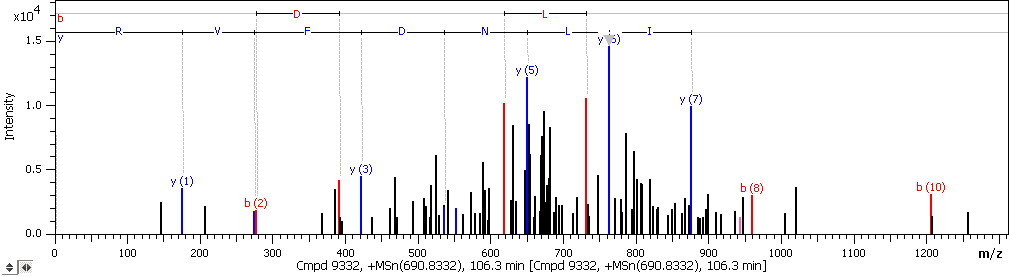


TCP4


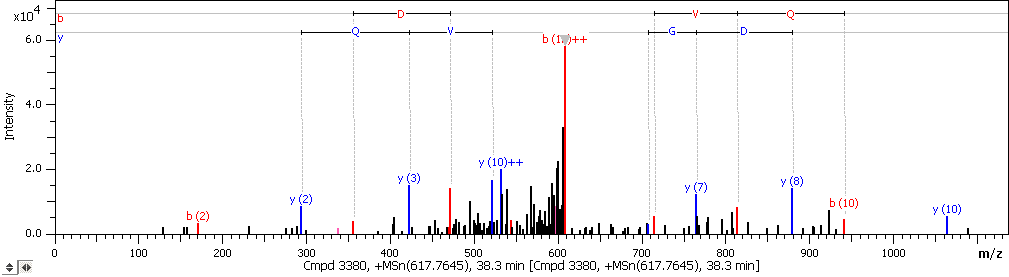


TFAM


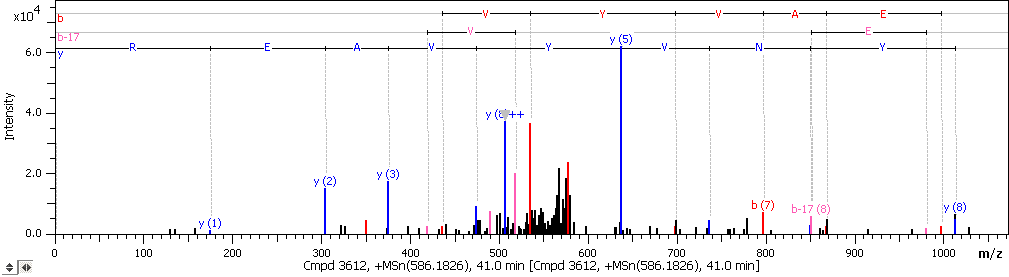


TGFB3


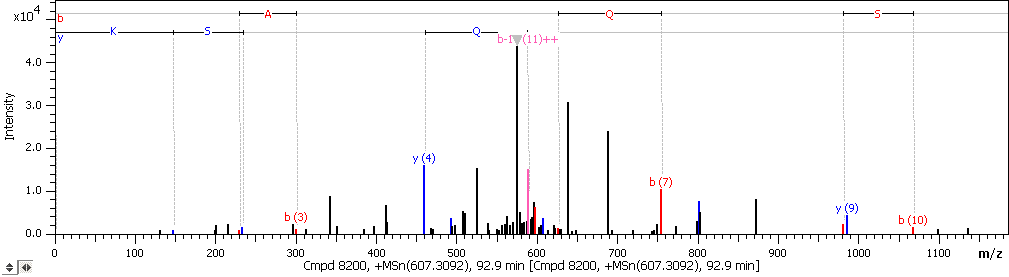


THIC


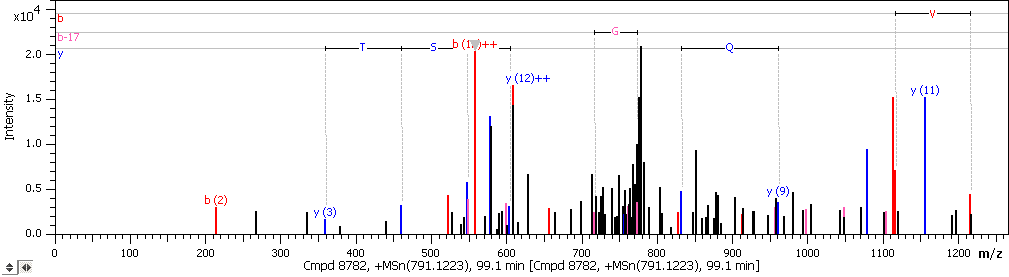


THIM


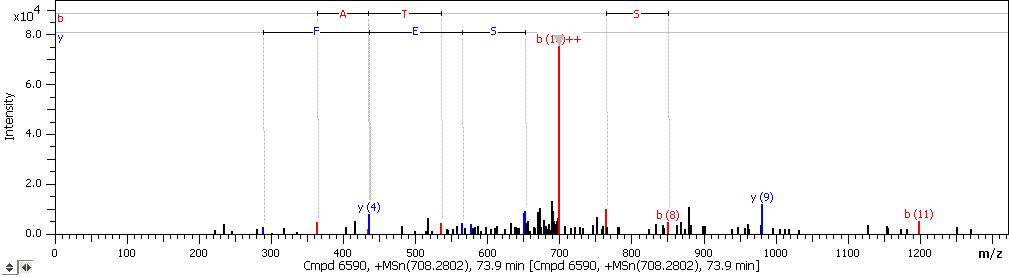


THI0


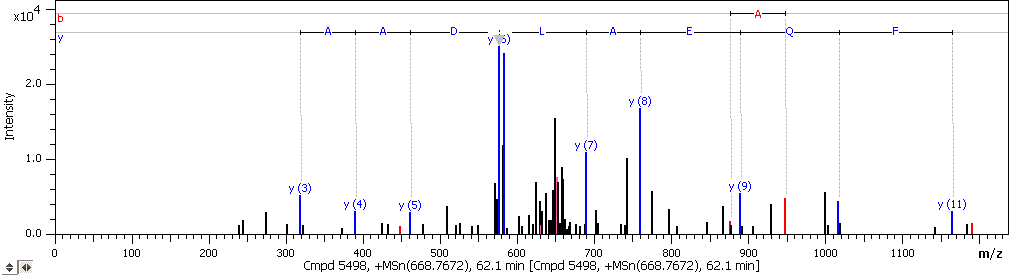


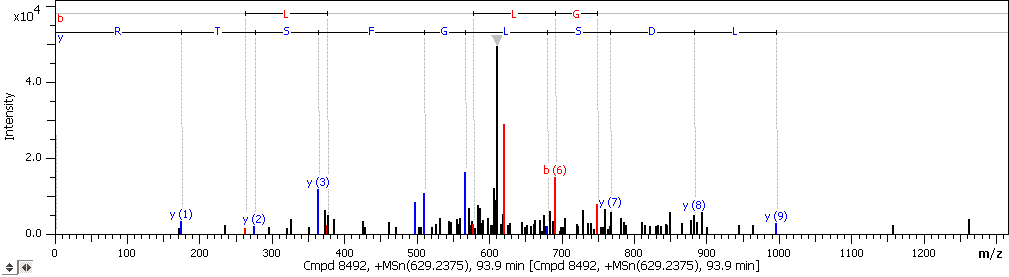


UAP1


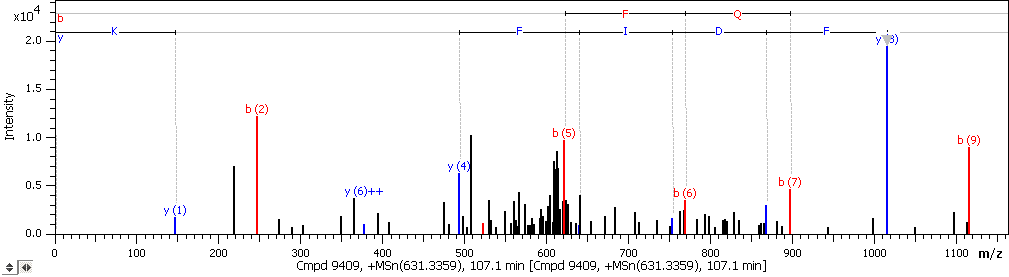


UB2V2


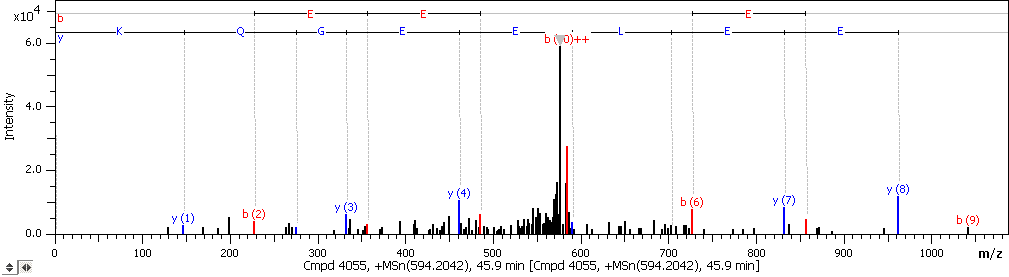


VAMP3


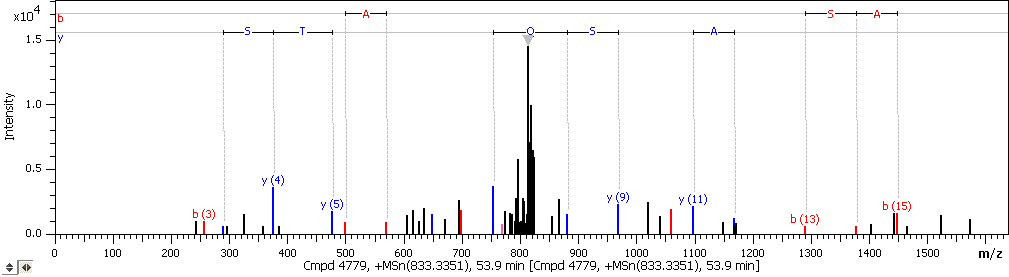


VTA1L


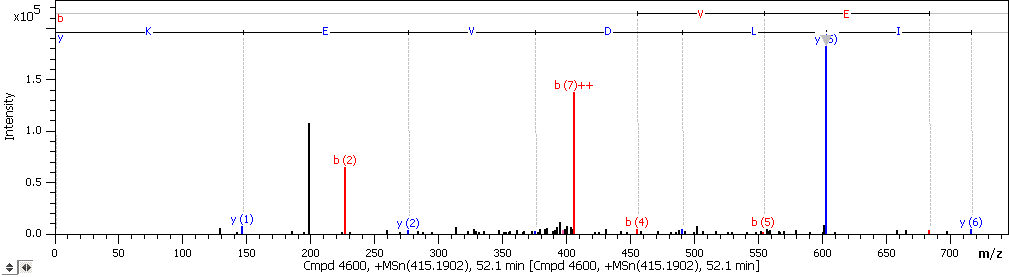


WDR62


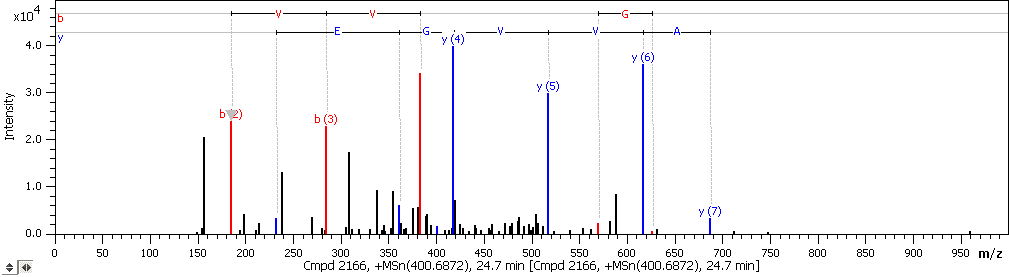

Supplement: Supplementary Information [file cddis2015357x4.docx]
