# Supplementary material for: Affinity purification-mass spectrometry analysis of bcl-2 interactome identified SLIRP as a novel interacting protein
Source: Cell Death Dis. 2016 Feb 11;7(2):e2090–. doi: 10.1038/cddis.2015.357 (PMC4849145; doi:10.1038/cddis.2015.357)
Supplement: Supplementary Table 4 [file cddis2015357x5.docx]

**Supplementary Table 4**. Ingenuity Pathway Analysis Results: List of Significant Molecular and Cellular Function.

| molecular and cellular function | *p-value* | no. of molecules |
| --- | --- | --- |
|  |  |  |
| Molecular Transport | 5.85 X 10^-7^ to 2.30 X 10^-2^ | 51 |
| Protein Trafficking | 5.85 X 10^-7^ to 8.40 X 10^-3^ | 21 |
| Cellular Assembly and Organization | 1.61 X 10^-6^ to 2.21 X 10^-2^ | 56 |
| Nucleic Acid Metabolism | 2.15 X 10^-6^ to 2.30 X 10^-2^ | 23 |
| Small Molecule Biochemistry | 2.15 X 10^-6^ to 2.30 X 10^-2^ | 42 |

**Supplementary Table 5**. Ingenuity Pathway Analysis Results: List of Significant Networks

| network | associated funcion | score |
| --- | --- | --- |
| 1 | Protein Synthesis, Gene Expression, Carbohydrate Metabolism | 53 |
| 2 | RNA Post-Transcriptional Modification, Developmental Disorder, Hereditary Disorder | 53 |
| 3 | Cellular Assembly and Organization, Cellular Compromise, Molecular Transport | 45 |
| 4 | Free Radical Scavenging, Small Molecule Biochemistry, Metabolic Disease | 38 |
| 5 | Cell Death and Survival, Infectious Disease, Cellular Assembly and Organization | 27 |

**Supplementary Table 6**. Ingenuity Pathway Analysis Results: Top Tox Lists

| Name | *p-value* |
| --- | --- |
| Mitochondrial Dysfunction | 7.06E-08 |
| Decreases Depolarization of Mitochondria and Mitochondrial Membrane | 3.47E-06 |
| Increases Transmembrane Potential of Mitochondria and Mitochondrial Membrane | 2.65E-03 |
| Anti-Apoptosis | 5.93E-03 |
| Decreases Transmembrane Potential of Mitochondria and Mitochondrial Membrane | 1.17E-02 |

**Supplementary Table 7**. Ingenuity Pathway Analysis Results: list of Top Canonical Pathways.

| Name | *p-value* |
| --- | --- |
| EIF2 Signaling | 1.64E-10 |
| Mitochondrial Dysfunction | 6.58E-08 |
| Regulation of eIF4 and p70S6K Signaling | 8.19E-07 |
| RhoA Signaling | 1.09E-05 |
| Oxidative Phosphorylation | 2.81E-05 |
