# Supplementary material for: Affinity purification-mass spectrometry analysis of bcl-2 interactome identified SLIRP as a novel interacting protein
Source: Cell Death Dis. 2016 Feb 11;7(2):e2090–. doi: 10.1038/cddis.2015.357 (PMC4849145; doi:10.1038/cddis.2015.357)

BCL2_HUMAN

Apoptosis regulator Bcl-2

| Row | m/z meas. | Mr calc. | z | Δ m/z [ppm] | Scores | Sequence | Modifications | Range |
| --- | --- | --- | --- | --- | --- | --- | --- | --- |
| 1 | 605.7574 | 1209.6394 | 2 | -114.87 | 85 | R.FATVVEELFR.D |  | 130 - 139 |
| 2 | 656.3074 | 1965.9255 | 3 | -12.68 | 47.1 | R.DFAEMSSQLHLTPFTAR.G | Oxidation: 5 | 111 - 127 |


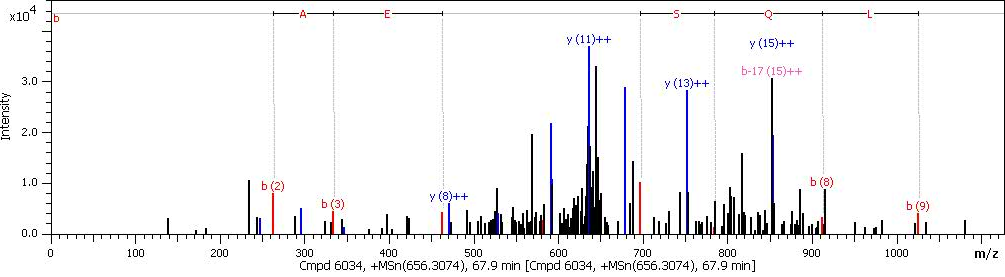


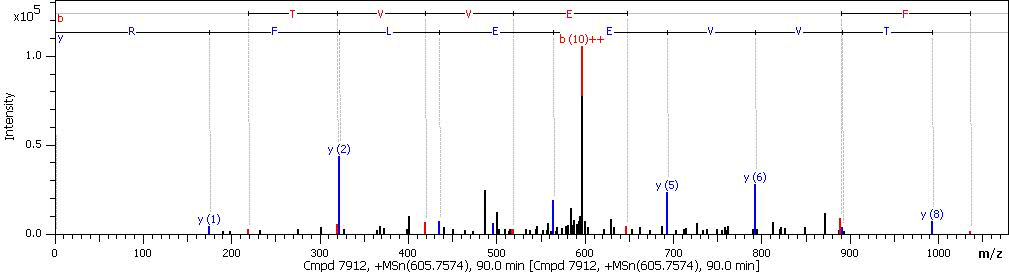


SLIRP_HUMAN

SRA stem-loop-interacting RNA-binding protein

| Row | m/z meas. | Mr calc. | z | Δ m/z [ppm] | Scores | Sequence | Modifications | Range |
| --- | --- | --- | --- | --- | --- | --- | --- | --- |
| 1 | 565.7642 | 1129.6244 | 2 | -97.70 | 44 | R.SINQPVAFVR.R |  | 15 - 24 |


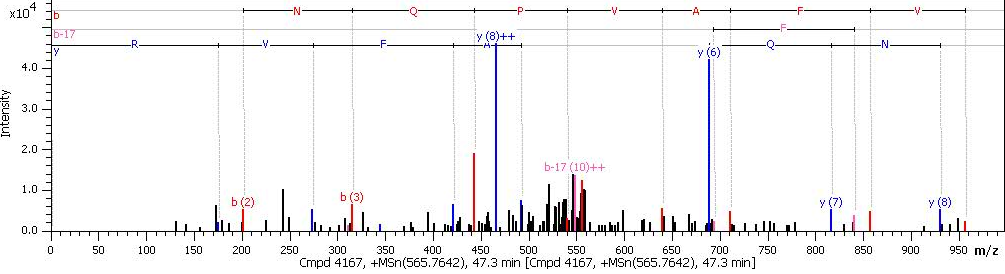


COX5A_HUMAN

Cytochrome c oxidase subunit 5A

| Row | m/z meas. | Mr calc. | z | Δ m/z [ppm] | Scores | Sequence | Modifications | Range |
| --- | --- | --- | --- | --- | --- | --- | --- | --- |
| 1 | 684.9661 | 2052.0105 | 3 | -65.17 | 37.8 | R.WVTYFNKPDIDAWELR.K |  | 56 - 71 |
| 2 | 838.9120 | 1675.8491 | 2 | -23.61 | 31.1 | K.GINTLVTYDMVPEPK.I |  | 73 - 87 |


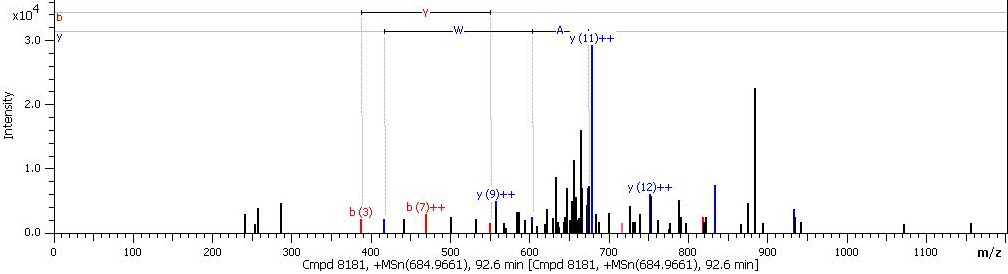


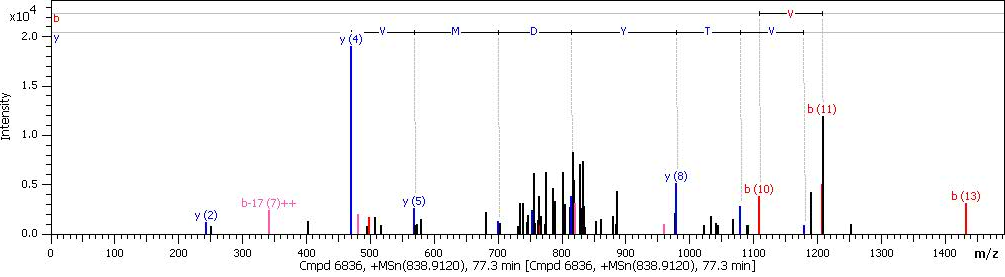


VDAC1_HUMAN

Voltage-dependent anion-selective channel protein 1

| Row | m/z meas. | Mr calc. | z | Δ m/z [ppm] | Scores | Sequence | Modifications | Range |
| --- | --- | --- | --- | --- | --- | --- | --- | --- |
| 2 | 515.7677 | 1029.6070 | 2 | -83.49 | 87.4 (M:87.4) | K.LTLSALLDGK.N |  | 257 - 266 |
| 3 | 607.2867 | 1212.6139 | 2 | -45.27 | 75.4 (M:75.4) | R.VTQSNFAVGYK.T |  | 164 - 174 |
| 4 | 687.7910 | 1373.6503 | 2 | -60.20 | 51.8 (M:51.8) | R.WTEYGLTFTEK.W |  | 64 - 74 |
| 5 | 700.7989 | 1399.6620 | 2 | -56.19 | 78.8 (M:78.8) | K.LTFDSSFSPNTGK.K |  | 97 - 109 |
| 6 | 701.7261 | 2102.1736 | 3 | -8.07 | 60.0 (M:60.0) | K.VNNSSLIGLGYTQTLKPGIK.L |  | 237 - 256 |
| 7 | 707.7864 | 1413.6235 | 2 | -46.11 | 56.2 (M:56.2) | K.YQIDPDACFSAK.V | Carbamidomethyl: 8 | 225 - 236 |


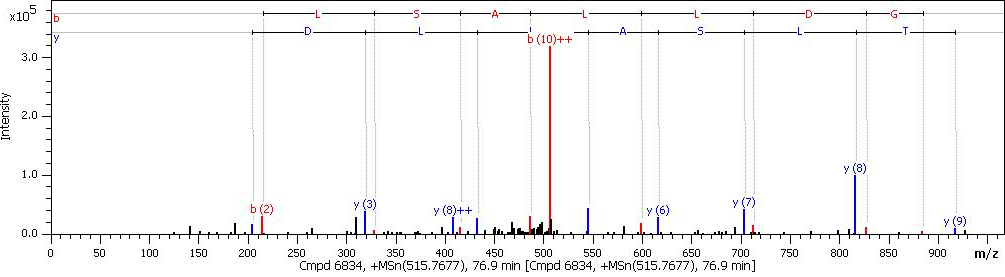


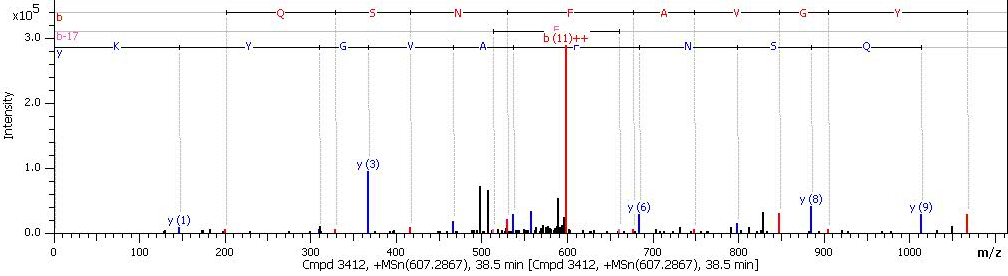


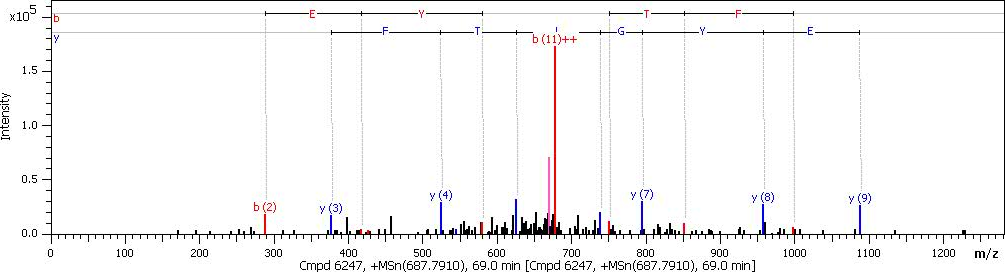


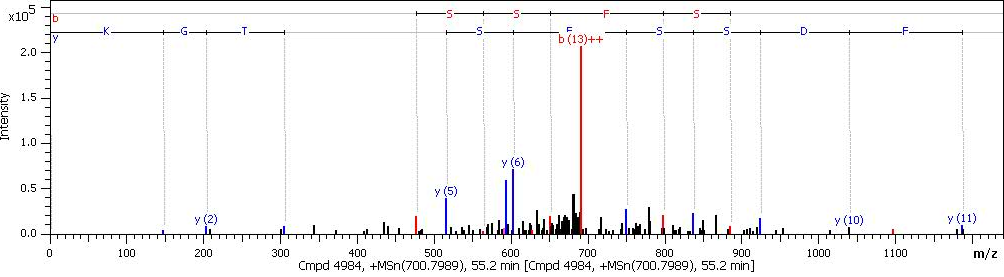


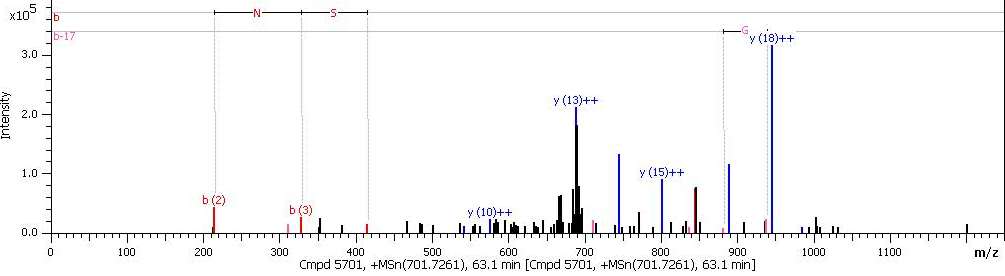


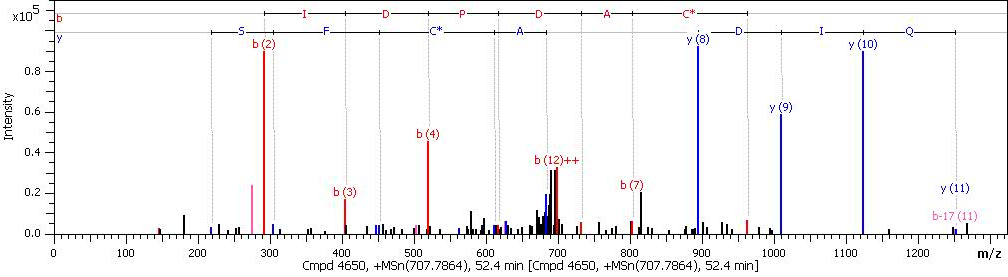

Supplement: Supplementary Information [file cddis2015357x6.docx]
