# Supplementary material for: Affinity purification-mass spectrometry analysis of bcl-2 interactome identified SLIRP as a novel interacting protein
Source: Cell Death Dis. 2016 Feb 11;7(2):e2090–. doi: 10.1038/cddis.2015.357 (PMC4849145; doi:10.1038/cddis.2015.357)
Supplement: Supplementary Table 7 [file cddis2015357x7.docx]

**Supplementary Table 7.** List of primers used in qRT-PCR

| Primer | Sequence (5'-3') |
| --- | --- |
| SLIRP forward | GCGCTGCGTAGAAGTATCAA |
| SLIRP reverse | TGCATTCCGAAGTCCTTCTT |
| ND1 forward | GAGCAGTAGCCCAAACAATCTC |
| ND1 reverse | GGGTCATGATGGCAGGAGTAAT |
| mtCOX1 forward | GGAGCAGGAACAGGTTGAACAG |
| mtCOX1 reverse | GTTGTGATGAAATTGATGGC |
| ATPase8/6 forward | CACAACACTAAAGGACGAACCT |
| ATPase8/6 reverse | GGGATGGCCATGGCTAGGTTTA |
| CYTB forward | CTGATCCTCCAAATCACCACAG |
| CYTB reverse | GCGCCATTGGCGTGAAGGTA |
| ND5 forward | ACCGCACAATCCCCTATCTAGG |
| ND5 reverse | TTGGGTTGAGGTGATGATGGAG |
| ND6 forward | TGGGGTTAGCGATGGAGGTAGG |
| ND6 reverse | AATAGGATCCTCCCGAATCAAC |
| ND3 forward | CCACCCCTTACGAGTGCGGCTT |
| ND3 reverse | TTGTAGGGCTCATGGTAGGGGT |
| actin forward | ATTGCCGACAGGATGCAGAA |
| actin reverse | GCTGATCCACATCTGCTGGAA |
